# Probing sulfatide-tissue lectin recognition with functionalized glycodendrimersomes

**DOI:** 10.1016/j.isci.2020.101919

**Published:** 2020-12-10

**Authors:** Paul V. Murphy, Antonio Romero, Qi Xiao, Anna-Kristin Ludwig, Srinivas Jogula, Nadezhda V. Shilova, Tanuja Singh, Adele Gabba, Bilal Javed, Dapeng Zhang, Francisco J. Medrano, Herbert Kaltner, Jürgen Kopitz, Nicolai V. Bovin, Albert M. Wu, Michael L. Klein, Virgil Percec, Hans-Joachim Gabius

**Affiliations:** 1 CÚRAM – SFI Research Centre for Medical Devices and the School of Chemistry, National University of Ireland Galway, University Road, Galway H91 TK33, Ireland; 2 Department of Structural and Chemical Biology, CIB Margarita Salas, CSIC, Ramiro de Maeztu, 9, 28040 Madrid, Spain; 3 Institute of Computational Molecular Science, Temple University, Philadelphia, PA 19122, USA; 4 Roy & Diana Vagelos Laboratories, Department of Chemistry, University of Pennsylvania, Philadelphia, PA 19104-6323, USA; 5 Institute of Physiological Chemistry, Faculty of Veterinary Medicine, Ludwig-Maximilians-University Munich, Veterinärstr. 13, 80539 Munich, Germany; 6 Shemyakin & Ovchinnikov Institute of Bioorganic Chemistry, Russian Academy of Sciences, 16/10 Miklukho-Maklaya str., 117437 Moscow, Russian Federation; 7 National Medical Research Center for Obstetrics, Gynecology and Perinatology named after Academician V.I. Kulakov of the Ministry of Healthcare of Russian Federation, 4 Oparina str, 117997 Moscow, Russian Federation; 8 Glyco-Immunology Research Laboratory, Institute of Molecular and Cellular Biology, Chang-Gung-Medical College, Kwei-san, Tao-yuan 333, Taiwan; 9 Zentrum Pathologie, Institut für Angewandte Tumorbiologie, Medizinische Fakultät der Ruprecht-Karls-Universität Heidelberg, Im Neuenheimer Feld 224, 69120 Heidelberg, Germany

**Keywords:** Supramolecular Chemistry, Biochemistry, Biophysics

## Abstract

The small 3-*O*-sulfated galactose head group of sulfatides, an abundant glycosphingolipid class, poses the (sphinx-like) riddle on involvement of glycan bridging by tissue lectins (sugar code). First, synthesis of head group derivatives for functionalization of amphiphilic dendrimers is performed. Aggregation of resulting (biomimetic) vesicles, alone or in combination with lactose, demonstrates bridging by a tissue lectin (galectin-4). Physiologically, this can stabilize glycolipid-rich microdomains (rafts) and associate sulfatide-rich regions with specific glycoproteins. Further testing documents importance of heterobivalency and linker length. Structurally, sulfatide recognition by galectin-8 is shown to involve sphingosine's OH group as substitute for the 3′-hydroxyl of glucose of lactose. These discoveries underscore functionality of this small determinant on biomembranes intracellularly and on the cell surface. Moreover, they provide a role model to examine counterreceptor capacity of more complex glycans of glycosphingolipids and to start their bottom-up glycotope surface programming.

## Highlights

• Nanoparticle programming detects sulfatide-(*N*)-glycan bridging by galectins-4 and -8
• Protein design (linker/domain type) is a switch for aggregation activity
• Sphingosine's OH group is involved in contact building with a galectin

## Graphical abstract

**Figure:**
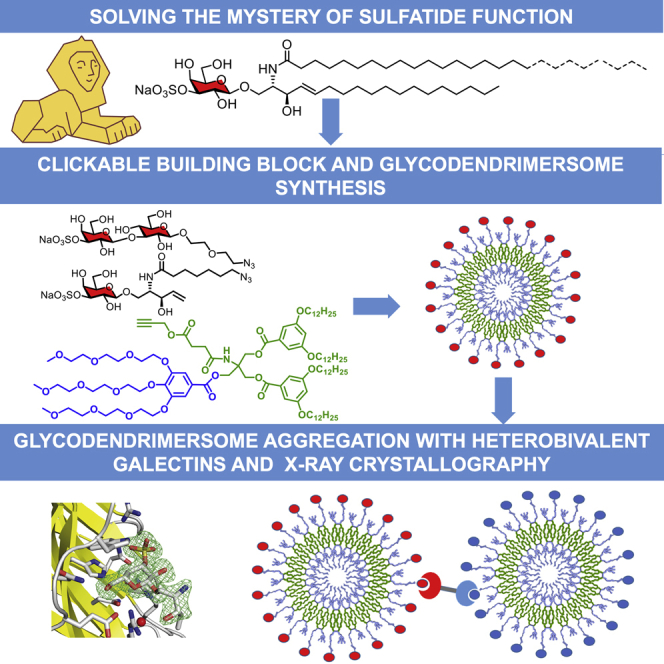

## Introduction

The “many enigmas” around a major component of “alkaloidal nature” in ethanolic brain extracts, i.e. sphingosine, and the sphingolipids are symbolized by their names: they originate from the sphinx and its famous riddle (Sourkes, 2003; Thudichum, 1884). Thudichum's detection of neutral sugar in phrenosine, now called galactocerebrosides, and its 3-*O*-sulfated derivative in sulfatides has started efforts to explain the abundant presence of a lipid-linked monosaccharide and its site-specific sulfation. Of course, it is reasonable to assume that these simple compounds are more than inert constituents of the lipid bilayer (Ishizuka, 1997; Takahashi and Suzuki, 2012; Vos et al., 1994; Yamakawa et al., 1962). As an attractive possibility, the concept of the sugar code considers the glycan part of glycosphingolipids as a biochemical message anchored in the membrane, and thereby presented on its surface ready for being ‘read’ by receptors (Gabius and Roth, 2017; Kaltner et al., 2019). Herein, we focus on the head group of sulfatides, whose enzymatic synthesis is made possible by (galactosyl)cerebroside 3-*O*-sulfotransferase (CST, Gal3ST-1). The occurrence of further sulfotransferases acting on sugars and their diversification to gain selectivity for diverse substrates are the pillars of the hypothesis for (patho)physiological relevance of glycan sulfation, which is assumed to convert rather small glycans (even a monosaccharide) into potent ligands (Bowman and Bertozzi, 1999; Fukuda et al., 2001; Hemmerich and Rosen, 2000; Hooper et al., 1997).

**Figure 1:**
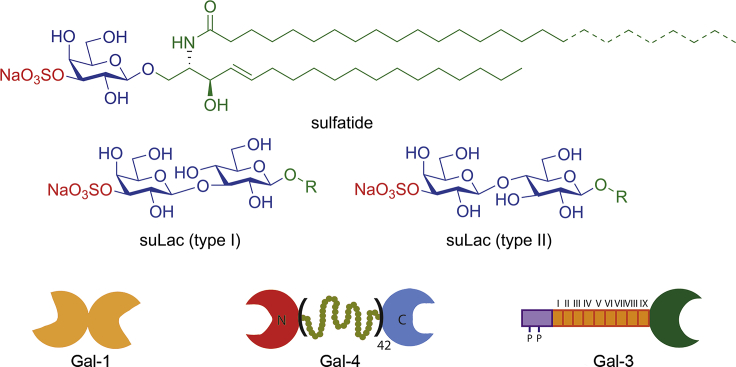
The toolbox of our study The carbohydrate ligands and the three forms of architecture of human galectins, i.e. non-covalently associated homodimer, linker-connected heterodimer (galectin-4 [Gal-4] with a 42 amino-acid-long linker) and lectin domain with N-terminal tail containing collagen-like repeats and a peptide with two sites of serine phosphorylation.

With respect to 3-*O*-sulfation of galactose, more than one enzyme with this activity has evolved. In addition to sulfatide generation, galactose of *N*-acetyllactosamine (Lac*N*Ac) at branch ends of glycan chains after protein glycosylation is a substrate (for Gal-3ST-2/-3; products shown in Figure 1) (Fukuda et al., 2001). In comparison to the advanced status of the characterization of the enzymatic machinery for the 3-*O*-sulfation of galactose, precise elucidation of the actual profile of bioactivities of the products as ligand lags behind. Respective efforts would benefit from a testing with a chemically prepared sulfatide analog and a fully programmable model system, tailored to be in principle useful for any type of glycosphingolipid ensuring broad-scale applicability. It is a challenge for synthetic and supramolecular chemistry to create the respective toolbox to put the assumption of involvement of the sulfatide head group in cross-linking processes by tissue receptors to the test.

Like the glycosphingolipids, known endogenous receptors for their glycan part (lectins) present their own mysteries (Ledeen et al., 2018). Especially the occurrence of diverse types of modular architecture is not yet fully understood in functional terms. As illustrated in the bottom part of Figure 1 for vertebrate galectins, three distinct forms are found (García Caballero et al., 2020; Kaltner et al., 2017). Concerning sulfatides as ligands, binding has up to now been reported for human galectins-4 and -8 (Gal-4 and -8) using the glycosphingolipid adsorbed to a plastic surface, the interaction with Gal-4 on the cellular level implicated in stabilization of enterocyte membrane microdomains rich in (glyco)lipids and -proteins (lipid rafts, also known as a fundamental platform for starting outside-in signaling) and in sulfatide-dependent apical/axonal routing of distinct glycoprotein cargo (likely predestined for recognition by a high density of Lac*N*Ac of complex-type *N*-glycans) (Braccia et al., 2003; Danielsen and van Deurs, 1997; Delacour et al., 2005; Ideo et al., 2003; 2005; Morelle et al., 2009; Stechly et al., 2009; Velasco et al., 2013). However, a physical bridging required for these processes has not yet been demonstrated. As shown by array testing with glycans and by galectin-dependent cell association of neoglycoconjugates (Blixt et al., 2004; Vokhmyanina et al., 2012), as well as by cocrystallization of both CRDs of Gal-4 with oligosaccharides (Bum-Erdene et al., 2015, 2016), the apparent dual specificity of Gal-4 to neutral and to sulfated glycans will have an obvious consequence for attempts to show functional bivalency and bridging: it requires to take assay and nanoparticle designs from a single defined epitope to mixed systems, hereby establishing the starting point for bottom-up tailoring to eventually reproduce cellular biodiversity. In detail for this context, the components for assays are built to simulate the natural presentation of substituted (sulfatide) and unsubstituted (glycoprotein) β-galactosides; this way, chemical proof-of-principle tools for examining the possibility of an actual realization of dual specificity of Gal-4 *in vivo* are established.

Here, we describe preparation of conjugatable headgroups and their alkyne-functionalized adapter for linking sulfatide derivatives to a lipid anchor to prepare custom-made amphiphilic Janus glycodendrimersomes (GDSs), which made galectin testing (incl. architecture variants obtained by protein engineering) possible: this strategic combination is applied as a step to solve pertinent mysteries both on (ga)lectin/glycosphingolipid presence and on structural diversity of galectins. The advantage of proof-of-principle work with amphiphilic Janus glycodendrimers, besides the perspective for chemical programming of the nanoparticle surface, is gaining access to diverse morphologies relevant for pathobiology such as cubosomes (Xiao et al., 2016a) or for galectin secretion, that is onion (multivesicular body)-like GDSs (Xiao et al., 2016b). Of course, application of synthetic glycodendrimers in classical systems such as liposomes will also be possible.

After having described the procedures to obtain the suited conjugatable head group derivatives and to nanoparticles as well as after having detected and mapped their activity profile in *trans*-interactions by aggregation assays, we also probed into the structural basis of contact building on the atomic level. This process commonly requires extension of galactose to the disaccharide (lactose) for galectins so that the glucose moiety can contribute, as revealed for the galectin CRD by crystallography (Kamitori, 2018; Romero and Gabius, 2019) and by chemical mapping (Solís et al., 1996). The data obtained by crystallography with the synthetic head group are first evidence that a non-glycan determinant, here sphingosine's hydroxyl group, is involved in sulfatide pairing with a galectin via water-mediated contacts, as the 3-OH group of glucose of the canonical ligand lactose otherwise does by direct hydrogen bonding.

## Results and discussion

### Sulfated lactose as ligand

**Scheme 1:**
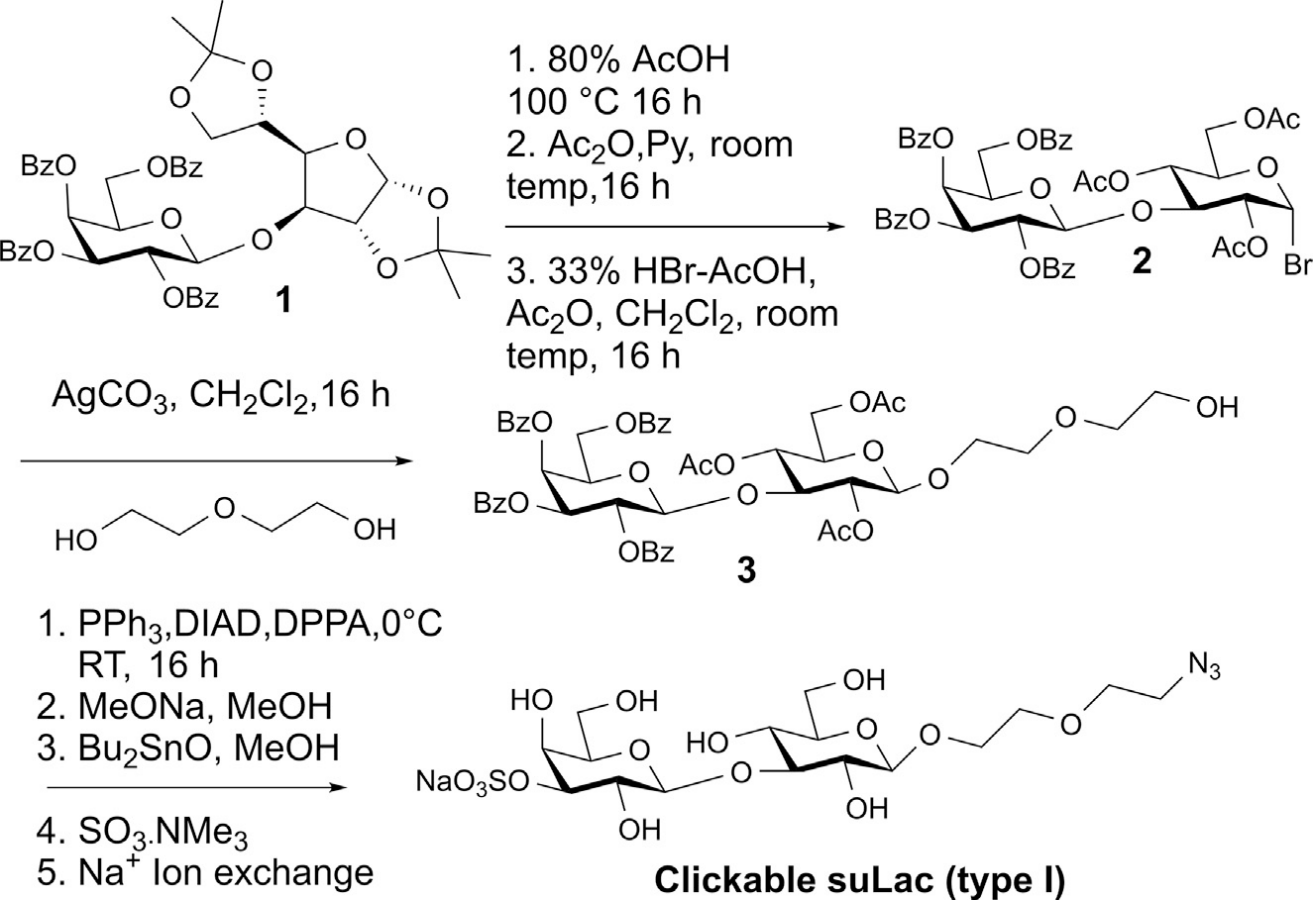
Synthetic route to the clickable suLac (type I) derivative See also Figure S3.

As a step to elucidate ligand properties of sulfated galactose comparatively, we first prepared *O*-sulfated galactose in β1,3-linkage to glucose (termed suLac (type I)). Its synthesis starting from galactopyranoside **1** (Crich et al., 2005) is summarized in Scheme 1.

The attachment of the azide-bearing glycan derivative to the lipid anchor alkyne by click chemistry (Percec et al., 2013; Zhang et al., 2014) and the resulting product shown in Figure S1 (left) self-assembled into GDSs (D_DLS_ = 64 nm, PDI = 0.27). Their application in aggregation assays documented that both CRDs in the heterobivalent Gal-4 can associate to this type of ligand, hereby bridging the suLac (type I)-bearing GDSs (Figure 1). A single CRD or a CRD mixture is unable to connect particles, highlighting the need for integrity of the linker. Endpoint determination indicates a reduced OD value relative to testing suLac (type II) (Xiao et al., 2018). In order to examine the importance of the nature of the linker between the two CRDs in this galectin architecture, we produced variants with shortened sequence or without such extension. Intriguingly, not length reduction (see Gal-4V with 16 amino acids instead of 42 amino acids in the linker in Figure 1, bottom center) but the complete removal of the linker between the CRDs by cDNA engineering visibly decreased extent of aggregation (see Gal-4P in Figure S1 ). Since profiles of array binding appear influenced by the nature of the CRD and also the length of the linker (Figure S2), the results of these assays inform us about notable consequences of reduction of the length of the linker. Since proto-type galectins form homodimers by non-covalent association, their testing will answer the question on differences in bridging capacity.

Interestingly, homodimeric (linker-free) Gal-1, -2 and -7 are active, pointing to a combination of nature of CRD and architecture of its presentation for extent of activity (Figure S1, right). The relative signal intensities for Gal-1 and -8 were similar, as independently reported from using AlphaScreen technology (Tu et al., 2013). Equally important, the chimera-type Gal-3 (Figure 1, bottom-right; monomeric in solution) becomes an aggregant for this ligand, whereas Lac presentation is not sufficient to trigger bridging capacity (Figure S1, right). 3-*O*-sulfated galactose presented by Lac thus is a galectin ligand, tuning of activity occurs by the linker for Gal-4. This documented, we can next address the open question whether cross-linking of ligands on opposing surfaces can still occur after shortening the canonical ligand Lac to the monosaccharide as is the case in sulfatides: only then could Gal-4 bring sulfatides on neighboring rafts as well as sulfatide (preferentially with C24 acyl chain) in such microdomains and glycoprotein cargo with Lac*N*Ac (or suLac) termini together physiologically.

### The sulfatide head group as ligand

**Scheme 2:**
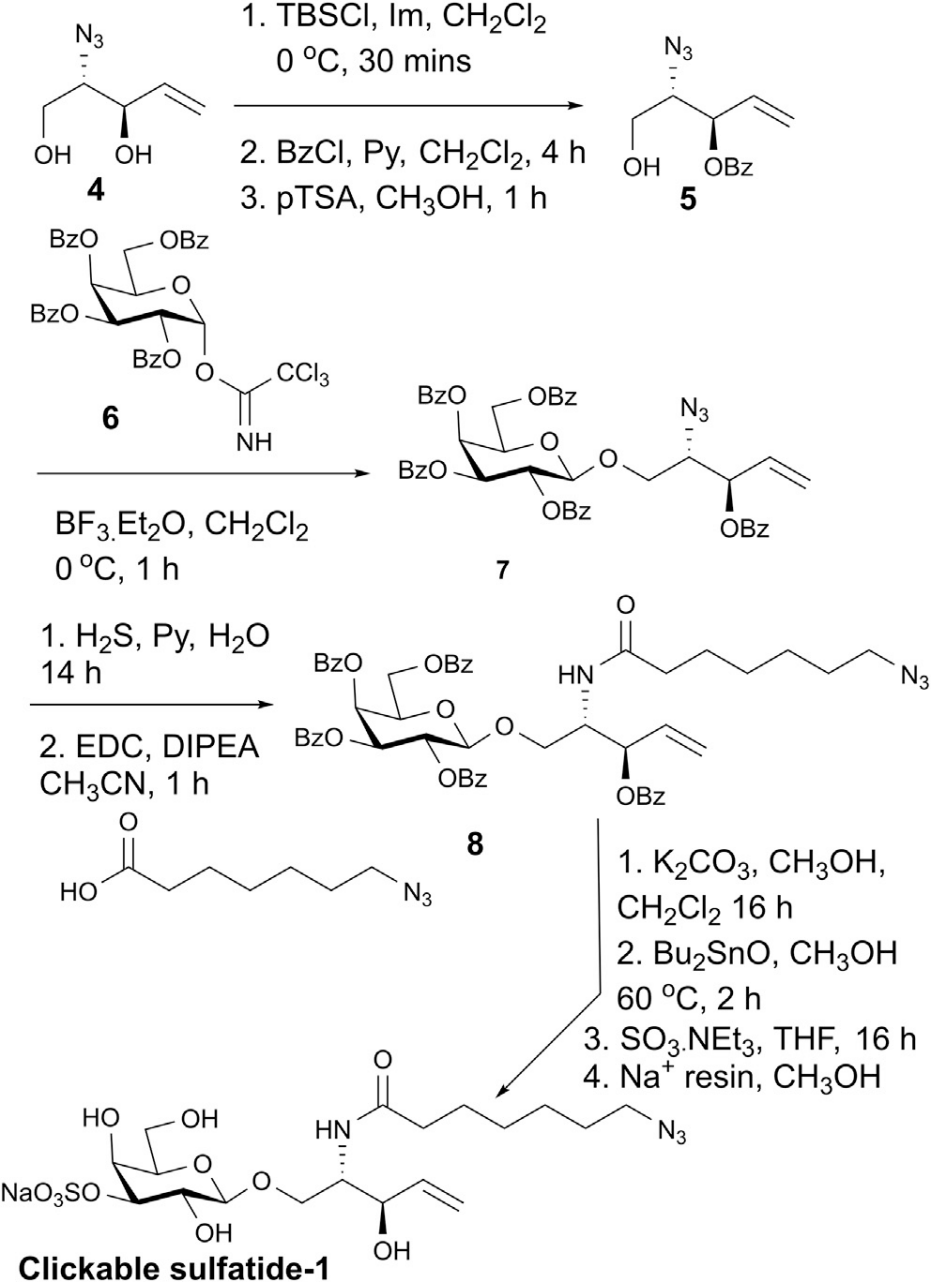
Synthetic route to the clickable sulfatide-1 derivative See also Figure S3.

The structure of the sulfatide hinge region to the acyls (Figure 1, top) suggests two sites for placement of the azide, i.e. in the palmitoyl CoA-derived acyl or in the second acyl (physiologically of variable length, which explains diversity for sulfatides; a C24 chain ensures accessibility). Each of the two routes was taken. The diol **4** described in a patent by (Bundle et al., 2003) with an additional azide group was converted to sulfatide-1 by the steps given in Scheme 2.

**Scheme 3:**
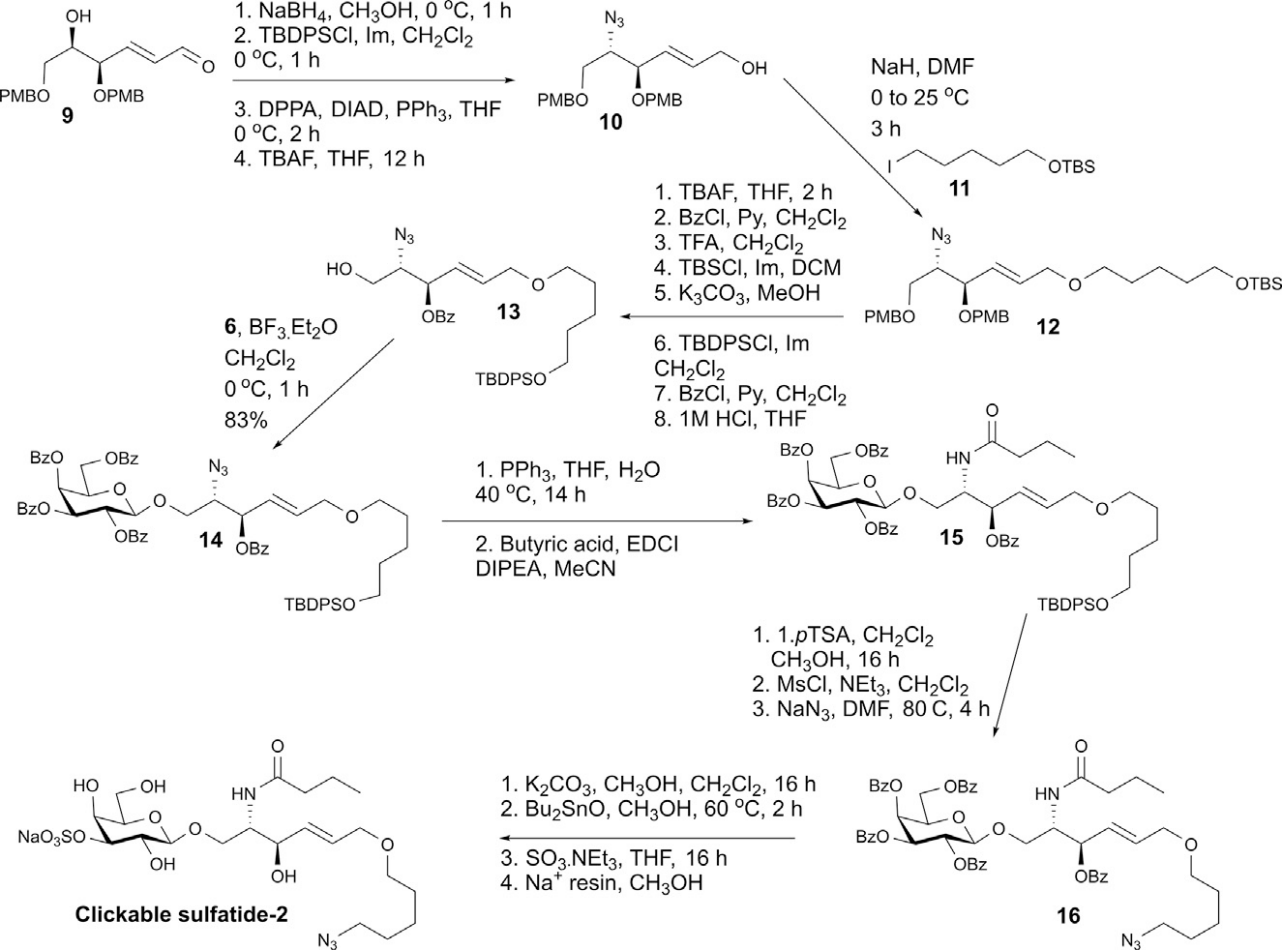
Synthetic route to the clickable sulfatide-2 derivative See also Figure S3.

Alternatively, the clickable azide was introduced into the core structure at the second acyl by the pathway from **9** summarized in Scheme 3.

**Figure 2:**
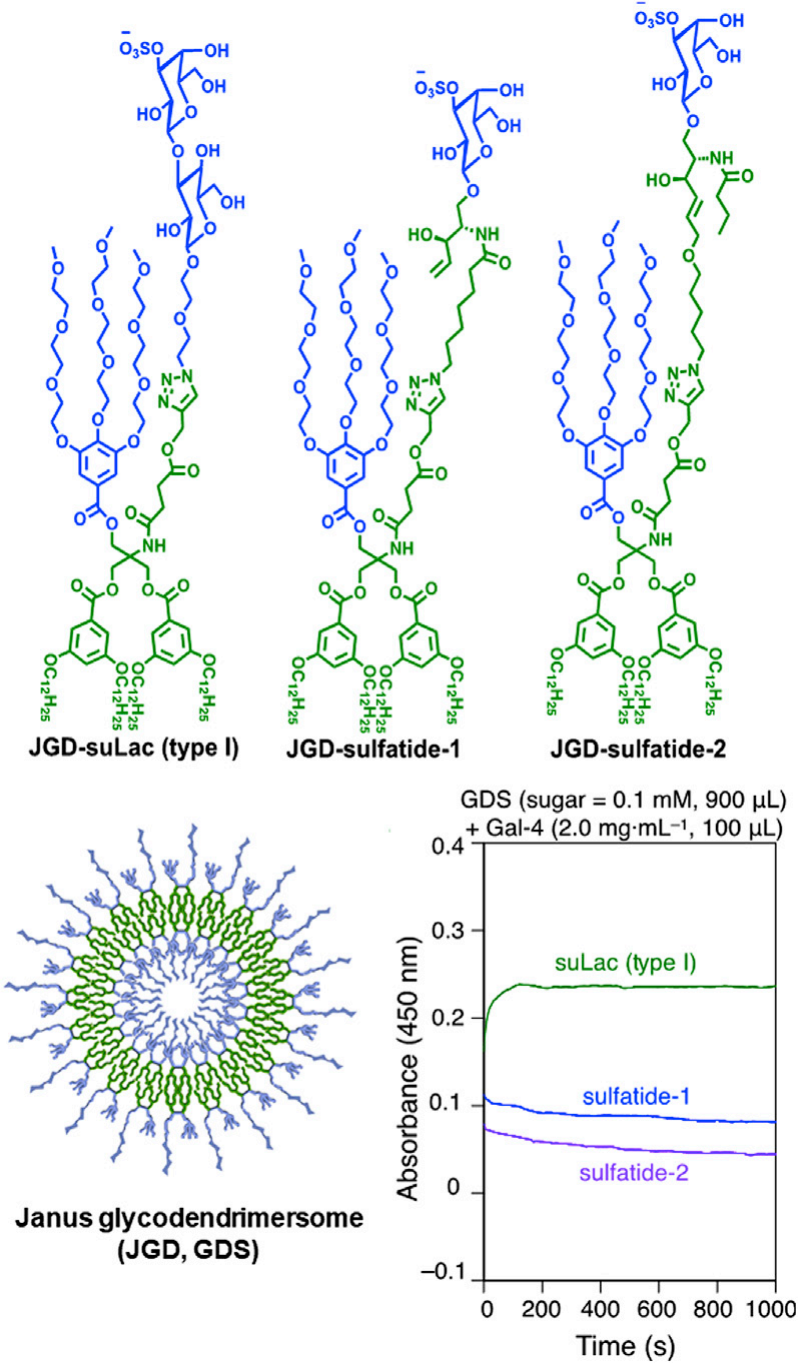
Glycodendrimer and Janus GDS structures (top, bottom-left) and course of aggregation of GDSs by Gal-4 in PBS (pH = 7.4) (bottom-right) See also Figure S3.

The two products were connected to the standard lipid anchor to set the stage for their self-assembly (Figure S3). The GDSs subsequently prepared, using either sulfatide-1 (D_DLS_ = 108 nm; PDI = 0.18) or sulfatide-2 (D_DLS_ = 147 nm; PDI = 0.27) (Figure 2, bottom-left), present a surface mimicking (*cum grano salis*) sulfatide presence in detergent-resistant membrane sections. Remarkably, these artificial nanoparticles established the assay platform to explore sulfatide activity as ligand in our study. Compared with suLac (type I), their ligand activity for Gal-4 was rather small but clearly detectable (Figure 2). The previously reported occurrence of “superrafts” heavily enriched in galectin-4 in microvillar membranes of pig small intestine (Braccia et al., 2003) could thus originate from sulfatide-Gal-4 cross-linking in regions of mutually high density.

**Figure 3:**
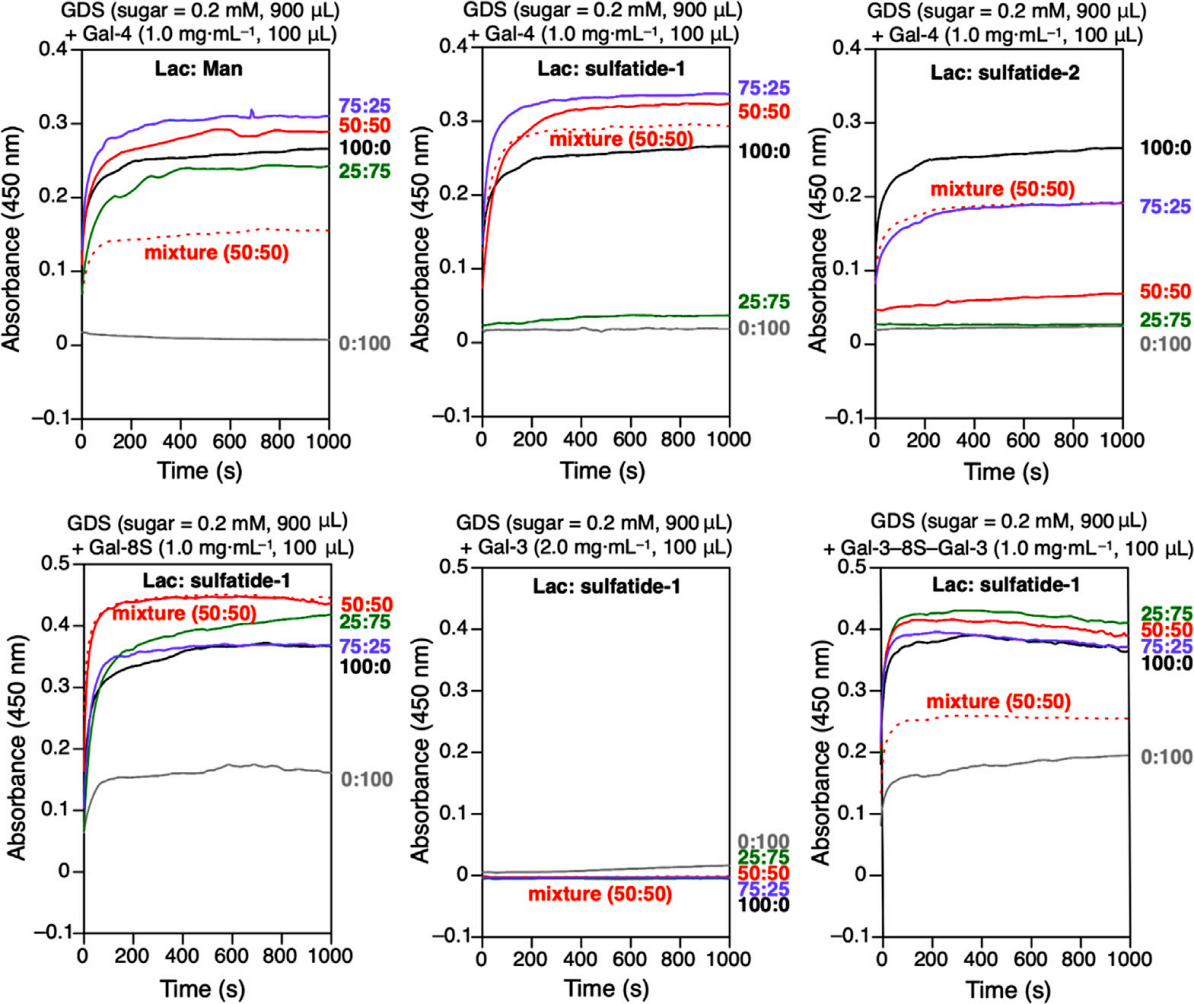
Galectin- and glycan-dependent bridging of GDSs Course of aggregation of co-assembled GDSs by Gal-4, Gal-8S, Gal-3, and the linker-connected homodimeric Gal-3 variant (termed Gal-3-8S-Gal-3) in PBS (pH = 7.4). (See also Figure S3).

Since Gal-4 had also been implicated in arranging glycoprotein segregation by *trans*-interactions, that is to bridge sulfatide and Lac*N*Ac of clustered *N*-glycans of distinct glycoproteins, then extent of galectin-dependent aggregation in mixed systems (a GDS mixture or GDSs obtained from two glycodendrimers mixed at different ratio) will be a respective sensor. The inert sugar mannose (Man) serves as negative control (Figure 3A). The illustrated baseline OD_450_-reading excludes a cognate-carbohydrate-independent aggregation, a general stickiness of ethylene glycol being the concern. The experience with glycoclusters containing similar linker structures, e.g. reported by (André et al., 2009, 2010), with a PEGylated galectin that does not self-associate, even exerting repulsion (He et al., 2010) and with other types of galectins and sugars of graded interaction potential (Ludwig et al., 2019a; Zhang et al., 2015) builds a solid body of evidence against such unwanted side effects. Moreover, a contact site found for glycerol in the N-terminal domain of murine Gal-4 is spatially distinct from the surface pocket binding lactose (Krejciríková et al., 2011), allowing them to distinguish bindings by competitive inhibition.

Figure 3 presents evidence for increased extent of aggregation of nanoparticle mixtures for the pair of Gal-4 and sulfatide-1-bearing GDSs (top). Presence of the sulfatide head groups increased the threshold for aggregation relative to Man when testing GDSs obtained from glycodendrimer mixtures (Figure 3, top). Physiologically, the density of Lac*N*Ac termini on *N*-glycans of a glycoprotein can thus be a criterion for counterreceptor selection, what has actually been suggested (Morelle et al., 2009). The data of our panel testing of glycoproteins (Tables S1 and S2, and Figure S4) support this concept.

Having revealed physical contacts in this heterotypic (pseudophysiological) system, the question can be addressed next as to whether Gal-4 is unique for this interplay. Respective testing disclosed aggregation also for Gal-8 (Figure 3, bottom, left). Wild-type Gal-3 that effectively connects suLac head groups (see above) fails to do the same with sulfatide. Of particular note, the Gal-3 CRD yet becomes an active cross-linker after its engineering to a homodimer with a linker (Figure 3, bottom row): obviously, the protein design is crucial and its alteration by engineering is like a molecular switch. Homodimeric Gal-1, in contrast, shows no evidence for a bridging activity of sulfatide headgroups (Figure S5). The loss of contact to the Glc(*N*Ac) part of suLac(*N*Ac) may play a significant role here, when noting the respective capacity of suLac as docking point. The two tandem-repeat-type galectins thus appear to use a compensatory contact for acquiring stability, and here sphingosine's hydroxyl group comes into play as a possibility. A means to test the validity of this hypothesis is crystallographic study, if a complex of a galectin CRD with synthetic sulfatide-1 could be obtained.

### Crystallography of Gal-8N with a sulfatide head group

**Figure 4:**
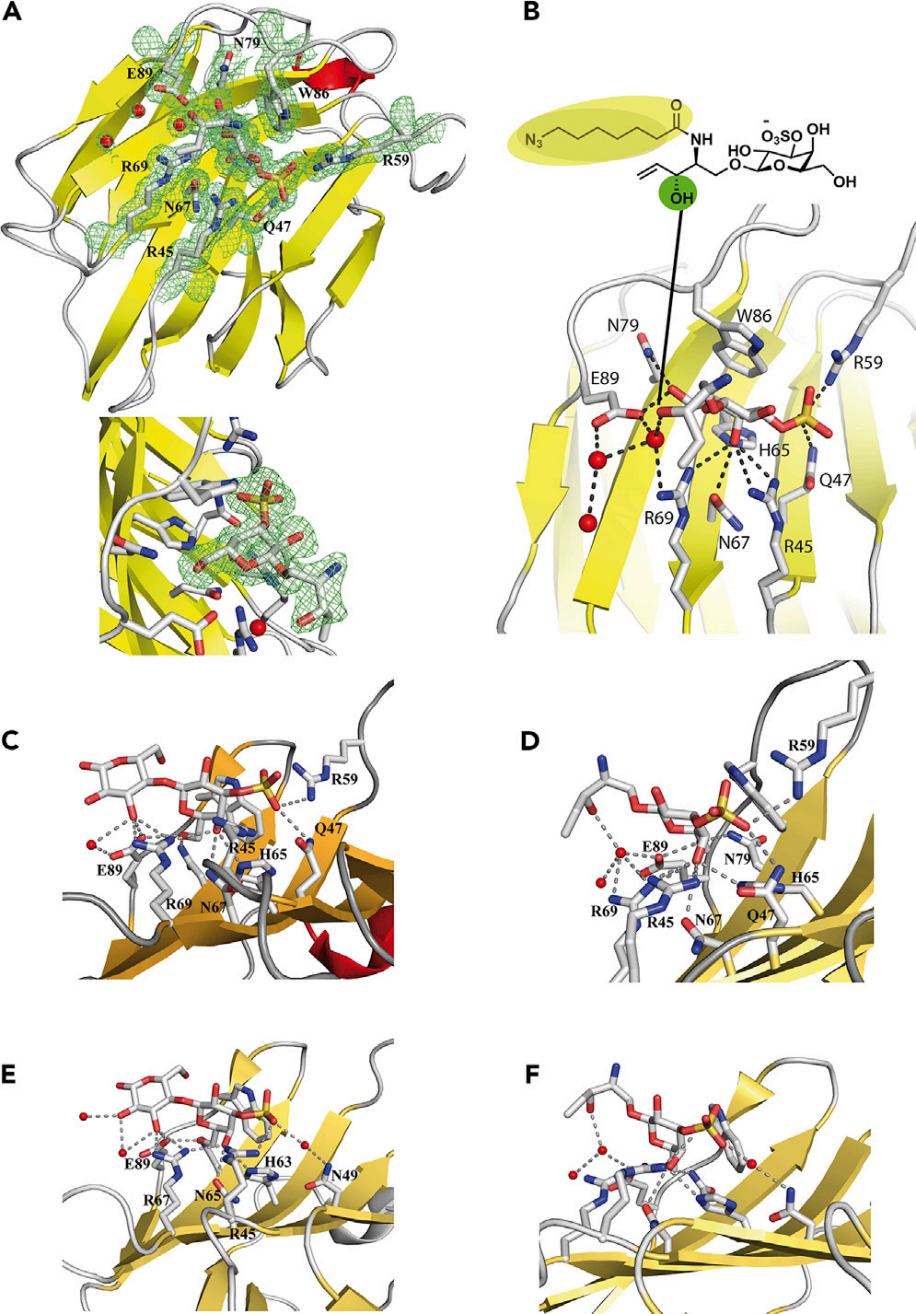
Structural equivalence of hydroxyl groups of glucose/sphingosine (A–F) (A) Relevant section of Gal-8N complexed with the sulfatide-1 as 2Fo-Fc electron density map contoured at 1.0 σ (protein drawn in cartoon-style, sulfatide-1 and contact residues in ball-and-stick mode and water molecules red spheres) at two levels of magnification and (B) profile of interactions, highlighting sphingosine's OH group and the mobile acyl section of the amide. Comparative illustration of the relevant sections of Gal-8N-ligand complexes for suLac (PDB: 3AP6; C) and for sulfatide-1 (D) showing the structural equivalence of the hydroxyl groups of sugar (C) and sphingosine (D). This is also seen for Gal-4N-ligand complexes, i.e. Gal-4-N with suLac (PDB: 5DUW); (E) and the correspondingly modeled complex with sulfatide-1 (F).

Systematic testing of conditions with the N-terminal CRDs of Gal-4 and -8 led to crystals for Gal-8N and thus data to decide the issue (Figure 4, Table S3, PDB: 6Z6Y). Clear electron density for the head group structure was obtained, whereas the amide's acyl chain appeared rather mobile, so that a profiling of the head group's contacts became possible. As depicted in Figure 4A on two levels of magnification, the galactose moiety with its sulfate was central to contact building. This was expectable, when considering the analogy to cocrystals of this CRD with lactose and 3′-sialyl- and 3′-sulfolactose ligands (Ideo et al., 2011). In this position, sphingosine's hydroxyl group and side chains of Arg69/Glu89, together with a water molecule, are connected by hydrogen bonding. It hereby appears to functionally substitute the 3′-hydroxyl group of the glucose part of lactose (Figure 4B). The intriguing structural equivalence of sphingosine's hydroxyl group with the 3′-hydroxyl group of glucose of suLac, shown in Figure 4C (PDB: 3AP6), is illustrated in Figure 4D, with the water-mediated network taking the place of the direct hydrogen bonding. When using a suLac-Gal-4N structure as platform (PDB: 5DUW, Figure 4E) and arranging key contacts for galactose and sulfate accordingly, a similar structural equivalence is seen (Figure 4F).

### Conclusions

The synthesis of two forms of clickable sulfatide head group has facilitated the generation of GDSs with biomimetic model character for glycosphingolipids. This platform offers the perspectives to further modify their surface presentation by additions of other membrane constituents such as cholesterol (Róg and Vattulainen, 2014) and to build a library of test tools including typical glycans of glycoproteins. Starting with sulfated lactose, it is shown to be a docking point for human galectins. This contact enables bridging irrespective of their architecture, even in the case of wild-type Gal-3. Presentation of the sulfatide head group yielded a rather weak but significant aggregation activity for Gal-4 in the homotypic system. This interaction can physiologically underlie reported raft stabilization and superraft formation. In GDSs mixtures simulating sulfatide and glycoprotein presence, the sulfatide-1 structure supported aggregation: this can mimic recruitment of glycoprotein cargo to heterobivalent Gal-4 presented by its association to sulfatide in detergent-resistant membranes. Bringing together two different CRDs that share binding to sulfatide and have context-dependent preferences, as indicated also by galectin histochemistry with fusion proteins of the CRDs (Wasano and Hirakawa, 1999), makes this functional versatility possible. As GDSs as sensor for contact building disclose, the presence of the linker is important for accepting clustered glycan arrangements as binding site, ligand binding to Gal-4 in solution then yielding a rather compact structure favoring cargo transport (André et al., 2014; Göhler et al., 2010).

In structural terms intriguing, sphingosine's OH group can be involved in galectin-sulfatide interaction, as shown by crystallography. The importance of the illustrated physical contact of sulfatide with sugar and sphingosine is underscored by pointing to likely physiological back-ups: in the absence of sulfatide, cholesterol 3-sulfate may well play this role (Ideo et al., 2007), and our data suggest that Gal-8 is a candidate to compensate for a genetic deficiency in Gal-4 expression, this possibility discussed in principle for a Gal-3 animal model (Eude-Le Parco et al., 2009). Since homodimeric Gal-1 is not a sulfatide receptor and chimera-type Gal-3 is only converted into a receptor by an engineered change of architecture, perspectives of further strategic combinations of this type of supramolecular tool with generation of innovative protein design (lectinology 4.0 (Ludwig et al., 2019b)) are obvious.

Finally, the reported homo- and heterobivalency in the cross-linking assays explains well a role of Gal-4 in detergent-resistant membranes, and it may also underlie its specific role as suppressor for human colon cancer (Michalak et al., 2017, 2019; Rao and Rao, 2017; Rechreche et al., 1997; Satelli et al., 2011), as factor in oligodendrocyte differentiation and as inhibitor of myelination (de Jong et al., 2018, 2020; Díez-Revuelta et al., 2017; Stancic et al., 2012). Thus, the applied synthetic and supramolecular chemistry that led to detection of GDS bridging by sulfatide-Gal-4 pairing identifies a versatile means of letting sulfatide presence appear less enigmatic. This conclusion encourages further studies with the GDS platform on other sulfatide-binding proteins, i.e. adhesive glycoproteins laminin or thrombospondin (Roberts and Ginsburg, 1988), L-selectin (Honke et al., 2004; Suzuki et al., 1993), and Ig-like receptor LMIR5 (Phongsisay et al., 2015), intracellularly the HIF-1 target NOD2 (Nabatov et al., 2013), and on carbohydrate-carbohydrate interactions (Bovin, 1997; Zhao et al., 2012).

### Limitations of the study

The documented attractive possibility for versatile surface programming should not lead to take the analogy to a biorelevant design too far. Typical segregation into microdomains known from cellular membranes can not yet be mimicked. Mixtures with natural (glyco)sphingolipids and glycerophosphatides typically bearing unsaturated acyl chains and addition of cholesterol are then helpful to constitute systems with a larger number of variables that can implement and regulate fluidity. Variation of acyl chain length, for example to the C24 form of sulfatide assumed to be the preferential binding partner (Delacour et al., 2005), is likely to have an impact, too, as incorporation of such artificial glycolipids, which are obtained by the described head group synthesis and conjugation to an anchor, into liposomal or even cellular membranes can be envisioned, all with the aim to come as close to the biochemical and spatial heterogeneity of a natural membrane as possible. Equally important, the monitoring of OD_450_-value changes upon aggregation is the starting point for quantitative analysis of galectin binding including quantifying *cis*-interactions and the strength of cohesion. Nonetheless, this platform teamed up with head group tailoring and protein engineering affords a robust system to detect bridging activity of physiological relevance, as shown here by the introduction of the heterotypic aggregation assay and a human lectin actually involved in apical transport.

### Resource availability

#### Lead contact

Further information and requests for resources should be directed to and will be fulfilled by the Lead contact, Paul V. Murphy (paul.v.murphy@nuigalway.ie).

#### Materials availability

This study did not generate new unique reagents.

#### Data and code availability

The published article includes all datasets/code generated or analyzed during this study.

## Methods

All methods can be found in the accompanying Transparent Methods supplemental file.

## Supplemental information

Document S1. Transparent methods, figures S1–S5, tables S1–S3, and schemes S1–S3
