# Supplementary material for: Probing sulfatide-tissue lectin recognition with functionalized glycodendrimersomes
Source: iScience. 2020 Dec 10;24(1):101919. doi: 10.1016/j.isci.2020.101919 (PMC7773886; doi:10.1016/j.isci.2020.101919)
Supplement: Document S1. Transparent methods, figures S1–S5, tables S1–S3, and schemes S1–S3 [file mmc1.pdf]

iScience, Volume *24*

Supplemental Information

Probing sulfatide-tissue lectin recognition with functionalized glycodendrimersomes

Paul V. Murphy, Antonio Romero, Qi Xiao, Anna-Kristin Ludwig, Srinivas Jogula, Nadezhda V. Shilova, Tanuja Singh, Adele Gabba, Bilal Javed, Dapeng Zhang, Francisco J. Medrano, Herbert Kaltner, Jürgen Kopitz, Nicolai V. Bovin, Albert M. Wu, Michael L. Klein, Virgil Percec, and Hans-Joachim Gabius

Supplemental Figures and Legends

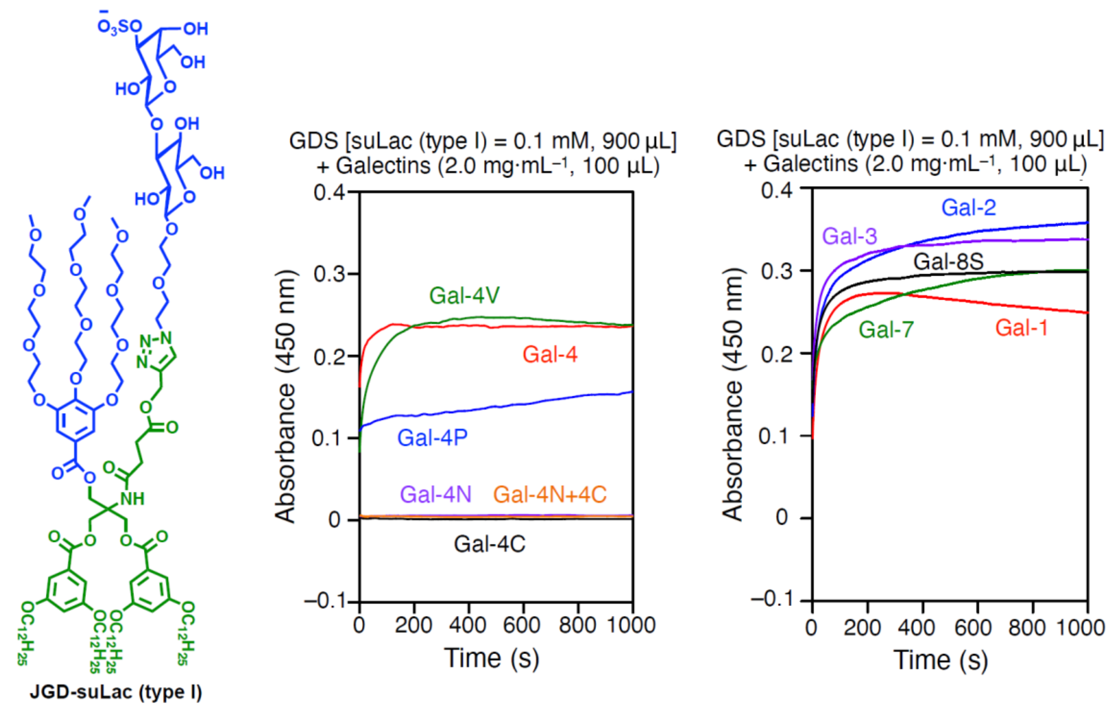

Figure S1. Course of aggregation of glycodendrimersomes (GDSs) presenting suLac (type I) with Gal-4 proteins and a panel of human galectins (2.0 mg/mL) in PBS (related to Scheme 1, Figure S3)

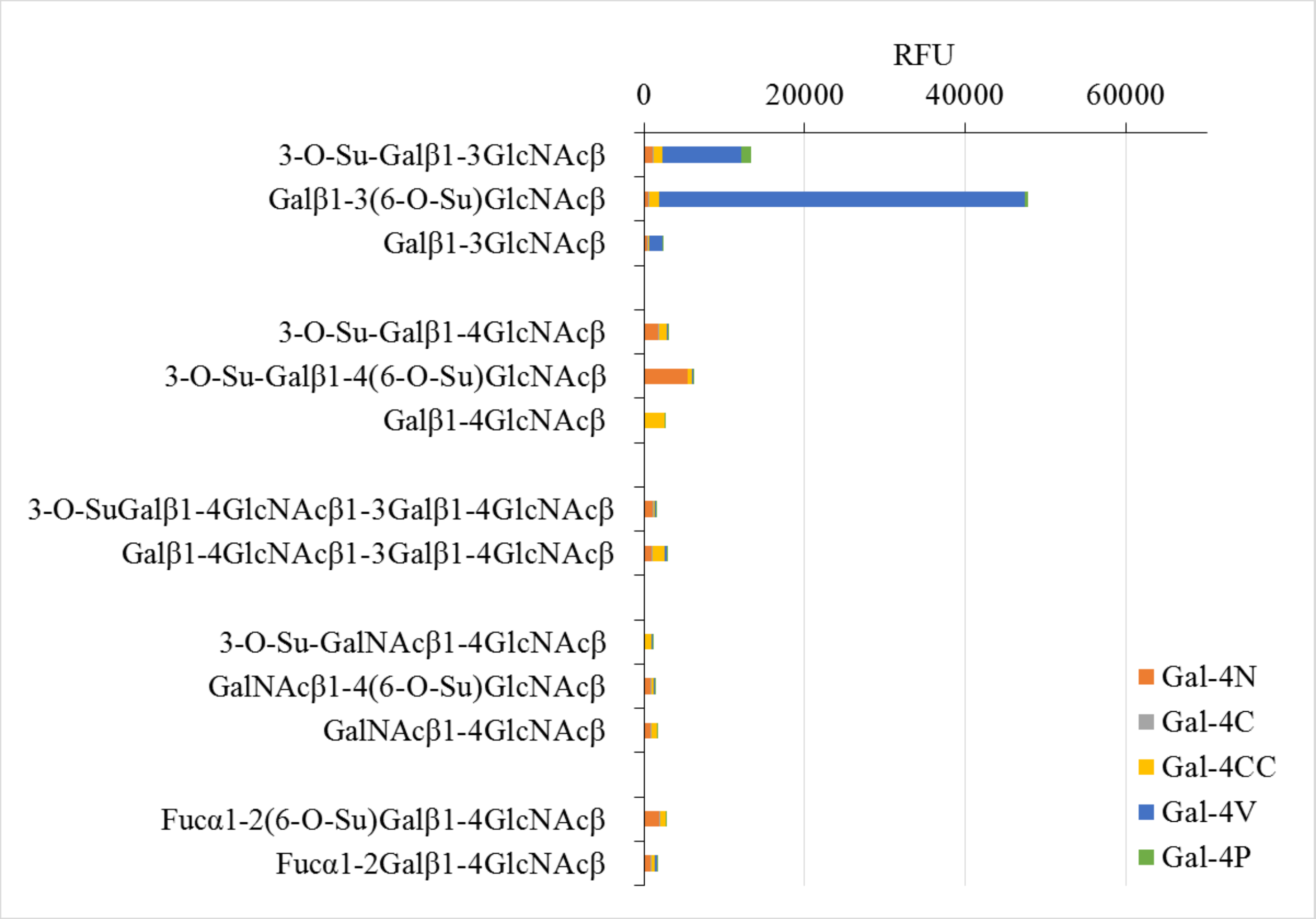

Figure S2. Stacked chart of signal intensities of binding of the two carbohydrate recognition domains (CRDs) of Gal-4, i.e. Gal-4N and Gal-4C, a Gal-4 variant constituted by two C CRDs and the variants with shortening of the 42 amino-acid-long linker to 16 amino acids (Gal-4V) and its removal (Gal-4P) to natural glycans (sulfated and parental structures) in an array (each colored part of the bar is the relative signal intensity (in relative units) for the given pair of protein and glycan). Related to Figure 1.
For details on data, see given link:
https://syncandshare.lrz.de/getlink/fiCpW4zo2HHDQfzEvRXvDu1L/Kopie_von_Gal-4_for_suppl_withoutTF.xlsx

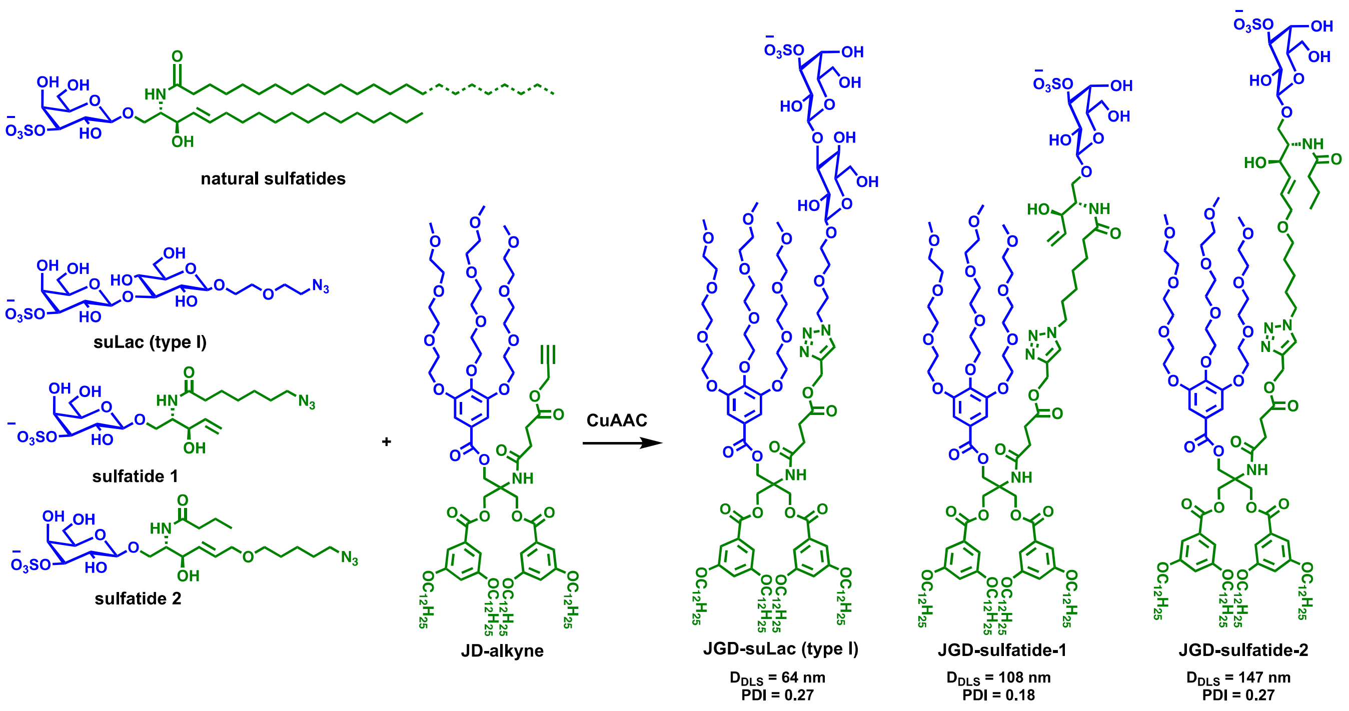

Figure S3. Synthesis of Janus glycodendrimers (JGDs) containing suLac (type I), sulfatide-1, and sulfatide-2 via copper-catalyzed azide-alkyne cycloaddition (CuAAC). Their diameter (D_{DLS}, in nm) and polydispersity (in the parentheses) were measured by dynamic light scattering (DLS) with 0.1 mM of sugar in PBS (pH = 7.4). (Related to Schemes 1-3, Figure 2, Figure S1)

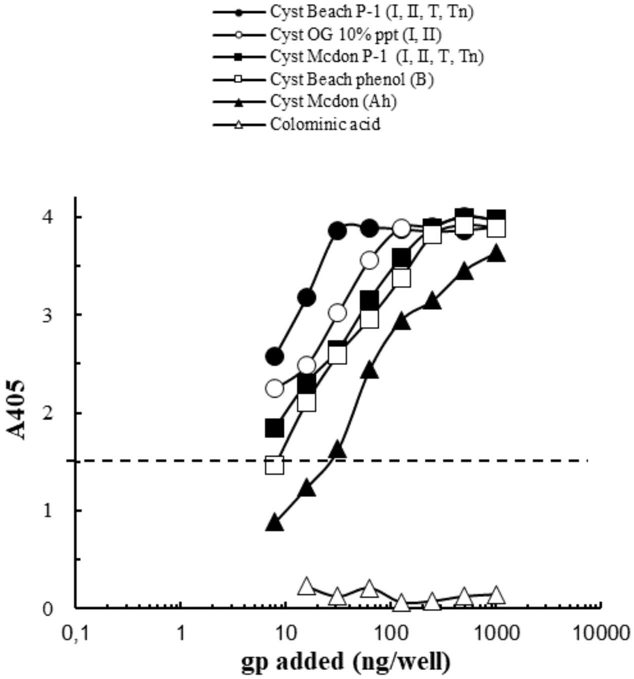

Figure S4. Binding of biotinylated Gal-4 (250 ng/well) to microtiter plates coated with six different glycoproteins. The standard deviation did not exceed 10%. Total volume of the assay was 50 μL. A_{405} was recorded after 24 h incubation. (Related to Figure 1)

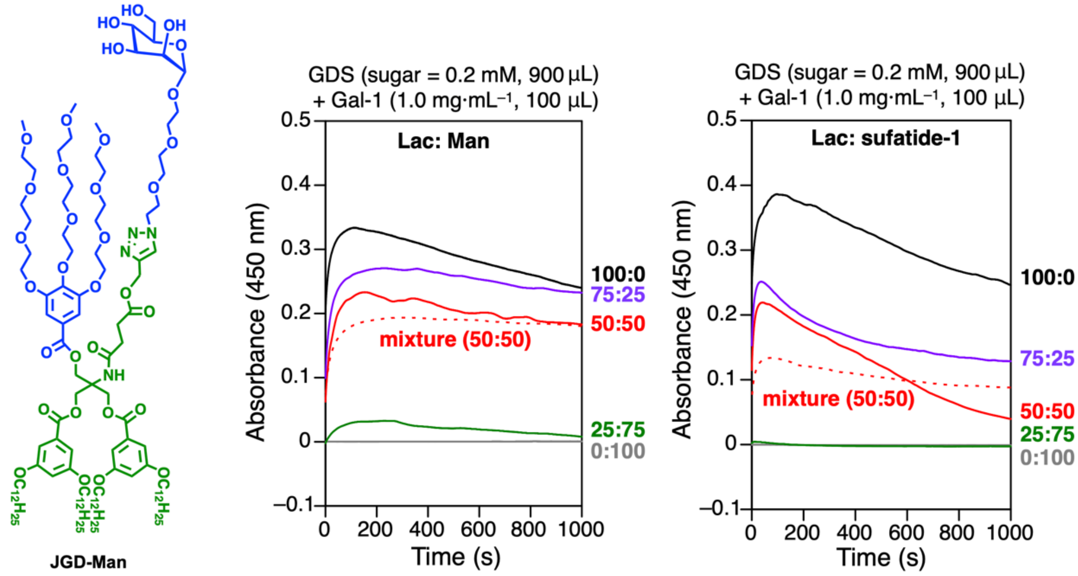

Figure S5. Course of aggregation of co-assembled glycodendrimersomes (GDSs) with Gal-1 in PBS (pH = 7.4). Related to Figure 3.

Supplemental Tables

Table S1. Reactivity of hGal-4 for natural glycoproteins (gps)[a] (Related to Figure 1)

Glycoprotein (terminal epitope)[b]	1.5 (A_{405}) unit (ng)	Maximum A_{405} absorbance	
		Absorbance reading[c]	Binding intensity[c]
Histo-blood group precursor (equivalent) gps			
Cyst Beach P-1 (**I, II, T, Tn**)	0.3	3.7	5+
Cyst OG 10% ppt (**I, II**)	1.0	3.8	5+
Cyst Mcdon P-1 (**I, II, T, Tn**)	4.0	3.7	5+
Cyst JS 1st Smith degraded (**I/II**)	4.0	3.3	5+
Hog gastric mucin #21 (**I/II**)	4.0	3.3	5+
Cyst Tighe P-1 (**I/II**)	12.0	2.9	5+
Histo-blood group ABH-active gps			
Cyst MSS 10% 2x (**A_h**)	0.7	4.0	5+
Hog gastric mucin #9 (**A_h**, **H** on core 2 and I-active O-glycans)	1.2	4.1	5+
Hog gastric mucin #4 (**A_h**, **H** on core 2 and I-active O-glycans)	2.0	3.9	5+
Cyst MSM 10% ppt (**A_h**)	4.0	3.8	5+
Cyst 19 (**B_h**)	7.0	3.6	5+
Cyst Beach phenol insoluble (**B_h**)	8.0	3.7	5+
Cyst JS phenol insoluble (**H**)	22.0	3.2	5+
Cyst Mcdon (**A_h**)	30.0	3.4	5+
Lewis[a]- and Lewis[b]- active gps			
Cyst N-1 Le^a 20% 2x (**Le^a**, **Le^x**)	15.0	3.1	5+
Asialo HOC 350 (**Le^a**)	32.0	2.8	5+
Cyst Tighe phenol insoluble (**H**, **Le^a**, **Le^b**, **Le^x**, **Le^y**)	40.0	3.4	5+
HOC 350 (sialyl **Le^a**)	-	1.1	2+
Multi-antennary Galβ1-4GlcNAc (II) gps			
Bird nest asialo gp (**II, E, T, F**)	6.0	4.1	5+
Human asialo α_1-acid gp (m**II**)	28.0	2.5	5+
Asialofetuin (m**II/I**, **T_α**)	28.0	2.3	4+
Asialo THGP Sd (a^+) W. M. (**S**, i**II**)	30.0	3.4	5+
Bovine asialo α_1-acid gp (m**II**)	30.0	2.9	5+
Porcine thyroglobulin (α2-3/6 sialyl **mII**)	40.0	2.3	4+
Bird nest gp (sialyl **II, E, T_α, F_α**)	50.0	2.0	4+
THGP Sd (a^+) W. M. (**S**, i**II**)	60.0	2.7	5+
Porcine asialothyroglobulin (m**II**)	60.0	2.3	4+
Pneumococcus type 14 polysaccharide (i**II/Lac**)	100.0	2.0	4+
Asialo RSL (m**II**)	100.0	2.3	4+
Bovine asialolactoferrin (m**II**, **B**, LacdiNAc)	150.0	3.0	5+
Fetuin (α2-3/6 sialyl m**II/I**, sialyl/disialyl **T_α**)	-	0.7	+
Human α_1-acid gp (α2-3/6 sialyl m**II**)	-	0.4	±
Human asialolactoferrin (m**II**, i**II**, **Le^x**)	-	0.2	±
RSL (sialyl m**II**)	-	0.2	±
Bovine lactoferrin (α2-6 sialyl m**II**, **B**, LacdiNAc)	-	0.0	-
Bovine α_1-acid gp (sialyl m**II**)	-	0.0	-
T, Tn-containing gps			
Asialo PSM (**Tn**, **T_α**, **A_h**, **H**)	1.9	4.0	5+
PSM (sialyl **Tn**, **T_α** **A_h**, **H**)	1.9	3.9	5+
Active antifreeze gp (**T_α**)	25.0	2.3	4+
Human asialoglycophorin (**T_α**, **Tn**, m**IIb/f**)	30.0	3.3	5+
Asialo BSM (**Tn**, GlcNAcβ1-3**Tn**, **T_α**)	50.0	2.5	5+
Asialo OSM (**Tn**, **T_α**, core 2 **II**)	80.0	2.4	4+
Human asialoagalactoglycophorin (**T_α**, **Tn**)	600.0	1.7	3+
Human glycophorin (sialyl **T_α**, **Tn**, α2-6 sialyl m**IIb/f**)	-	1.2	2+
BSM (sialyl **Tn**, GlcNAcβ1-3**Tn**, **T_α**)	-	1.0	2+

OSM (sialyl **Tn**, **T**$_{\alpha}$, core 2 **II**)	-	0.1	-

[a]Analyses were carried out by ELLSA. 250 ng of biotinylated hGal-4 was applied in solid-phase assays using various gps, ranging from 0.05 μg to 1 μg. [b]The symbol in parentheses indicates the terminal epitopes and are bolded: **I/II** (Galβ1-3/4GlcNAc); **iII/Lac** = internal Galβ1-4Glc(NAc); **A** (GalNAcα1-3Gal); **A**$_{h}$ (GalNAcα1-3[LFucα1-2]Gal); **H** (LFucα1-2Gal); **B** (Galα1-3Gal); **B**$_{h}$ (Galα1-3[LFucα1-2]Gal); **T**$_{\alpha}$ (Galβ1-3GalNAcα); **Tn** (GalNAcα1-Ser/Thr); **S** (GalNAcβ1-4Gal); **E** (Galα1-4Gal); **F** (GalNAcα1-3GalNAc); m = multi-antennary; m**IIb/f** = bi-antennary N-glycan with core fucosylation and bisecting GlcNAc. [c]The results were graded according to the spectrophotometric absorbance value at 405 nm (i.e. O.D.$_{405}$) after 24 h incubation as follows: +++++ (O.D. > 2.5), ++++ (2.5 > O.D. ≥ 2.0), +++ (2.0 > O.D. ≥1.5), ++ (1.5 > O.D. ≥ 1.0), + (1.0 > O.D. ≥ 0.5), ± (0.5 > O.D. ≥ 0.2), and - (O.D. < 0.2).

Table S2. Inhibitory potency of various glycoproteins on binding of hGal-4 (125 ng/50 μl) to a **I/II**-containing gp (Cyst beach P-1, 5 ng/50 μl)[a] (Related to Figure 1)

Inhibitor[b]	Quantity giving 50% inhibition (ng)[c]	Mass relative potency [d]
Histo-blood group precursor (equivalent) gps		
Cyst Beach P-1 (**I**, **II**, **T**, **Tn**)	0.3	7.0×10^{5}
Cyst OG 10% ppt (**I, II**)	0.9	2.3×10^{5}
Cyst Mcdon P-1 (**I, II, T, Tn**)	7.0	3.0×10^{4}
Cyst MSS 1st Smith (**I, II, Tn, T**)	7.0	3.0×10^{4}
Hog gastric mucin #14 (**I/II**)	20.0	1.0×10^{4}
Cyst JS 1st Smith degraded (**I/II**)	40.0	5.2×10^{3}
Hog gastric mucin #21 (**I/II**)	150.0	1.4×10^{3}
Cyst Tighe P-1 (**I/II**)	200.0	1.0×10^{3}
Histo-blood group ABH-active gps		
Hog gastric mucin #9 (**A**$_{h}$**, H**)	1.8	1.2×10^{5}
Hog gastric mucin #4 (**A**$_{h}$**, H**)	2.0	1.0×10^{5}
Cyst MSS 10% 2x (**A**$_{h}$)	2.0	1.0×10^{5}
Cyst Beach phenol insoluble (**B**$_{h}$)	30.0	7.0×10^{3}
Cyst 19 (**B**$_{h}$)	30.0	7.0×10^{3}
Cyst Mcdon (**A**$_{h}$)	110.0	1.9×10^{3}
Saccharides		
Tri-antennary Galβ1→4GlcNAc (Tri-**II**)	$1.0\text{x}10^{4}$	21.0
Galβ1→4Glc (**L**)	$2.7\text{x}10^{4}$	7.7
Galβ1→4GlcNAc (**II**)	$2.1\text{x}10^{5}$	1.0
Gal	4.0×10^{5}	0.5
Multi-antennary Galβ1-4GlcNAc(II) gps		
Asialo bovine α1-acid GP (m**II**)	2.0×10^{2}	1.0×10^{3}
Asialo human α1-acid (m**II**)	2.0×10^{2}	1.0×10^{3}
Asialo fetuin (**II,T**)	2.5×10^{3}	8.0×10^{1}
Pneumococcus type 14 ps (i**II**)	>555.6 (36.6%)	−
Human α1-acid (α2-3/6 sialyl m**II**)	>277.8 (4%)	−
Bovine α1-acid (sialy m**II**)	>277.8 (2%)	−
Fetuin (sialy **II,T**)	>277.8 (8%)	−
T,Tn-containing gps		
Asialo BN in H_2O (**II, E, T, F**)	3.0	7.0×10^{4}
Asialo PSM (**T, Tn, A**$_{h}$**, H**)	20.0	1.0×10^{4}
Native BN (sialyl **II, E, T**$_{\alpha}$**, F**$_{\alpha}$)	>555.6 (33.4%)	−
PSM (sialy **T, Tn**)	>1388.9 (36.7%)	−

[a]The inhibitory activity is expressed as the amount of inhibitor leading to 50% inhibition of the control lectin binding. Total volume was 50μl.
[b]The symbols in parentheses indicate the human blood group activity and/or lectin determinants. Expressed in bold are: **A** (GalNAcα1→3Gal); **A**h (GalNAcα1→3[LFuca1→2]Gal); **B**h (Galα1→3[Fucα1→3]Gal); **E** (Galα1→4Gal); **H**(LFuca1→2Gal); **T**α (Galβ1→3GalNAcα1-); **Tn**(GalNAcα1→Ser/Thr); **T**(Galβ1→3GalNAc); **I**$_{β}$/**II**$_{β}$(Galβ1→ 3/4GlcNAcβ1-); **Le**^{a}(Galβ1→3[Fucα1→4]GlcNAc); **Le**^{b}(Fucα1→2Galβ1→3[Fucα1→4]GlcNAc); m(Multivalent)
[c]The gp amount required to produce 50% inhibition of hGal4-Cyst beach P-1 glycoprotein binding.
[d]Mass Relative potency (RP) = quantity of Galβ1→4GlcNAc required for 50% inhibition is taken as 1.0/quantity of sample required for 50% inhibition.

Table S3. Data collection and refinement statistics (Related to Figure 4)

**Data collection**	
Space group	C2
Wavelength (Å)	0.979257
Cell dimensions	
a, b, c (Å)	111.2, 40.2, 40.9
α, β, γ (˚)	90, 99.9, 90
Resolution(Å)[a]	50.00 - 1.34 (1.42 - 1.34)
R_{merge}	3.3 (97.4)
CC 1/2	100 (82.3)
Completeness (%)	99.6 (98.3)
$\langle I/\sigma(I)\rangle$	21.2 (1.5)
Redundancy	6.4 (6.2)
Mol/asymmetric unit	1
**Refinement**	
Resolution (Å)	50.0 - 1.34
No. reflections	39229
R_{work} / R_{free}	17.1 / 21.3
No. atoms (non-hydrogens)	
Protein	1254
Water	121
Sulfatide	23
PEG	10
Acetate ions	24
Average B factors ($Å^2$)	
Protein atoms	31.76
Water	42.26
Sulfatide	34.19
PEG	52.14
Acetate ions	54.80
R.m.s. deviations	
Bond lengths (Å)	0.009
Bond angles (˚)	1.047
Ramachandran statistics	
Preferred (%)	98.05
Allowed (%)	1.95
Outliers (%)	0.00

[a]Values in parentheses are for highest-resolution shell.

Coordinates and structure factors have been deposited in the Protein Data Bank with accession code 6Z6Y.

Transparent Methods

Synthesis of clickable suLac(type 1), sulfatide-1 and sulfatide-2, the building blocks for JGD synthesis

Synthesis: Glycosylation between the known trichloroacetimidate **6** (Doyle et al., 2019) and diacetone-D-glucose (commercially available) gave the known β-glycosidic product **1** (60% yield) (Crich et al., 2005). After the hydrolysis of the acetonide groups and spontaneous rearrangement to the pyranoside form, the disaccharide was acetylated and then treated with 33% HBr in AcOH to give glycosyl bromide **2**. Reaction of **2** with diethylene glycol in the presence of silver carbonate in dichloromethane gave alcohol **3**. Reaction of this alcohol under Mitsunobu conditions gave the respective azide. Removal of the acyl protecting groups followed by regioselective sulfation in three steps gave **clickable suLac (type 1)**.

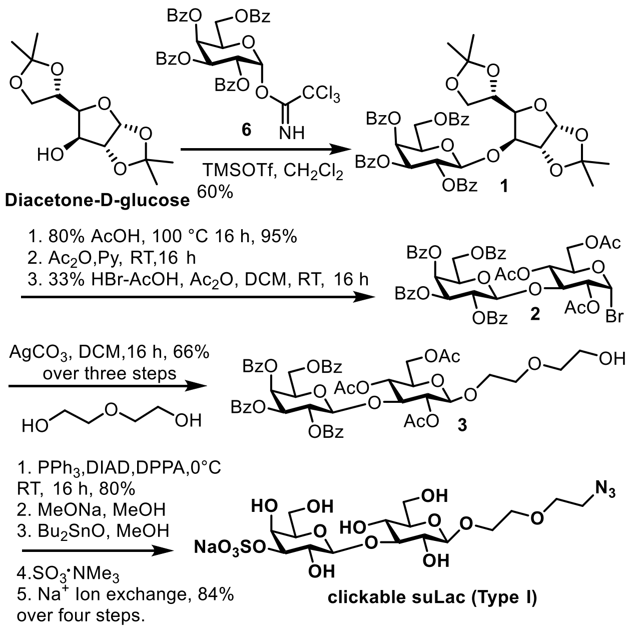

Scheme S1. Synthesis of clickable SuLac (Type-1)

The synthesis of the **clickable sulfatide 1** commenced from diol **4**, which was obtained as previously described from diacetone-D-glucose (Bundle et al.). The diol **4** was converted to the regioselectively benzoylated alcohol **5** via protection of its primary alcohol with a TBS group, then benzoylation and subsequent desilylation. Glycosidation with **6** (Doyle et al., 2019) provided **7**. Next reduction of the

azide using hydrogen sulfide (safety hazard[1]), followed by coupling with known 7-azido heptanoic acid (Wang et al., 2019) gave amide **8**. The removal of all benzoyl groups from **8** followed by regioselective sulfation gave **clickable sulfatide 1**. Yields for all steps are reported in the experimental details.

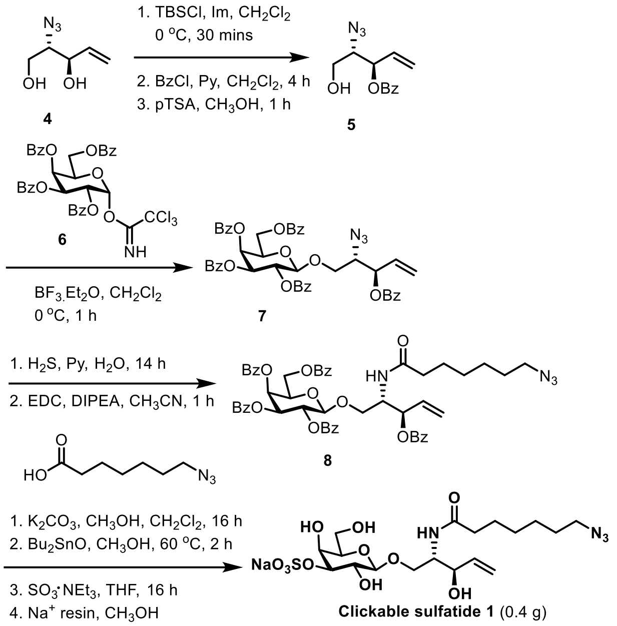

Scheme S2. Synthesis of Clickable Sulfatide-1

Preparation of **clickable sulfatide 2** was carried out from **11**, which was first prepared from D-galactal as described.[S6] The compatibility of protecting groups with certain reactions in the sequence, such as the alkylation and the requirement for orthogonal removal of PMB groups, complicated the sequence. Nevertheless, the clickable **sulfatide 2** could be prepared in ~0.4 g quantity. Thus the reduction of **11** followed by protection of the primary alcohol with a TBDPS group and subsequent Mitsunobu type substitution using azide, followed by TBDPS removal gave alcohol **12**. Alkylation was possible with TBS protected **13**, and gave **14**. We tried various methods for removal of the PMB groups, including use of DDQ, investigating a variety of conditions, but these were not successful. Use of TFA for this purpose also led to removal of TBS and so the latter was therefore exchanged for benzoate. Then successful removal of the two PMB groups was followed by reintroduction of the TBS group at the primary alcohol near the azide and the benzoate was exchanged for TBDPS. Benzoylation of the secondary alcohol followed by removal of the TBS group using aqueous HCl in THF gave acceptor **15**, which was glycosidated with **2** to give **16**. Subsequent reduction of the azide and coupling with butyric

[1] *Adequate precautions must be taken when using hydrogen sulfide as exposure to it at sufficiently high concentrations can seriously damage health or be fatal.*

acid gave **17**. Then removal of the TBDPS group followed by mesylation and substitution with azide gave **18**. Similar removal of all benzoates and sulfation as for other galactosides gave **clickable sulfatide-2**.

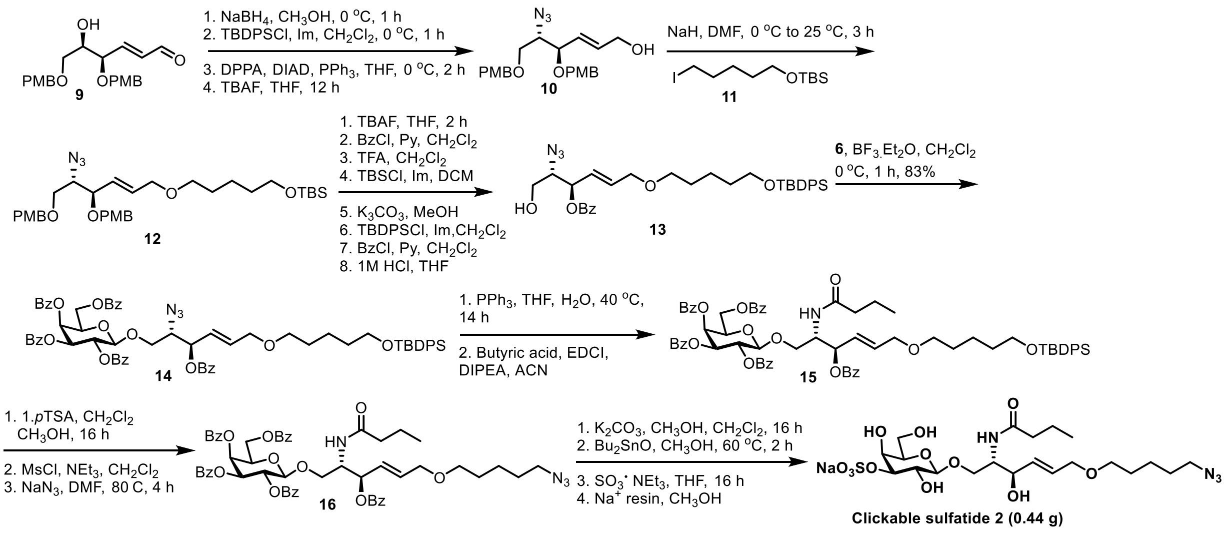

Scheme S3. Synthesis of Clickable Sulfatide-2

NMR spectra were recorded with 500 MHz Varian spectrometers. Chemical shifts are reported relative to internal Me_4Si in $CDCl_3$ (δ 0.0), HOD for D_2O (δ 4.84) or CD_2HOD (δ 3.31) for ^1H and $CDCl_3$ (77.16) or CD_3OD (49.05) for ^{13}C NMR spectra were processed and analysed using MestReNova software. ^1H-NMR signals were assigned with the aid of gCOSY. ^{13}C-NMR signals were assigned with the aid of APT, gHSQCAD and/or gHMBCAD. Coupling constants are reported in Hertz, with all J values reported uncorrected. Low- and high-resolution mass spectra were measured on a Waters LCT Premier XE Spectrometer, measuring in both positive and/or negative mode as, using MeCN, H_2O and/or MeOH as solvent. Thin layer chromatography (TLC) was performed on aluminium sheets precoated with silica gel 60 (HF254, E. Merck) and spots visualized by UV and charring with H_2SO_4-EtOH (1:20), cerium molybdate or phosphomolybdic acid staining agents. Flash chromatography was carried out with silica gel 60 (0.040-0.630 mm, E. Merck or Aldrich) and using a stepwise solvent polarity gradient (starting with the conditions indicated in each case and increasing the polarity as required), correlated with TLC mobility. Chromatography solvents, cyclohexane, EtOAc, CH_2Cl_2 and MeOH were used as obtained from suppliers (Fisher Scientific and Sigma-Aldrich). Anhydrous pyridine and DMF were purchased from Sigma Aldrich with other dried solvents (methanol, THF, dicholoromethane, toluene, diethyl ether) being used as obtained after treating with Pure Solv™ Solvent Purification System.

Detailed Synthetic Protocols

Preparation of 3 The glycoside **1** (382 mg) was dissolved in 6 mL of 80% AcOH in water and heated at 100 ºC for 16 h to yield intermediate pyranose (95%); ESI-HRMS: Calcd for $C_{40}H_{38}NaO_{15}$ $[M+Na]^+$ 781.2108; Found, 781.2147; ^{1}H NMR (500 MHz, $CDCl_3$): δ 8.07 (overlapped signals, 2H), 8.01 (overlapped signals, 3H), 7.79 (overlapped signals, 2H), 7.64 (t, J = 7.3 Hz, 1H), 7.58 – 7.49 (overlapped signals, 4H), 7.42 (overlapped signals, 5H), 7.29 – 7.22 (overlapped signals, 3H), 6.01 (s, 1H), 5.79 (t, J = 9.1 Hz, 1H), 5.62 (d, J = 12.8 Hz, 1H), 5.53 (s, 1H), 4.99 (d, J = 7.9 Hz, 1H), 4.57 (d, J = 5.9 Hz, 2H), 4.44 – 4.35 (overlapped signals, 2H), 4.23 (s, 1H), 4.20 – 4.15 (m, 1H), 4.15 – 4.09 (m, 1H), 3.88 (d, J = 11.7 Hz, 1H), 3.70 (dd, J = 11.5, 5.8 Hz, 1H); ^{13}C NMR (125 MHz, $CDCl_3$) δ 165.5, 165.4, 164.8, 163.2, 133.8, 133.7, 133.4, 133.4, 130.0, 129.9, 129.8, 129.6, 129.1, 128.9, 128.8, 128.7, 128.6, 128.5, 128.5, 128.3, 112.2, 105.2, 101.9, 83.6, 83.2, 79.9, 72.3, 71.3, 69.5, 68.8, 68.0, 64.3, 62.2. To this intermediate (1.25 g, 1.49 mmol) in pyridine (7 mL), Ac_2O (7 mL) was slowly added at 0 °C. The reaction mixture was stirred for 16 h at room temperature. The solvents were then removed by co-evaporation with toluene and the residue dissolved in dry dichloromethane (5.5 mL). The mixture was cooled to 0 °C, then 33% HBr in AcOH (1.85 mL) and Ac_2O (5mL) were added and the mixture left for 2 h at room temperature. The mixture was diluted with 10 mL dichloromethane and cold sat aq $NaHCO_3$ was added. The aqueous phase was extracted with dichloromethane (20 mL x 3). The combined organic phases were dried over Na_2SO_4 and the solvent removed under reduced pressure to give the residue which was the glycosyl bromide intermediate **2**. The bromide obtained was dissolved in dry dichloromethane (13 mL), then diethyleneglycol (DEG, 2.6 mL, 27.5 mmol) and silver carbonate (640 mg, 2.33 mmol) were added. The mixture was stirred at 30 °C for 16 h in the dark. The mixture was passed through celite®, washing with water and dichloromethane. The phases were separated and the organic portion dried over Na_2SO_4 and the solvent removed under reduced pressure. Column chromatography (1:1 cyclohexane-EtOAc) gave **3** (955 mg, 66%); ESI-HRMS: Calcd for $C_{50}H_{52}O_{20}Na$, $[M+Na]^+$, 995.2950; Found, 995.2957; ^{1}H NMR (500 MHz, $CDCl_3$): δ 8.06 (overlapped signals, 4H), 7.85 (overlapped signals, 2H), 7.74 (overlapped signals, 2H), 7.63 (t, J = 7.5 Hz, 1H), 7.58 (t, J = 7.4 Hz, 1H), 7.52 (overlapped signals, 2H), 7.46 (overlapped signals, 3H), 7.41 (t, J = 7.4 Hz, 1H), 7.35 (overlapped signals, 2H), 7.21 (overlapped signals, 2H), 5.95 (d, J = 3.0 Hz, 1H), 5.67 – 5.57 (overlapped signals, 2H), 5.10 (t, J = 9.5 Hz, 1H), 4.95 (overlapped signals, 2H), 4.67 (dd, J = 10.8, 6.2

Hz, 1H), 4.42 (d, J = 7.8 Hz, 1H), 4.35 (dd, J = 10.8, 7.0 Hz, 1H), 4.30 (t, J = 6.6 Hz, 1H), 4.21 (dd, J = 12.2, 4.7 Hz, 1H), 4.15 (dd, J = 12.4, 2.7 Hz, 1H), 4.14 – 4.09 (m, 1H), 3.99 (t, J = 9.2 Hz, 1H), 3.66 (overlapped signals, 2H), 3.61 (dd, J = 11.0, 5.0 Hz, 1H), 3.58 – 3.54 (overlapped signals, 3H), 3.51 (overlapped signals, 2H), 2.09 (s, 3H), 2.07 (s, 3H), 1.93 (s, 3H);^{13}C NMR (125 MHz, $CDCl_3$)δ 170.8, 169.1, 168.6, 166.0, 165.6, 165.4, 165.1, 133.7, 133.4, 133.3, 133.1, 129.9, 129.8, 129.7, 129.6, 129.4, 129.3, 128.9, 128.8, 128.5, 128.5, 128.3, 128.2, 101.1, 100.7, 78.3, 77.3, 77.0, 76.8, 72.5, 72.3, 71.7, 71.0, 70.1, 69.9, 68.3, 68.3, 67.8, 62.2, 61.6, 61.6, 20.9, 20.8, 20.7.

Preparation of clickable suLac (type 1) To **3** (888 mg, 0.91 mmol) in THF (10 mL) at 0 ºC, PPh_3 (765 mg, 2.9 mmol) and DIAD (590 mg, 2.9 mmol) were added. The reaction mixture was stirred at 0 ºC until the solution became clear. DPPA (826 mg, 3.0 mmol, 3.3 eq.) was then added and the reaction was allowed to attain room temperature and stirred for 2 h. The mixture was diluted with EtOAc (100 mL) and washed with H_2O (3 x 15 mL). The organic portion was dried over Na_2SO_4 and the solvent removed under reduced pressure. Column chromatography (7:3 to 1:1 cyclohexane-EtOAc, gradient elution) gave the intermediate azide (725 mg, 80%); ESI-HRMS: Calcd for $C_{52}H_{50}N_3O_{19}$, $[M+H]^+$, 1020.3039; Found, 1020.3043; ^{1}H NMR (500 MHz, $CDCl_3$) δ 8.10–8.03 (overlapped signals, 4H), 7.88–7.83 (overlapped signals, 2H), 7.74 (overlapped signals, 2H), 7.64 (t, J = 7.0 Hz, 1H), 7.59 (t, J = 7.5 Hz, 1H), 7.52 (overlapped signals, 2H), 7.46 (overlapped signals, 3H), 7.41 (t, J = 7.4 Hz, 1H), 7.35 (t, J = 7.8 Hz, 2H), 7.22 (overlapped signals, 2H), 5.95 (dd, J = 3.2, 1.1 Hz, 1H), 5.68 – 5.61 (m, 1H), 5.61 – 5.56 (m, 1H), 5.10 (t, J = 9.5 Hz, 1H), 4.95 (overlapped signals, 2H), 4.66 (dd, J = 11.0, 6.4 Hz, 1H), 4.43 (dd, J = 7.9, 1.0 Hz, 1H), 4.38 – 4.34 (m, 1H), 4.34 – 4.26 (m, 1H), 4.21 (dd, J = 12.2, 4.7 Hz, 1H), 4.15 (dd, J = 12.3, 2.5 Hz, 1H), 3.98 (t, J = 9.3 Hz, 1H), 3.85 (dt, J = 11.1, 3.8 Hz, 1H), 3.69 – 3.61 (overlapped signals, 2H), 3.61 – 3.55 (overlapped signals, 3H), 2.09 (s, 3H), 2.07 (s, 3H), 1.92 (s, 3H); ^{13}C NMR (125 MHz, $CDCl_3$): δ 169.0, 168.4, 166.0, 165.4, 165.1, 156.3, 155.8, 133.7, 133.4, 133.3, 133.1, 129.9, 129.8, 129.7 (2 signals), 129.5, 129.3, 128.9, 128.8, 128.5, 128.5, 128.3, 128.1, 101.1, 100.8, 78.3, 72.4, 72.0, 71.0, 70.4, 70.2, 70.1, 68.5, 68.3, 67.8, 62.2, 61.6, 50.8, 20.9, 20.8, 20.7. To the azide (700 mg, 0.7 mmol) in dry dichloromethane (7 mL), freshly prepared sodium methoxide in dry methanol was added until the pH was 10 and the solution was stirred at room temperature for 4 h. The solution was neutralized with an ion exchange resin (Dowex 50 × 8, H^+ form), filtered and concentrated to give the fully deacylated intermediate in quantitative yield. This intermediate and Bu_2SnO (227 mg, 0.912 mmol) were stirred in dry MeOH (30 mL) while heating at reflux under argon for 2 h. The solvent

was removed under reduced pressure and the resulting complex was treated with $Me_3N \cdot SO_3$ (166 mg, 1.19 mmol) in dry THF (30 mL) at room temperature for 12 h. The solvent was evaporated off under reduced pressure, then the residue was dissolved in 1:1 $CHCl_3$-MeOH, and passed through a cation exchange resin column (Dowex 50 × 8 Na^+ form), eluting with 1:1 $CHCl_3$-MeOH. The solvents were then removed under reduced pressure and flash chromatography (8:2 to 7:3, $CHCl_3$-MeOH) gave the **clickable suLac (type 1)** (312 mg, 84%); ESI-HRMS: Calcd for $C_{16}H_{28}N_3O_{15}S$, $[M-Na]^-$, 534.1241; Found, 534.1245; ^{1}H NMR (500 MHz, CD_3OD) δ 4.63 (d, J = 7.8 Hz, 1H, H-1), 4.38 (d, J = 7.9 Hz, 1H, H-1), 4.26 (dd, J = 9.7, 3.2 Hz, 1H, galactose H-3), 4.23 (d, J = 2.6 Hz, 1H)., 4.01 (dt, J = 10.4, 4.4 Hz, 1H), 3.87 (d, J = 11.5 Hz, 1H), 3.82 – 3.74 (overlapped signals, 3H), 3.74 – 3.64 (overlapped signals, 6H), 3.65 – 3.55 (overlapped signals, 2H), 3.47 – 3.37 (overlapped signals, 4H), 3.35 – 3.32 (m, 1H); ^{13}C NMR (125 MHz, MeOD) δ 105.3, 103.7, 87.7, 81.3, 77.4, 76.6, 74.3, 71.2, 71.2, 70.8, 69.9, 69.6, 68.5, 62.5, 62.4, 51.7.

Preparation of 5 To stirred **4** (1 g, 6.9 mmol) in 25 mL dry dichloromethane at 0 ^{o}C under nitrogen, was added imidazole (0.71 g, 10.5 mmol) followed by TBSCl (1.16 g, 7.6 mmol). The mixture was stirred for 30 mins at 0 ^{o}C and then 10 mL H_2O was added and the mixture extracted with dichloromethane (2 x 10 mL). The organic portions were combined and dried over Na_2SO_4, and the solvent was removed at 30 ^{o}C, using a rotary evaporator (150 mm Hg). The residue obtained was used in the next step, without further purification. A small portion was subjected to flash column chromatography with 15% EtOAc–cyclohexane as eluant to give sample for analysis; TLC: R_f 0.3 in 1:4 EtOAc-cyclohexane; HRMS: $[M+Cl]^-$ Calcd for $C_{11}H_{23}N_3O_2SiCl$ 292.1248, found 292.1252; ^{1}H NMR (500 MHz, $CDCl_3$): δ 5.97 – 5.84 (m, 1H), 5.39 (dp, *J* = 17.2, 1.4 Hz, 1H), 5.29 (dp, *J* = 10.6, 1.4 Hz, 1H), 4.29 (q, *J* = 5.6 Hz, 1H), 3.91 – 3.78 (overlapped signals, 2H), 3.43 (td, *J* = 5.7, 3.0 Hz, 1H), 2.50 (dd, *J* = 5.6, 1.7 Hz, 1H), 0.94 – 0.88 (overlapped signals, 9H), 0.10 (d, *J* = 1.8 Hz, 6H); ^{13}C NMR (125 MHz, $CDCl_3$): δ 136.5, 117.5, 73.3, 65.9, 63.5, 25.7, 18.1, -5.6. To the residue, taken up in 15 mL dry dichloromethane, at 0 ^{o}C under nitrogen, was added 3 mL pyridine followed by BzCl (1.2 mL, 10.5 mmol). The mixture was stirred for 4 h at room temperature and then sat aq $NaHCO_3$ solution was added at 0° C, and extracted with dichloromethane (2 x 15 mL). The combined organic portions dried over Na_2SO_4 and the solvent was removed under reduced pressure. The residue was used in the next step without further purification. A small portion was subjected to flash column chromatography (1:19 EtOAc–cyclohexane) to give a sample for analysis; TLC: R_f 0.2 in 1:19 EtOAc–cyclohexane; HRMS:

$[2M+H]^+$ Calcd for $C_{36}H_{55}N_6O_6Si_2$ 723.3722, found 723.3713; ^{1}H NMR (500 MHz, $CDCl_3$) δ 8.07 (overlapped signals, 2H), 7.59 (td, *J* = 7.4, 1.4 Hz, 1H), 7.46 (overlapped signals, 2H), 5.96 (dddd, *J* = 17.2, 10.5, 6.9, 1.3 Hz, 1H), 5.66 (ddt, *J* = 6.9, 4.6, 1.1 Hz, 1H), 5.46 (dq, *J* = 17.3, 1.1 Hz, 1H), 5.38 (dt, *J* = 10.5, 1.1 Hz, 1H), 3.89 – 3.67 (overlapped signals, 3H), 0.91 (overlapped signals, 10H), 0.08 (overlapped signals, *J* = 1.7 Hz, 6H).

^{13}C NMR (125 MHz, $CDCl_3$) δ 165.2, 133.3, 131.8, 129.7, 128.5, 120.2, 74.2, 65.5, 62.7, 25.7, 18.2, -5.5, -5.6. To this intermediate in 10 mL of MeOH, *p*TSA (0.13 g, 0.7 mmol) was added. After stirring for 1 h at room temperature, 5 mL sat aq $NaHCO_3$ solution was added and the mixture was extracted with dichloromethane (2 x 15 mL), the combined organic portions were dried over Na_2SO_4 and the solvent was removed under reduced pressure. Flash column chromatography, with 20% EtOAc–cyclohexane as eluant, gave **5** (1.2 g, 72% over 3 steps); TLC: R_f 0.2 in 1:4 EtOAc–cyclohexane; HRMS: $[M+Cl]^-$ Calcd for $C_{12}H_{13}N_3O_3Cl$ 282.0645, found 282.0652; ^{1}H NMR (500 MHz, $CDCl_3$) δ 8.10 – 8.04 (overlapped signals, 2H), 7.63 – 7.56 (m, 1H), 7.47 (overlapped signals, 2H), 6.00 (ddd, *J* = 17.3, 10.5, 6.9 Hz, 1H), 5.68 (ddt, *J* = 7.1, 5.0, 1.1 Hz, 1H), 5.50 (dt, *J* = 17.2, 1.2 Hz, 1H), 5.41 (dt, *J* = 10.5, 1.1 Hz, 1H), 3.88 – 3.77 (overlapped signals, 2H), 3.69 (dd, *J* = 11.7, 7.1 Hz, 1H), 2.08 (s, 1H); ^{13}C NMR (125 MHz, $CDCl_3$) δ 165.4, 133.5, 131.8, 129.8, 129.5, 128.6, 120.5, 74.3, 65.8, 61.8.

Preparation of 7 To stirred **6** (4.0 g, 5.4 mmol), in 35 mL of dry dichloromethane at 0 °C under nitrogen, $BF_3.Et_2O$ (0.7 mL, 5.4 mmol) was charged slowly. The mixture was stirred for 30 mins at 0 °C. Then compound **5** (0.9 g, 3.6 mmol) in 5 mL of dry dichloromethane was charged slowly and the mixture was stirred for 30 mins at 0 °C. Then 15 mL sat aq $NaHCO_3$ solution was added and the mixture was extracted with dichloromethane (2 x 30 mL), then the organic portions were combined and dried over Na_2SO_4, and the solvent was removed under reduced pressure. Flash column chromatography, using 14:86 EtOAc–cyclohexane as eluant, gave **7** (2.4 g, 81%) as a white solid; TLC: R_f 0.2 in 15:85 EtOAc–cyclohexane; HRMS $[M+Na]^+$ Calcd for $C_{46}H_{39}N_3O_{12}Na$ 848.2431, found 848.2405; ^{1}H NMR (500 MHz, $CDCl_3$) δ 8.11 (overlapped signals, 2H), 8.06 – 7.94 (overlapped signals, 6H), 7.82 – 7.76 (overlapped signals, 2H), 7.66 – 7.60 (m, 1H), 7.58 – 7.53 (overlapped signals, 2H), 7.53 – 7.47 (overlapped signals, 3H), 7.43 (overlapped signals, 5H), 7.37 (overlapped signals, 2H), 7.27 – 7.24 (overlapped signals, 2H), 5.99 (dd, *J* = 3.4, 1.2 Hz, 1H), 5.92 – 5.80 (overlapped signals, 2H), 5.69 – 5.64 (m, 1H), 5.62 (dd, *J* = 10.4, 3.5 Hz, 1H), 5.32 (dt, *J* = 17.2, 1.2 Hz, 1H), 5.25 (dt, *J* = 10.6, 1.1 Hz, 1H), 4.90 (d, *J* = 7.9 Hz, 1H), 4.63 (dd, *J* = 11.1, 6.3 Hz, 1H), 4.38 (dd, *J* = 11.1, 6.6 Hz, 1H),

4.36 – 4.31 (m, 1H), 4.09 (dd, J = 10.2, 6.8 Hz, 1H), 4.01 (ddd, J = 6.7, 5.4, 4.2 Hz, 1H), 3.78 (dd, J = 10.2, 5.4 Hz, 1H); ^{13}C NMR (125 MHz, $CDCl_3$) δ 166.0, 165.6, 165.6, 165.1, 165.0, 163.6, 133.7, 133.3, 133.3, 133.3, 131.3, 130.1, 129.8, 129.8, 129.8, 129.7, 129.6, 129.3, 129.2, 128.9, 128.7, 128.7, 128.5, 128.5, 128.4, 128.4, 128.4, 128.3, 128.3, 120.5, 101.2, 74.5, 71.6, 71.5, 69.6, 8.0, 67.9, 63.1, 61.9.

Preparation of 8 H_2S gas (generated from Na_2S with dilute H_2SO_4) was bubbled, for 30 mins, to stirred **7** (2 g, 2.4 mmol) in 10 mL of pyridine and 10 mL of H_2O. The mixture was stirred for 14 h at room temperature and then bubbled with N_2 gas to remove the unreacted H_2S gas. It was then diluted with 20 mL of H_2O and extracted with dichloromethane (2 x 25 mL). The combined organic portions were dried over Na_2SO_4, then the solvent was removed under reduced pressure, to give the amine intermediate, which was used in the next step without further purification. This amine was dissolved in 15 mL of MeCN, and 1 mL of DIPEA was added as well as 7-azidoheptanoic acid (0.45 g, 2.6 mmol), followed by EDC (0.65 mL, 3.6 mmol). The mixture was stirred at room temperature for 1 h and then diluted with 10 mL sat aq $NaHCO_3$ and extracted with EtOAc (2x30 mL). The combined organic portions were dried over Na_2SO_4, and the solvent was removed under reduced pressure. Flash column chromatography with 1:3 EtOAc–cyclohexane, gave **8** (1.26 g, 56% over 2 steps) as a white solid; TLC: R_f 0.6 in 1:1 EtOAc–cyclohexane; HRMS $[M+Na]^+$ Calcd for $C_{53}H_{52}N_4O_{13}Na$ 975.3429, found 974.3424; ^{1}H NMR (500 MHz, $CDCl_3$) δ 8.14 – 8.09 (overlapped signals, 2H), 8.08 – 8.02 (overlapped signals, 2H), 7.99 – 7.92 (overlapped signals, 4H), 7.77 (overlapped signals, 2H), 7.68 – 7.61 (m, 1H), 7.52 (overlapped signals, 5H), 7.47 – 7.33 (overlapped signals, 7H), 7.29 – 7.24 (overlapped signals, 2H), 5.92 (overlapped signals, 2H), 5.81 (d, J = 9.2 Hz, 1H), 5.74 (ddd, J = 10.3, 7.7, 2.1 Hz, 1H), 5.64 (overlapped signals, 2H), 5.43 – 5.36 (m, 1H), 5.26 (d, J = 10.7 Hz, 1H), 4.80 (dd, J = 7.7, 2.1 Hz, 1H), 4.53 (ddt, J = 9.7, 6.4, 3.2 Hz, 1H), 4.38 (ddd, J = 10.8, 5.9, 2.1 Hz, 1H), 4.31 – 4.17 (overlapped signals, 3H), 3.72 (dt, J = 10.0, 2.9 Hz, 1H), 3.20 (overlapped signals, 2H), 1.84 (overlapped signals, 2H), 1.55 – 1.39 (overlapped signals, 4H), 1.30 – 1.21 (overlapped signals, 2H), 1.15 (overlapped signals, 2H); ^{13}C NMR (125 MHz, $CDCl_3$) δ 172.5, 165.9, 165.5, 165.5, 165.1, 133.7, 133.6, 133.4, 133.3, 133.3, 133.1, 130.1, 130.0, 129.8, 129.7, 129.6, 129.3, 129.1, 129.0, 128.7, 128.6, 128.4, 128.3, 119.0, 100.9, 74.1, 71.3, 71.2, 70.2, 67.9, 67.1, 61.7, 51.3, 50.3, 36.2, 28.6, 28.5, 26.4, 25.2.

Preparation of clickable sulfatide-1 To stirred **8** (1.1 g, 1.1 mmol), in 10 mL of 1:1 MeOH–dichloromethane, was added K_3CO_3 (0.16 g, 1.1 mmol). The reaction mixture was stirred for 16 h at room temperature. The solvent was removed under reduced pressure and flash chromatography with

18:82 MeOH–dichloromethane, gave intermediate tetra-ol (0.4 g, 86%) as a white solid; TLC: R_f 0.2 in 15:85 MeOH–dichloromethane; HRMS $[M+Na]^+$ Calcd for $C_{18}H_{32}N_4O_8Na$ 455.2118, found 455.2124; ^1H NMR (500 MHz, CD_3OD) δ 7.89 (d, *J* = 9.1 Hz, 1H), 5.87 (dddd, *J* = 17.1, 10.5, 6.7, 1.0 Hz, 1H), 5.27 (dt, *J* = 17.2, 1.5 Hz, 1H), 5.14 (dq, *J* = 10.4, 1.3 Hz, 1H), 4.22 (dd, *J* = 7.5, 1.0 Hz, 1H), 4.19 – 4.12 (overlapped signals, 2H), 4.02 (dtt, *J* = 10.2, 5.0, 3.2 Hz, 1H), 3.82 (d, *J* = 3.3 Hz, 1H), 3.76 (dd, *J* = 11.3, 7.0 Hz, 1H), 3.71 (dd, *J* = 11.3, 5.1 Hz, 1H), 3.61 (dd, *J* = 10.3, 3.5 Hz, 1H), 3.56 – 3.49 (overlapped signals, 2H), 3.47 (dd, *J* = 9.8, 3.3 Hz, 1H), 3.27 (overlapped signals 2H), 2.20 (overlapped signals, 2H), 1.59 (overlapped signals, 4H), 1.37 (overlapped signals, 4H); ^{13}C NMR (125 MHz, CD_3OD) δ 174.7, 138.2, 115.5, 103.9, 75.4, 73.4, 71.8, 71.2, 68.9, 68.3, 61.1, 53.3, 51.0, 35.6, 28.4, 28.3, 26.1, 25.4; To this tetra-ol (0.3 g, 0.7 mmol) in 15 mL dry MeOH, was added Bu_2SnO (0.26 g, 1.0 mmol). The reaction mixture was heated at 60° C for 2 h. Then the solvent was removed under reduced pressure, and this was followed by drying under high vacuum for 30 mins. To well dried residue, which had been taken up in 15 mL dry THF, was added $SO_3.NEt_3$ (0.19 g, 1.4 mmol). The reaction mixture was stirred for 16 h at room temperature and then the solvent was removed under reduced pressure and the residue dissolved in 20 mL of 1:1 MeOH–dichloromethane and passed through a small bed of Na^+ resin, to form the sodium salt. The solvent was removed under reduced pressure and flash column chromatography with 20% MeOH–dichloromethane, gave **clickable sulfatide-1** (0.33 g, 91%); TLC: R_f 0.2 in 20% MeOH–dichloromethane; HRMS $[M+H]^+$ Calcd for $C_{18}H_{32}N_4O_{11}NaS$ 535.1686, found 535.1689; ^1H NMR (500 MHz, CD_3OD) δ 5.86 (dddd, *J* = 17.2, 10.4, 6.7, 1.8 Hz, 1H), 5.27 (dq, *J* = 17.3, 1.7 Hz, 1H), 5.13 (dq, *J* = 10.4, 1.6 Hz, 1H), 4.33 (dd, *J* = 7.8, 1.8 Hz, 1H), 4.27 – 4.14 (overlapped signals, 4H), 3.99 (dq, *J* = 8.2, 3.2, 2.6 Hz, 1H), 3.73 (overlapped signals, 3H), 3.63 – 3.54 (overlapped signals, 2H), 3.28 (overlapped signals, 2H), 2.20 (overlapped signals, 2H), 1.66 – 1.55 (overlapped signals, 4H), 1.45 – 1.30 (overlapped signals, 4H); ^{13}C NMR (125 MHz, CD_3OD) δ 174.63, 138.2, 115.5, 103.7, 80.4, 75.0, 71.7, 69.6, 68.3, 67.1, 61.0, 53.2, 51.0, 35.6, 28.4, 28.3, 26.1, 25.4.

Preparation of 10 To aldehyde **9** (14 g, 36 mmol) in 1:10 MeOH–THF (55 mL) at 0 °C was added, in a portionwise manner, $NaBH_4$ (1.51 g, 39.9 mmol). The mixture was stirred at room temperature for 1 h and then 30 mL of sat NH_4Cl solution was added at 0 °C, and the resulting mixture then extracted with EtOAc (2 x 150 mL). The organic layers were combined and washed with 60 mL of sat brine solution and the organic layer dried over Na_2SO_4. Flash column chromatography with 3:2 EtOAc–cyclohexane, gave intermediate allylic alcohol (11.4 g, 81%) as a colourless oil; TLC: R_f 0.1 in 1:1 EtOAc–cyclohexane; HRMS: $[M+Na]^+$ Calcd for $C_{22}H_{28}O_6Na$ 411.1784, found 411.1776; ^1H NMR (500 MHz,

$CDCl_3$): δ 7.22 (overlapped signals, 4H), 6.86 (overlapped signals, 4H), 5.87 (dt, *J* = 15.6, 5.2Hz, 1H), 5.64 (ddt, *J* = 15.7, 7.8, 1.6 Hz, 1H), 4.55 (d, *J* = 11.2 Hz, 1H), 4.47 (d, *J* = 11.6 Hz, 1H), 4.41 (d, *J* = 11.6 Hz, 1H), 4.28 (d, *J* = 11.3 Hz, 1H), 4.13 (overlapped signals, 2H), 3.92 (dd, *J* = 7.8, 6.1 Hz, 1H), 3.79 (overlapped signals, 6H), 3.73 (m, 1H), 3.52 (dd, *J* = 10.0, 4.1 Hz, 1H), 3.44 (dd, *J* = 10.0, 5.53 Hz, 1H), 2.78 (br s, 1H), 1.81 (br s, 1H); ^{13}C NMR (125 MHz, $CDCl_3$): δ 159.3, 159.3, 134.3, 130.1, 130.1, 129.5, 129.5, 127.8, 113.8, 113.8, 79.4, 73.0, 73.0, 70.3, 70.1, 62.7, 55.3. To this diol (10 g, 26 mmol) in 60 mL of dry CH_2Cl_2 at 0 ^{o}C under N_2 were added imidazole (2.6 g, 39 mmol) and TBDPSCl (7.4 mL, 28 mmol) at the same temperature. The reaction mixture was stirred at room temperature for 1 h, then diluted with 40 mL of water and extracted with CH_2Cl_2 (2 x 60 mL). The combined organic portions were dried over Na_2SO_4 and the solvent removed under reduced pressure. Flash column chromatography with 1:9 EtOAc–cyclohexane, gave the silylated intermediate (15.2 g, 94%) as a colourless liquid; TLC: R_f 0.2 in 1:4 EtOAc–cyclohexane; HRMS: $[M+Na]^+$ Calcd for $C_{38}H_{46}O_6SiNa$ 649.2961, found 649.2938; ^{1}H NMR (500 MHz, $CDCl_3$): δ 7.71 – 7.65 (overlapped signals, 4H), 7.46 – 7.39 (overlapped signals, 2H), 7.37 (overlapped signals, 4H), 7.22 (overlapped signals, 4H), 6.85 (overlapped signals, 4H), 5.82 (dt, *J* = 15.5, 4.4 Hz, 1H), 5.69 (dd, *J* = 15.6, 7.9 Hz, 1H), 4.54 (d, *J* = 11.2 Hz, 1H), 4.47 (d, *J* = 11.7 Hz, 1H), 4.40 (d, *J* = 11.7 Hz, 1H), 4.29 – 4.20 (overlapped signals, 2H), 3.91 (t, *J* = 7.3 Hz, 1H), 3.80 (s, 3H), 3.77 (s, 3H), 3.74 – 3.68 (m, 1H), 3.53 (dd, *J* = 10.1, 3.5 Hz, 1H), 3.41 (dd, *J* = 10.1, 5.4 Hz, 1H), 2.68 (d, *J* = 3.5 Hz, 1H), 1.07 (s, 9H); ^{13}C NMR (125 MHz, $CDCl_3$): δ 159.2, 159.2, 135.5, 134.6, 133.6, 133.6, 130.2, 129.7, 129.6, 129.4, 127.7, 126.4, 113.8, 113.7, 79.7, 73.1, 70.2, 70.1, 63.7, 55.3, 55.2, 26.8, 19.3; To this intermediate (12 g, 19 mmol) in 100 mL of dry THF at 0 ^{o}C under nitrogen were added PPh_3 (10.0 g, 38.3 mmol) and DIAD (7.5 mL, 38.3 mmol) and the mixture was stirred for 30 mins at 0 ^{o}C, and then DPPA (8.3 mL, 38.3 mmol) was added. The reaction mixture was stirred for 1 hour. Then reaction mixture was diluted with 50 mL water and extracted with dichloromethane (2 x 80 mL), the organic portions were combined and washed with 40 mL of sat brine solution, dried over Na_2SO_4 and the solvent was removed under reduced pressure. Flash column chromatography with 1:19 EtOAc–cyclohexane, gave the intermediate azide (10.7 g, 86%) as a colourless liquid; TLC: R_f 0.6 in 1:4 EtOAc–cyclohexane; HRMS: $[M+Na]^+$ Calcd for $C_{38}H_{45}N_3O_5SiNa$ 674.3026, found 674.3011; ^{1}H NMR (500 MHz, $CDCl_3$): δ 7.76 – 7.70 (overlapped signals, 3H), 7.48–7.37 (overlapped signals, 9H), 7.32 – 7.20 (overlapped signals, 10H), 6.92 – 6.86 (overlapped signals, 3H), 5.86 (dt, *J* = 15.4, 3.9 Hz, 1H), 5.79 (dd, *J* = 15.4, 7.8 Hz, 1H), 5.25 (hept, *J* = 6.3 Hz, 1H), 4.55 (d, *J* = 11.5 Hz, 1H), 4.51–4.44 (overlapped signals, 2H), 4.29 (overlapped signals, 2H), 3.98 (dd, *J* = 7.7,

5.4 Hz, 1H), 3.80 (overlapped signals, 6H), 3.69 – 3.55 (overlapped signals, 3H), 1.11 (s, 9H); ^{13}C NMR (125 MHz, $CDCl_3$): δ 159.3, 159.2, 149.9, 149.8, 135.5, 135.5, 135.1, 133.6, 133.5, 130.1, 130.1, 129.9, 129.7, 129.3, 129.3, 127.7, 126.1, 126.1, 126.1, 120.2, 120.2, 113.8, 113.8, 78.5, 73.0, 70.0, 69.0, 64.3, 63.6, 55.2, 55.2, 26.9, 26.8, 21.6, 19.3. To this intermediate (8 g, 12 mmol) in 30 mL of THF under nitrogen, was added 1.0 M TBAF in THF (18.4 mL, 18.4 mmol). The reaction mixture was stirred at room temperature for 12 h. Then the reaction mixture was diluted with 30 mL of water and extracted with EtOAc (2 x 60 mL). The organic portions were then combined and washed with 40 mL of sat brine solution, dried over Na_2SO_4 and solvent was removed under reduced pressure. Flash column chromatography with 25% EtOAc–cyclohexane, gave **10** (4.61 g, 91%) as a colourless liquid; TLC: R_f 0.2 in 30:70 EtOAc–cyclohexane; HRMS: $[M+Na]^+$ Calcd for $C_{22}H_{27}N_3O_5Na$ 436.1848, found 436.1836; ^{1}H NMR (500 MHz, $CDCl_3$): δ 7.22 (overlapped signals, 4H), 6.865 (overlapped signals, 4H), 5.90 (dt, *J* = 15.7, 5.1 Hz, 1H), 5.68 (dd, *J* = 15.6, 7.9 Hz, 1H), 4.53 (d, *J* = 11.4 Hz, 1H), 4.49 – 4.41 (overlapped signals, 2H), 4.293 (d, *J* = 11.4 Hz, 1H), 4.19 (d, *J* = 5.1 Hz, 2H), 3.96 (dd, *J* = 8.0, 5.6 Hz, 1H), 3.80 (overlapped signals, 6H), 3.59 (overlapped signals, 3H), 1.716 (s, 1H); ^{13}C NMR (125 MHz, $CDCl_3$): δ 159.3, 159.2, 135.1, 129.9, 129.8, 129.3, 129.3, 127.3, 113.8, 113.8, 78.4, 73.0, 70.2, 68.9, 64.2, 62.7, 55.3, 21.9.

Preparation of 12 To stirred **10** (5 g, 12 mmol) in 60 mL dry DMF at 0 ^{o}C under nitrogen, was added NaH (0.87 g, 36 mmol). The mixture was stirred for 1 h at 0 ^{o}C and then **11** (Furstner et al., 2007) (7.94 g, 24.2 mmol) in 20 mL of dry DMF was added over 10 min. The mixture was stirred for 2 h and then 30 mL of water was cautiously added at 0 ^{o}C, followed by extraction with EtOAc (2 x 60 mL). The combined organic portions were dried over Na_2SO_4, and the solvent was removed under reduced pressure. Flash column chromatography with 1:9 EtOAc–cyclohexane, gave **12** (5.32 g, 92% based on recovered **10**) as a colourless liquid and also recovered **10** (1.1 g); TLC: R_f 0.5 in 20% EtOAc–cyclohexane; HRMS: $[M+Na]^+$ Calcd for $C_{33}H_{51}N_3O_6SiNa$ 636.3445, found 636.3441; ^{1}H NMR (500 MHz, CDCl3) δ 7.27 – 7.17 (overlapped signals, 4H), 6.861 (overlapped signals, 4H), 5.822 (dt, *J* = 15.7, 5.5 Hz, 1H), 5.66 (dd, *J* = 15.6, 8.1 Hz, 1H), 4.53 (d, *J* = 11.4 Hz, 1H), 4.45 (overlapped signals, 2H), 4.28 (d, *J* = 11.4 Hz, 1H), 4.01 (overlapped signals, 2H), 3.94 (dd, *J* = 8.0, 5.3 Hz, 1H), 3.80 (overlapped signals, 6H), 3.66 – 3.51 (overlapped signals, 6H), 3.43 (overlapped signals, 2H), 1.65–1.59 (overlapped signals, 2H), 1.54 (overlapped signals, 2H), 1.39 (overlapped signals, 2H), 0.89 (s, 9H), 0.044 (overlapped signals, 6H); ^{13}C NMR (125 MHz, $CDCl_3$) δ 159.3, 159.2, 133.0, 130.0, 129.9,

129.3, 129.3, 128.4, 113.8, 113.8, 78.5, 73.0, 70.5, 70.5, 70.1, 69.0, 64.2, 63.1, 55.2, 32.7, 29.5, 26.9, 26.0, 22.4, 18.3, -5.3.

Preparation of 13 To stirred **12** (5.0 g, 8.4 mmol) in 40 mL of THF under nitrogen, was added TBAF 1M in THF (12.2 mL, 12.2 mmol). The reaction mixture was stirred for 2 h at room temperature. Then the mixture was diluted with 20 mL of water and extracted with EtOAc (2 x 60 mL), the organic portions were combined and washed with 30 mL of sat brine solution and the organic layer dried over Na_2SO_4. The solvent was removed under reduced pressure and flash column chromatography, with 2:3 EtOAc–cyclohexane, gave intermediate alcohol (3.82 g, 94%) as a colourless liquid; TLC: R_f 0.4 in 1:1 EtOAc–cyclohexane; HRMS: $[M+Na]^+$ Calcd for $C_{27}H_{37}N_3O_6Na$ 522.2580, found 522.2576; ^1H NMR (500 MHz, $CDCl_3$) δ 7.22 (overlapped signals, 4H), 6.86 (overlapped signals, 4H), 5.82 (dt, *J* = 15.6, 5.4 Hz, 1H), 5.66 (dd, *J* = 15.6, 8.1 Hz, 1H), 4.53 (d, *J* = 11.5Hz, 1H), 4.50 (overlapped signals, 2H), 4.28 (d, *J* = 11.4 Hz, 1H), 4.01 (overlapped signals, 2H), 3.94 (dd, *J* = 8.0, 5.4 Hz, 1H), 3.80 (overlapped signals, 6H), 3.68 – 3.57 (overlapped signals, 4H), 3.54 (dd, *J* = 9.8, 6.9 Hz, 1H), 3.41 (overlapped signals, 2H), 1.67 – 1.58 (overlapped signals, 5H), 1.44 (overlapped signals, 2H); ^{13}C NMR (125 MHz, $CDCl_3$) δ 159.3, 159.2, 132.9, 129.9, 129.9, 129.3, 129.3, 128.5, 113.8, 113.8, 78.5, 73.0, 70.4, 70.3, 70.2, 69.0, 64.2, 62.8, 55.2, 32.5, 29.4, 22.4. To this alcohol intermediate (3.6 g, 7.2 mmol) in 40 mL dry dichloromethane under nitrogen at 0 °C, was added NEt_3 (3.01 mL, 21.61 mmol) followed by slow addition of BzCl (1.25 mL, 10.8 mmol). The reaction mixture was stirred for 1 h at room temperature, and then, cautiously, 5 mL of sat aq $NaHCO_3$ solution at 0 °C was added. The mixture was then diluted with 15 mL water and extracted with dichloromethane (2 x 50 mL). The combined organic portions were dried over Na_2SO_4 and the solvent was removed under reduced pressure. Flash column chromatography with 15% EtOAc–cyclohexane, gave a benzoylated intermediate (4.02 g, 93%) as a colourless syrup; TLC: R_f 0.3 in 20% EtOAc–cyclohexane; HRMS: $[M+Na]^+$ Calcd for $C_{34}H_{41}N_3O_7Na$ 626.2842, found 626.2833; ^1H NMR (500 MHz, $CDCl_3$) δ 8.04 (overlapped signals, 2H), 7.541 (t, *J* = 7.4 Hz, 1H), 7.425 (overlapped signals, 2H), 7.21 (overlapped signals, 4H), 6.90 – 6.82 (overlapped signals, 4H), 5.82 (dt, *J* = 15.6, 5.4 Hz, 1H), 5.66 (dd, *J* = 15.7, 8.0 Hz, 1H), 4.53 (d, *J* = 11.4 Hz, 1H), 4.44 (d, *J* = 2.6 Hz, 2H), 4.33 (overlapped signals, 2H), 4.28 (d, *J* = 11.4 Hz, 1H), 4.015 (overlapped signals, 2H), 3.94 (dd, *J* = 8.0, 5.3 Hz, 1H), 3.79 (overlapped signals, 6H), 3.64 – 3.56 (overlapped signals, 2H), 3.533 (dd, *J* = 9.6, 6.7 Hz, 1H), 3.46 (overlapped signals, 2H), 1.81 (overlapped signals, 2H), 1.68 (overlapped signals, 2H), 1.52 (overlapped signals, 2H); ^{13}C NMR (125 MHz, $CDCl_3$) δ

166.6, 159.3, 159.2, 132.9, 132.8, 130.4, 129.9, 129.9, 129.5, 129.3, 129.3, 128.5, 128.3, 113.8, 113.8, 78.4, 73.0, 70.5, 70.2, 70.2, 69.0, 64.9, 64.2, 55.2, 29.3, 28.6, 22.8.

To this benzoylated intermediate (3.8 g, 6.3 mmol) in dry 80 mL dichloromethane under nitrogen at 0 ^{o}C, was added slowly 8 mL of trifluoroacetic acid. Stirring was continued at the same temperature for 2 h and then the volatile components were removed under reduced pressure at room temperature (avoiding heating). Flash column chromatography with 50% EtOAc–cyclohexane gave a diol (1.87 g, 82%) as a colourless syrup; TLC: R_f 0.1 in 50% EtOAc–cyclohexane; HRMS: $[M+Na]^+$ Calcd for $C_{18}H_{25}N_3O_5Na$ 386.1692, found 386.1677; ^{1}H NMR (500 MHz, $CDCl_3$) δ 8.03 (overlapped signals, 2H), 7.55 (t, *J* = 7.2 Hz, 1H), 7.43 (overlapped signals, 2H), 5.90 (dt, *J* = 15.6, 5.3 Hz, 1H), 5.79 (dd, *J* = 15.6, 6.5 Hz, 1H), 4.35 – 4.28 (overlapped signals, 3H), 3.99 (overlapped signals, 2H), 3.80 (overlapped signals, 2H), 3.49 (t, *J* = 5.2 Hz, 1H), 3.46 (overlapped signals, 2H), 2.53 (overlapped signals, 2H), 1.79 (overlapped signals, 2H), 1.66 (overlapped signals, 2H), 1.52 (overlapped signals, 2H); ^{13}C NMR (125 MHz, $CDCl_3$) δ 166.8, 132.9, 130.6, 130.5, 130.3, 129.5, 128.3, 72.9, 70.4, 70.4, 66.4, 65.0, 62.4, 29.2, 28.5, 22.7

To this diol (3.6 g, 9.9 mmol) in 30 mL of dry dichloromethane at 0 ^{o}C under nitrogen, were added imidazole (1.0 g, 15 mmol) and TBSCl (1.6 g, 11 mmol). The reaction mixture stirred for 1 h at 0 ^{o}C and was then diluted with 15 mL of water and extracted with dichloromethane (2 x 25 mL), the combined organic portionswas dried over Na_2SO_4 and the solvent was removed under reduced pressure. Then flash column chromatography with 20% EtOAc–cyclohexane, gave TBS protected intermediate (3.7 g, 78%) as a colourless syrup; TLC: R_f 0.3 in 20% EtOAc–cyclohexane; HRMS: $[M+Na]^+$ Calcd for $C_{24}H_{39}N_3O_5NaSi$ 500.2557, found 500.2556; ^{1}H NMR (500 MHz, $CDCl_3$) δ 8.07 – 8.01 (overlapped signals, 2H), 7.55 (t, *J* = 7.5 Hz, 1H), 7.43 (overlapped signals, 2H), 5.89 (dtd, *J* = 15.5, 5.4, 1.1 Hz, 1H), 5.77 (ddt, *J* = 15.5, 6.3, 1.4 Hz, 1H), 4.32 (overlapped signals, 2H), 4.28 (t, *J* = 5.3 Hz, 1H), 3.99 (overlapped signals, 2H), 3.86 – 3.79 (overlapped signals, 2H), 3.45 (overlapped signals, 2H), 3.41 (apparent q, *J* = 5.4 Hz, 1H), 2.529 (d, *J* = 5.2 Hz, 1H), 1.835 – 1.758 (overlapped signals, 2H), 1.654 (overlapped signals, 2H), 1.56 – 1.48 (overlapped signals, 2H), 0.90 (s, 9H), 0.086 (s, 6H); ^{13}C NMR (125 MHz, $CDCl_3$) δ 166.7, 132.8, 130.6, 130.3, 129.5, 128.3, 72.4, 70.5, 70.2, 66.1, 64.9, 63.5, 29.4, 28.6, 25.7, 22.7, 18.1, -5.6, -5.6.

To a stirred solution of this TBS protected intermediate (3.5 g, 7.3 mmol) in 30 mL of MeOH, was added K_2CO_3 (0.1 g, 0.7 mmol) at room temperature. The reaction mixture was stirred at room temperature for 2 h and then the solvent was removed under reduced pressure at room temperature, avoiding heating. Flash column chromatography with 50% EtOAc–cyclohexane, gave a primary alcohol intermediate (2.3 g, 86%) as a colourless syrup; TLC: R_f 0.2 in 60% EtOAc–cyclohexane; HRMS: $[M+Na]^+$ Calcd for $C_{17}H_{35}N_3O_4NaSi$ 396.2295, found 393.2291; ^1H NMR (500 MHz, $CDCl_3$) δ 5.89 (dt, J = 15.7, 5.4 Hz, 1H), 5.78 (dd, J = 15.6, 6.3 Hz, 1H), 4.29 (t, J = 5.9 Hz, 1H), 3.99 (overlapped signals, 2H), 3.83 (overlapped signals, 2H), 3.65 (overlapped signals, 2H), 3.45 (overlapped signals, 2H), 3.42 (d, J = 5.8 Hz, 1H), 1.61 (overlapped signals, 4H), 1.44 (overlapped signals, 2H), 0.91 (s, 9H), 0.09 (overlapped s, 6H); ^{13}C NMR (125 MHz, $CDCl_3$) δ 130.7, 130.3, 72.4, 70.5, 70.4, 66.1, 63.6, 62.8, 32.4, 29.4, 25.7, 22.4, 18.1-5.6.

To this primary alcohol (2.0 g, 5.3 mmol) in 30 mL of dry dichloromethane at 0 ^oC under nitrogen, were added imidazole (0.54 g, 8.0 mmol) and TBDPSCl (1.5 mL, 5.9 mmol). The reaction mixture was stirred for 2 h at 0 ^oC, then diluted with 15 mL of water, and extracted with dichloromethane (2x30 mL). The combined organic portions were dried over Na_2SO_4 and the solvent was removed under reduced pressure. Flash column chromatography with 10% EtOAc–cyclohexane, gave the TBDPS protected intermediate (2.8 g, 86%) as a colourless syrup; TLC: R_f 0.2 in 10% EtOAc–cyclohexane; HRMS: $[M+Na]^+$ Calcd for $C_{33}H_{53}N_3O_4NaSi_2$ 634.3472, found 634.3475; ^1H NMR (500 MHz, $CDCl_3$) δ 7.69 – 7.64 (overlapped signals, 4H), 7.44 – 7.35 (overlapped signals, 6H), 5.89 (dt, J = 15.4, 5.4 Hz, 1H), 5.77 (dd, J = 15.5, 6.4 Hz, 1H), 4.29 (apparent q, J = 5.7 Hz, 1H), 3.98 (overlapped signals, 2H), 3.87 – 3.78 (overlapped signals, 2H), 3.66 (overlapped signals, 2H), 3.41 (overlapped signals, 3H), 2.42 (d, J = 5.3 Hz, 1H), 1.62 – 1.56 (overlapped signals, 4H), 1.41 (overlapped signals, 2H), 1.04 (s, 9H), 0.91 (s, 9H), 0.09 (overlapped s, 6H); ^{13}C NMR (125 MHz, $CDCl_3$) δ 135.5, 134.1, 130.4, 130.4, 129.5, 127.6, 72.5, 70.6, 70.4, 66.0, 63.8, 63.6, 32.4, 29.5, 26.9, 25.7, 22.4, 19.2, 18.1, -5.6, -5.6.

To this TBDPS protected intermediate, which had a secondary alcohol (2.5 g, 4.1 mmol), in 40 mL of dry dichloromethane at 0 ^oC under nitrogen, were added 5 mL pyridine and, slowly, benzoyl chloride (0.94 mL, 8.2 mmol). The mixture was stirred for 2 h at room temperature, then diluted with 20 mL of sat aq $NaHCO_3$, and extracted with dichloromethane (2 x 30 mL), the combined organic portions dried over Na_2SO_4 and the solvent was removed under reduced pressure. Flash column chromatography with

5% EtOAc–cyclohexane gave a benzoylated intermediate (2.6 g, 91%) as a colourless syrup; TLC: R_f 0.5 in 10% EtOAc–cyclohexane; HRMS: $[M+Na]^+$ Calcd for $C_{40}H_{57}N_3O_5NaSi_2$ 738.3734, found 738.3712; ^1H NMR (500 MHz, $CDCl_3$) δ 8.05 (overlapped signals, 2H), 7.65 (overlapped signals, 4H), 7.61 – 7.52 (m, 1H), 7.47 – 7.42 (overlapped signals, 2H), 7.41 – 7.33 (overlapped signals, 5H), 7.30 – 7.09 (m, 1H), 6.01 – 5.93 (m, 1H), 5.86 – 5.79 (m, 1H), 5.68 (t, *J* = 5.7 Hz, 1H), 3.99 (overlapped signals, 2H), 3.84 – 3.77 (m, 1H), 3.73 (overlapped signals, 2H), 3.68 – 3.60 (overlapped signals, 2H), 3.39 (overlapped signals, 2H), 1.62 – 1.56 (overlapped signals, 4H), 1.40 (overlapped signals, 2H), 1.03 (s, 9H), 0.90 (s, 9H), 0.06 (overlapped signals, 6H); ^{13}C NMR (125 MHz, $CDCl_3$) δ 165.1, 135.5, 134.1, 133.3, 133.2, 129.7, 129.5, 128.4, 127.6, 125.2, 73.4, 70.6, 70.2, 65.6, 63.8, 62.8, 32.4, 29.4, 26.8, 25.7, 22.3, 19.2, 18.1, -5.6, -5.6.

To this benzoylated intermediate (4 g, 5.6 mmol) in 120 mL of THF, was added 1M HCl (5.6 mL, 5.6 mmol). The reaction mixture was stirred for 48 h and was then 10 mL of triethylamine was added. Then the volatile components were removed under reduced pressure, while not heating above room temperatura (20 C). Column chromatography with 1:19 EtOAc–cyclohexane gave the recovered unreacted intermediate (1 g); subsequent elution with 30:70 EtOAc–cyclohexane gave **13** (2.0 g, 81%) as a syrup; TLC: R_f 0.2 in 30:70 EtOAc–cyclohexane; HRMS: $[M+Na]^+$ Calcd for $C_{34}H_{43}N_3O_5NaSi$ 624.2870, found 624.2886; ^1H NMR (500 MHz, $CDCl_3$) δ 8.09 – 8.03 (overlapped signals, 2H), 7.69 – 7.63 (overlapped signals, 4H), 7.49 – 7.33 (overlapped signals, 8H), 6.02 (dt, *J* = 15.6, 5.1 Hz, 1H), 5.88 (ddt, *J* = 15.5, 7.3, 1.7 Hz, 1H), 5.71 (dd, *J* = 7.2, 5.0 Hz, 1H), 4.03 – 3.96 (overlapped signals, 2H), 3.83 (dt, *J* = 6.9, 4.5 Hz, 1H), 3.78 (ddd, *J* = 11.7, 7.5, 4.2 Hz, 1H), 3.71 – 3.62 (overlapped signals, 3H), 3.41 (t, *J* = 6.6 Hz, 2H), 2.15 – 2.05 (m, 1H), 1.62 – 1.52 (overlapped signals, 4H), 1.43 – 1.36 (overlapped signals, 2H), 1.04 (s, 9H); ^{13}C NMR (125 MHz, $CDCl_3$) δ 165.4, 135.5, 134.1, 133.6, 133.4, 129.8, 129.5, 128.5, 127.6, 125.0, 73.7, 70.7, 70.1, 66.0, 63.8, 61.8, 32.3, 29.4, 26.9, 26.9, 22.3, 19.2.

Preparation of 14 To stirred **6** (3.7 g, 5.0 mmol) and **13** (2 g, 3.3 mmol) in 40 mL dry dichloromethane at 0 °C under nitrogen, was added slowly, $BF_3.Et_2O$ (0.6 mL, 5.0 mmol) in 3 mL of dry dichloromethane, and the mixture stirred for 1 h at 0 °C. Then it was diluted with 20 mL of sat aq $NaHCO_3$, and extracted with dichloromethane (2 x 30 mL). The combined organic portions were dried over Na_2SO_4, the solvent was removed under reduced pressure and flash column chromatography of the residue with 18:82 EtOAc–cyclohexane, gave **14** (3.2 g, 83%) as a white solid; TLC: R_f 0.6 in 50:50 EtOAc–cyclohexane; HRMS: $[M+Na]^+$ Calcd for $C_{68}H_{69}N_3O_{14}NaSi$ 1202.4447, found 1202.4420; ^1H

NMR (500 MHz, $CDCl_3$) δ 8.15 – 8.09 (overlapped signals, 2H), 8.07 – 7.95 (overlapped signals, 6H), 7.80 (dd, *J* = 8.3, 1.4 Hz, 2H), 7.70 – 7.64 (overlapped signals, 4H), 7.65 – 7.60 (m, 1H), 7.58 – 7.47 (overlapped signals, 4H), 7.46 – 7.34 (overlapped signals, 13H), 7.28 – 7.22 (overlapped signals, 2H), 6.01 (dd, *J* = 3.5, 1.1 Hz, 1H), 5.89 – 5.82 (overlapped signals, 2H), 5.82 – 5.75 (m, 1H), 5.71 (dd, *J* = 6.9, 4.4 Hz, 1H), 5.63 (dd, *J* = 10.4, 3.4 Hz, 1H), 4.90 (d, *J* = 7.9 Hz, 1H), 4.62 (dd, *J* = 11.0, 6.2 Hz, 1H), 4.39 (t, *J* = 5.5 Hz, 1H), 4.36 – 4.30 (m, 1H), 4.09 (dd, *J* = 10.2, 6.7 Hz, 1H), 4.05 – 3.98 (m, 1H), 3.92 – 3.81 (overlapped signals, 2H), 3.76 (dd, *J* = 10.2, 5.4 Hz, 1H), 3.66 (overlapped signals, 2H), 3.34 (overlapped signals, 2H), 1.55 (overlapped signals, 4H), 1.43 – 1.34 (overlapped signals, 2H), 1.05 (s, 9H); ^{13}C NMR (125 MHz, $CDCl_3$) δ 166.0, 165.6, 165.5, 165.1, 164.9, 135.6, 134.1, 133.8, 133.6, 133.3, 133.2, 133.2, 130.1, 129.8, 129.8, 129.8, 129.7, 129.5, 129.4, 129.3, 129.0, 128.7, 128.7, 128.5, 128.4, 128.4, 128.3, 127.6, 124.5, 101.2, 73.7, 71.6, 71.4, 70.7, 70.0, 69.6, 68.1, 67.9, 63.8, 63.4, 61.9, 32.4, 29.4, 26.9, 26.9, 22.3, 19.2.

Preparation of 15 To stirred **14** (2.8 g, 2.3 mmol) in 60 mL of 1:5 THF-H_2O, was added PPh_3 (1.9 g, 7.1 mmol). The reaction mixture was stirred for 14 h at 40 °C, then diluted with 10 mL of sat aq NaCl, and extracted with EtOAc (2 x 30 mL). The combined organic portions were dried over Na_2SO_4 and the solvent was removed under reduced pressure and the residue used in the next step without further purification. Thus, to the residue were added butyric acid (0.3 mL, 3.6 mmol) in 25 mL MeCN, DIPEA (3 mL) and EDCI (1.9 mL, 10.8 mmol). The mixture was stirred for 2 h at room temperature, then diluted with 10 mL of sat aq $NaHCO_3$, and extracted with EtOAc (2 x 30 mL). The combined organic portions were dried over Na_2SO_4 and the solvent was removed under reduced pressure. Flash chromatography of the residue with 22:78 EtOAc–cyclohexane gave **15** (1.8 g, 61%) as a white solid; TLC: R_f 0.5 in 50% EtOAc–cyclohexane; HRMS: $[M+Na]^+$ Calcd for $C_{72}H_{77}NO_{15}NaSi$ 1246.4960, found 1246.4962; ^{1}H NMR (500 MHz, $CDCl_3$) δ 8.16 – 8.10 (overlapped signals, 2H), 8.06 – 8.01 (overlapped signals, 2H), 7.95 (overlapped signals, 4H), 7.81 – 7.75 (overlapped signals, 2H), 7.69 – 7.61 (overlapped signals, 5H), 7.57 – 7.47 (overlapped signals, 5H), 7.46 – 7.34 (overlapped signals, 13H), 7.29 – 7.22 (overlapped signals, 2H), 6.00 – 5.89 (overlapped signals, 2H), 5.84 – 5.72 (overlapped signals, 3H), 5.70 (t, *J* = 7.0 Hz, 1H), 5.64 (ddd, *J* = 10.4, 3.6, 1.6 Hz, 1H), 4.79 (dd, *J* = 7.8, 1.7 Hz, 1H), 4.59 – 4.50 (m, 1H), 4.34 (ddd, *J* = 10.8, 6.0, 1.7 Hz, 1H), 4.29 (dt, *J* = 9.5, 2.0 Hz, 1H), 4.25 (t, *J* = 6.4 Hz, 1H), 4.19 (ddd, *J* = 10.9, 6.7, 1.7 Hz, 1H), 3.94 (d, *J* = 5.6 Hz, 2H), 3.73 – 3.68 (m, 1H), 3.65 (overlapped signals, 2H), 3.41 – 3.30 (overlapped signals, 2H), 1.82 (overlapped signals, 2H), 1.55 (overlapped signals, 4H), 1.48 – 1.44 (m, 1H), 1.42 – 1.34 (overlapped signals, 3H), 1.04

(overlapped signals, 9H), 0.75 (m, 3H); ^{13}C NMR (125 MHz, $CDCl_3$) δ 172.6, 165.8, 165.5, 165.5, 165.4, 165.1, 134.1, 133.7, 133.5, 133.3, 133.3, 133.1, 132.4, 130.1, 130.1, 129.8, 129.7, 129.6, 129.5, 129.3, 129.1, 129.0, 128.7, 128.6, 128.5, 128.4, 128.3, 1276, 127.2, 101.0, 73.2, 71.3, 70.5, 70.5, 70.2, 67.9, 67.4, 63.8, 61.6, 50.6, 38.3, 32.4, 29.4, 26.9, 26.9, 22.3, 19.2, 18.9, 13.6.

Preparation of 16 To stirred **15** (1.4 g, 1.1 mmol) in 30 mL of 1:1 dichloromethane-MeOH, was added *p*TSA (0.2 g, 1.1 mmol) and the mixture was stirred for 16 h at room temperature. Then the mixture was diluted with 15 mL of sat aq $NaHCO_3$ and extracted with EtOAc (2 x 25 mL). The combined organic portions were dried over Na_2SO_4 and the solvent was removed under reduced pressure. Flash column chromatography with 80% EtOAc–cyclohexane, gave the primary alcohol intermediate (0.97 g, 86%) as a white solid; TLC: R_f 0.3 in 80:20 EtOAc–cyclohexane; HRMS: $[M+Na]^+$ Calcd for $C_{56}H_{59}NO_{15}Na$ 1008.3782, found 1008.3765; ^{1}H NMR (500 MHz, $CDCl_3$) δ 8.12 (overlapped signals, 2H), 8.03 (overlapped signals, 2H), 7.94 (overlapped signals, 4H), 7.77 (overlapped signals, 2H), 7.64 (dd, J = 8.4, 6.6 Hz, 1H), 7.51 (overlapped signals, 5H), 7.45 – 7.32 (overlapped signals, 7H), 7.28 – 7.20 (overlapped signals, 2H), 5.99 – 5.90 (overlapped signals, 3H), 5.87 – 5.77 (m, 1H), 5.74 (ddd, J = 9.8, 7.7, 1.8 Hz, 1H), 5.69 (t, J = 7.2 Hz, 1H), 5.66 – 5.60 (m, 1H), 4.80 (dd, J = 7.9, 1.8 Hz, 1H), 4.56 (m, 1H), 4.30 (overlapped signals, 2H), 4.24 (t, J = 6.4 Hz, 1H), 4.16 (ddd, J = 10.9, 6.6, 1.8 Hz, 1H), 3.99 – 3.89 (overlapped signals, 2H), 3.70 (dt, J = 9.8, 2.5 Hz, 1H), 3.61 (overlapped signals, 2H), 3.39 (overlapped signals, 2H), 1.90 – 1.78 (overlapped signals, 2H), 1.63 – 1.51 (overlapped signals, 4H), 1.43 (overlapped signals, 4H), 0.75 (m, 3H); ^{13}C NMR (125 MHz, $CDCl_3$) δ 172.7, 165.8, 165.5, 165.5, 165.5, 165.1, 133.7, 133.6, 133.3, 133.3, 133.1, 132.4, 130.1, 130.1, 129.7, 129.7, 129.6, 129.3, 129.0, 129.0, 128.7, 128.7, 128.6, 128.5, 128.4, 128.3, 127.1, 100.9, 73.1, 71.3, 71.3, 70.3, 70.2, 67.9, 67.3, 62.6, 61.6, 50.6, 38.3, 32.4, 29.3, 22.5, 18.9, 13.5. To this intermediate (1.5 g, 1.5 mmol) in 25 mL of dry dichloromethane, was added 3 mL of triethylamine and MsCl (0.35 mL, 4.5 mmol) and the mixture was stirred for 30 min at room temperature. It was then diluted with 10 mL of sat aq $NaHCO_3$, and extracted with dichloromethane (2 x 20 mL), the combined organic portions were dried over Na_2SO_4 and the solvent was removed under reduced pressure. The residue, used without further purification, was taken up in 15 mL dry DMF, NaN_3 (0.29 mg, 4.5 mmol) was added and then the reaction mixture was stirred at 80 ^{o}C for 3 h. It was then diluted with 50 mL of H_2O, and extracted with EtOAc (3 x 20 mL) and the combined organic portions were dried over Na_2SO_4. The solvent was removed under reduced pressure and flash column chromatography of the residue with 25:75 EtOAc–cyclohexane, gave **16** (1.2 g, 78%) as a white solid; TLC: R_f 0.2 in 20:80 EtOAc–cyclohexane; HRMS: $[M+Na]^+$ Calcd for

$C_{56}H_{58}N_4O_{14}Na$ 1033.3847, found 1033.3883; ^{1}H NMR (500 MHz, $CDCl_3$) δ 8.15 – 8.10 (overlapped signals, 2H), 8.06 – 8.01 (overlapped signals, 2H), 7.98 – 7.92 (overlapped signals, 4H), 7.80 – 7.75 (overlapped signals, 2H), 7.68 – 7.61 (m, 1H), 7.52 (overlapped signals, 5H), 7.45 – 7.34 (overlapped signals, 7H), 7.24 (overlapped signals, 2H), 5.94 (overlapped signals, 2H), 5.84 – 5.77 (overlapped signals, 2H), 5.74 (dd, *J* = 10.4, 7.7 Hz, 1H), 5.69 (t, *J* = 6.9 Hz, 1H), 5.64 (dd, *J* = 10.4, 3.4 Hz, 1H), 4.80 (d, *J* = 7.8 Hz, 1H), 4.55 (ddt, *J* = 10.3, 7.0, 3.3 Hz, 1H), 4.33 (dd, *J* = 10.9, 6.1 Hz, 1H), 4.29 (dd, *J* = 9.6, 3.1 Hz, 1H), 4.25 (t, *J* = 6.4 Hz, 1H), 4.18 (dd, *J* = 10.9, 6.6 Hz, 1H), 3.95 (overlapped signals, 2H), 3.70 (dd, *J* = 9.6, 3.7 Hz, 1H), 3.38 (overlapped signals, 2H), 3.23 (overlapped signals, 2H), 1.89 – 1.76 (overlapped signals, 2H), 1.58 (overlapped signals, 4H), 1.50 – 1.43 (overlapped signals, 2H), 1.41 – 1.36 (overlapped signals, 2H), 1.27 (overlapped signals, 3H), 0.76 (m, 3H); ^{13}C NMR (125 MHz, $CDCl_3$) δ 172.6, 165.8, 165.5, 165.5, 165.4, 165.1, 133.7, 133.6, 133.3, 133.3, 133.1, 132.2, 130.1, 130.1, 129.7, 129.7, 129.6, 129.3, 129.1, 129.0, 128.7, 128.6, 128.4, 128.4, 128.3, 127.3, 101.0, 73.2, 71.3, 70.4, 70.2, 70.1, 67.9, 67.3, 61.6, 51.3, 50.5, 38.3, 29.2, 28.7, 26.9, 23.4, 18.9, 13.6.

Preparation of clickable sulfatide-2 To the stirred azide **16** (1.0 g, 0.99 mmol) in 15 mL of 1:1 MeOH–dichloromethane, was added K_2CO_3 (0.14 g, 0.99 mmol). The reaction mixture was stirred for 16 h, at room temperature. The solvent was removed under reduced pressure and flash chromatography using 16:84 MeOH–dichloromethane as eluant, gave the penta-ol intermediate (0.44 g, 91%) as a white solid; TLC: R_f 0.1 in 15% MeOH–dichloromethane; HRMS $[M+Na]^+$ Calcd for $C_{21}H_{38}N_4O_9Na$ 513.2536, found 513.2516; ^{1}H NMR (500 MHz, CD_3OD) δ 5.79 (dt, *J* = 15.6, 5.1 Hz, 1H), 5.73 (dd, *J* = 15.7, 6.1 Hz, 1H), 4.27 – 4.18 (overlapped signals, 2H), 4.16 (dd, *J* = 10.2, 5.0 Hz, 1H), 4.02 (dt, *J* = 8.1, 4.2 Hz, 1H), 3.95 (overlapped signals, 2H), 3.83 (d, *J* = 3.3 Hz, 1H), 3.76 (dd, *J* = 11.3, 7.0 Hz, 1H), 3.72 (dd, *J* = 11.3, 5.1 Hz, 1H), 3.62 (dd, *J* = 10.3, 3.5 Hz, 1H), 3.57 – 3.50 (overlapped signals, 2H), 3.47 (dd, *J* = 3.5, 1.3 Hz, 1H), 3.44 (overlapped signals, 2H), 3.29 (overlapped signals, 2H), 2.17 (overlapped signals, 2H), 1.61 (overlapped signals, 6H), 1.44 (overlapped signals, 2H), 0.94 (m, 3H); ^{13}C NMR (125 MHz, CD_3OD) δ 174.6, 132.5, 128.9, 103.9, 75.3, 73.4, 71.2, 70.9, 70.4, 69.7, 68.9, 68.4, 61.1, 53.4, 51.0, 37.8, 28.9, 28.3, 23.1, 19.0, 12.7. To the stirred penta-ol (0.4 g, 0.8 mmol) in 15 mL dry MeOH, was added Bu_2SnO (0.3 g, 1.2 mmol) and the mixture was heated at 60°C for 2 h. Then the solvent was removed under reduced pressure and was dried under high vacuum for 30 mins. When the residue was well dried, it was taken up in 15 mL dry THF, and then $SO_3.NEt_3$ (0.22 g, 1.6 mmol) was added. The reaction mixture was stirred for 16 h at room temperature. Then the solvent was removed under reduced pressure and the residue was dissolved in 20 mL of 1:1 MeOH–dichloromethane and

passed through a small bed of Na^+ resin. The solvent was then removed under reduced pressure and flash column chromatography with 18% MeOH–dichloromethane gave the **clickable sulfatide-2** (0.42 g, 89% 3 steps) as a white solid; TLC: R_f 0.2 in 20% MeOH–dichloromethane; HRMS $[M+Na]^+$ Calcd for $C_{21}H_{37}N_4O_{12}Na_2S$ 615.1924, found 615.1907; ^1H NMR (500 MHz, CD_3OD) δ 5.79 (dt, *J* = 15.6, 5.2 Hz, 1H), 5.73 (dd, *J* = 15.6, 6.3 Hz, 1H), 4.34 (d, *J* = 7.7 Hz, 1H), 4.27 – 4.16 (overlapped signals, 4H), 4.01 (dt, *J* = 7.9, 3.9 Hz, 1H), 3.95 (overlapped signals, 2H), 3.79 – 3.70 (overlapped signals, 3H), 3.62 (dd, *J* = 10.3, 3.4 Hz, 1H), 3.57 (t, *J* = 6.0 Hz, 1H), 3.43 (overlapped signals, 2H), 3.28 (overlapped signals, 2H), 2.17 (overlapped signals, 2H), 1.61 (overlapped signals, 6H), 1.50 – 1.39 (overlapped signals, 2H), 0.94 (m, 3H); ^{13}C NMR (125 MHz, CD_3OD) δ 174.6, 132.5, 128.9, 103.7, 80.3, 75.0, 70.8, 70.4, 69.7, 69.5, 68.4, 67.1, 61.0, 53.3, 51.0, 37.7, 28.9, 28.3, 23.1, 19.0, 12.7.

General Experimental for JGD Synthesis

All reagents were obtained from commercial sources and used without purification unless otherwise stated. THF was distilled over Na/benzophenone immediately before use. ^1H and ^{13}C NMR spectra were recorded at 500 MHz and 126 MHz respectively, on a Bruker DRX (500 MHz) NMR spectrometer. All NMR spectra were measured at 23 °C. Chemical shifts (δ) are reported in ppm and coupling constants (*J*) are reported in Hertz (Hz). The resonance multiplicities in the ^1H NMR spectra are described as "s" (singlet), "d" (doublet), "t" (triplet), and "m" (multiplet) and broad resonances are indicated by "br". Residual protic solvent of $CDCl_3$ (^1H, δ 7.26 ppm; ^{13}C, δ 77.16 ppm), and tetramethylsilane (TMS, δ 0 ppm) were used as the internal reference in the ^1H- and ^{13}C-NMR spectra. The absorptions are given in wavenumbers (cm^{-1}). Progress of the reaction was monitored by thin-layer chromatography (TLC) using silica gel 60 F_{254} precoated plates (E. Merck) and compounds were visualized by UV light with a wavelength of 254 nm. Purifications by flash column chromatography were performed using flash silica gel from Silicycle (60 Å, 40–63 μm) with the indicated eluent. The purity of the products was determined by a combination of TLC and high-pressure liquid chromatography (HPLC) was carried out using Shimadzu LC-20AD high-performance liquid chromatograph pump, a PE Nelson Analytical 900 Series integration data station, a Shimadzu SPD-10A VP (UV-*vis*, λ = 254 nm) and three AM gel columns (a guard column, two 500 Å, 10 μm columns). THF was used as solvent at the oven temperature of 23 ºC. Detection was done by UV absorbance at 254 nm. MALDI-TOF mass spectrometry was performed on a PerSeptive Biosystem-Voyager-DE (Framingham, MA) MALDI-TOF

mass spectrometer equipped with nitrogen laser (337 nm) and operating in linear mode. Internal calibration was performed using angiotensin II and bombesin as standards. The analytical sample was obtained by mixing the THF solution of the sample (5–10 mg/mL) and THF solution of the matrix (2,5-dihydroxybenzoic acid, 10 mg/mL) in a 1/5 (v/v) ratio. The prepared solution of the sample and the matrix (0.5 μL) was loaded on the MALDI plate and allowed to dry at 23 ºC before the plate was inserted into the vacuum chamber of the MALDI instrument. The laser steps and voltages applied were adjusted depending on both the molecular weight and the nature of each analyzed compound.

Synthesis of JD-A and Lac/Man-presenting GDSs

2-(3,4,5-Tris(((methyl triethylene glycol)benzoyl)oxy))-2,2-bis-hydroxymethyl-3-oxo-prop2-yn-1-yl succinate (**JD-alkyne)**, Janus dendrimer **JGD-Lac** and **JGD-Man** were synthesized and characterized as described before (Percec et al., 2013; Zhang et al., 2014).

JGD-suLac (type I). To a mixed solution of **JD-alkyne** (Kabsch, 2010) (see SI, 200 mg, 0.114 mmol) in THF (13 mL) and **suLac (type I)** (61 mg, 0.114 mmol) in water (2 mL) was added $CuSO_4{\cdot}5H_2O$ (28 mg, 0.182 mmol) in water (2 mL), and sodium ascorbate (45 mg, 0.228 mmol) in water (2 mL), successively, under nitrogen atmosphere. The reaction mixture was allowed to stir at 23 °C for 24 h. The reaction mixture was concentrated to dryness. The crude product was further purified by silica column chromatography with a mobile phase of CH_2Cl_2-MeOH, 10:1 to 5:1 to yield compound **JGD-suLac (type I)** (sodium salt) as a light-yellow gel (220 mg, 84%). Purity (HPLC): 99%+. ^{1}H NMR (500 MHz, $CDCl_3$) δ = 7.75 (s, 1H, 1×=*CH* (triazole)), 7.35 (s, 2H, 2×Ar*H*), 7.07 (s, 4H, 4×Ar*H*), 6.59 (s, 2H, 2×Ar*H*), 5.14 (m, 2H), 4.81–4.86 (m, 6H), 4.55 (m, 2H), 4.27 (m, 8H), 3.52–3.89 (m, 42H), 3.37 (m, 9H, 3×OCH_3), 2.61–2.65 (m, 4H), 1.72–1.74 (m, 8H, 4×-Ar$CH_2CH_2CH_2(CH_2)_8CH_3$), 1.40–1.41 (m, 8H, 4×-Ar$CH_2CH_2CH_2(CH_2)_8CH_3$), 1.25– (m, 64H, 4×-Ar$CH_2CH_2CH_2(CH_2)_8CH_3$), 0.85–0.88 (t, J = 6.9 Hz, 12H, 4×-Ar$(CH_2)_{11}CH_3$). ^{13}C NMR (126 MHz, $CDCl_3$) δ = 172.6, 172.3, 165.9, 165.7, 160.1, 152.1, 142.2, 131.0, 124.4, 109.0, 107.7, 106.5, 103.8, 102.4, 79.7, 75.6, 74.7, 72.3, 71.8, 70.5, 70.4, 70.4, 70.3, 69.4, 68.7, 68.2, 63.9, 61.3, 58.9, 58.8, 57.8, 50.3, 32.6, 31.7, 31.0, 29.4, 29.2, 26.0, 23.0, 22.8, 22.5, 13.6. MALDI-TOF (m/z): $[M+Na]^+$ cald. for $C_{117}H_{195}N_4Na_2O_{40}S$: 2374.29; found 2372.80.

JGD-sulfatide-1. To a mixed solution of **JD-alkyne** (see SI, 200 mg, 0.114 mmol) in THF (13 mL) and **sulfatide-1** (61 mg, 0.114 mmol) in water (2 mL) was added $CuSO_4{\cdot}5H_2O$ (28 mg, 0.114 mmol) in

water (2 mL), and sodium ascorbate (45 mg, 0.228 mmol) in water (2 mL), successively, under nitrogen atmosphere. The reaction mixture was allowed to stir at 23 °C for 24 h. The reaction mixture was concentrated to dryness. The crude product was further purified by silica column chromatography with a mobile phase of CH_2Cl_2/MeOH, 10:1 to 5:1 to yield compound **JGD-sulfatide-1** (sodium salt) as a light yellow gel 190 mg, 72%). Purity (HPLC): 99%+. ^{1}H NMR (500 MHz, $CDCl_3$) δ = 7.75 (s, 1H, 1×*CH* (triazole)), 7.33 (s, 2H, 2×Ar*H*), 7.07 (s, 4H, 4×Ar*H*), 6.59 (s, 2H, 2×Ar*H*), 5.84 (m, 1H, 1×C*H*=), 5.28 (m, 1H, 1×C*H*=), 5.14 (m, 3H, 1×O-*CH2*-TRZ and 1×C*H*=), 4.84 (m, 6H, 3×*CH2*), 4.32–4.32 (m, 10H), 4.04 (m, 2H), 3.52–3.89 (m, 46H), 3.35–3.36 (m, 9H, 3×O*CH3*), 2.65 (br, 2H, 1×COO-*CH2*CH_2CONH), 2.58 (br, 2H, COO-CH_2*CH2*CONH), 1.86 (m, 2H), 1.72–1.75 (m, 8H), 1.56 (m, 2H), 1.44 (m, 8H), 1.25–1.30 (m, 72H), 0.85–0.88 (t, 12H). ^{13}C NMR (126 MHz, $CDCl_3$) δ = 174.4, 172.6, 172.2, 166.0, 165.7, 160.4, 160.1, 152.1, 142.2, 137.3, 131.0, 124.5, 116.3, 109.0, 107.8, 106.6, 103.3, 80.0, 74.1, 72.3, 71.8, 70.4, 70.3, 69.4, 68.7, 68.3, 67.2, 63.8, 60.7, 59.4, 59.0, 58.9, 53.3, 50.8, 36.2, 36.1, 32.0, 31.8, 31.1, 29.7, 29.6, 29.6, 29.4, 29.3, 29.2, 28.3, 26.0, 25.9, 25.4, 14.1. MALDI-TOF (m/z): $[M+Na]^+$ cald. for $C_{119}H_{198}N_5Na_2O_{36}S$: 2351.33; found 2353.6.

JGD-sulfatide-2. To a mixed solution of **JD-alkyne** (see SI, 200 mg, 0.114 mmol) in THF (13 mL) and **sulfatide-2** (61 mg, 0.114 mmol) in water (2 mL) was added $CuSO_4·5H_2O$ (28 mg, 0.114 mmol) in water (2 mL), and sodium ascorbate (45 mg, 0.228 mmol) in water (2 mL), successively, under nitrogen atmosphere. The reaction mixture was allowed to stir at 23 °C for 24 h. The reaction mixture was concentrated to dryness. The crude product was further purified by silica column chromatography with a mobile phase of CH_2Cl_2/MeOH, 10:1 to 5:1 to yield compound **JGD-sulfatide-2** (sodium salt) as a light yellow gel (207 mg, 75%). Purity (HPLC): 99%+. ^{1}H NMR (500 MHz, $CDCl_3$) δ = 7.71 (s, 1H, 1×*CH* (triazole)), 7.31 (s, 2H, 2×Ar*H*), 7.08 (s, 4H, 4×Ar*H*), 6.59 (s, 2H, 2×Ar*H*), 5.74–5.78 (m, 2H, 1×C*H2*=), 5.13 (m, 3H, 1×O-*CH2*-TRZ), 4.80–4.84 (m, 6H, 3×*CH2*), 4.22–4.33 (m, 12H), 4.10 (m, 2H), 3.52–3.89 (m, 50H), 3.35–3.36 (m, 12H), 2.65 (br, 2H, 1×COO-*CH2*CH_2CONH), 2.57 (br, 2H, COO-CH_2*CH2*CONH), 2.16 (m, 2H), 2.04 (m, 6H), 1.91 (m, 2H), 1.72–1.75 (m, 8H), 1.58 (m, 4H), 1.39–1.42 (m, 8H), 1.25 (m, 70H), 0.85–0.88 (m, 15H). ^{13}C NMR (126 MHz, $CDCl_3$) δ = 174.3, 172.5, 172.1, 166.0, 165.7, 160.2, 160.2, 152.2, 142.3, 131.2, 129.0, 124.4, 109.1, 107.8, 106.6, 103.4, 79.9, 73.9, 72.3, 71.8, 71.7, 70.5, 70.4, 69.9, 69.4, 68.7, 68.3, 63.8, 59.0, 53.3, 50.4, 38.3, 31.9, 31.3, 30.0, 29.7, 29.6, 29.6, 29.4, 29.3, 29.2, 29.0, 26.0, 23.3, 22.7, 19.2, 14.1, 13.8. MALDI-TOF (m/z): $[M+Na]^+$ cald. for $C_{122}H_{204}N_5Na_2O_{37}S$: 2409.38; found 2410.05.

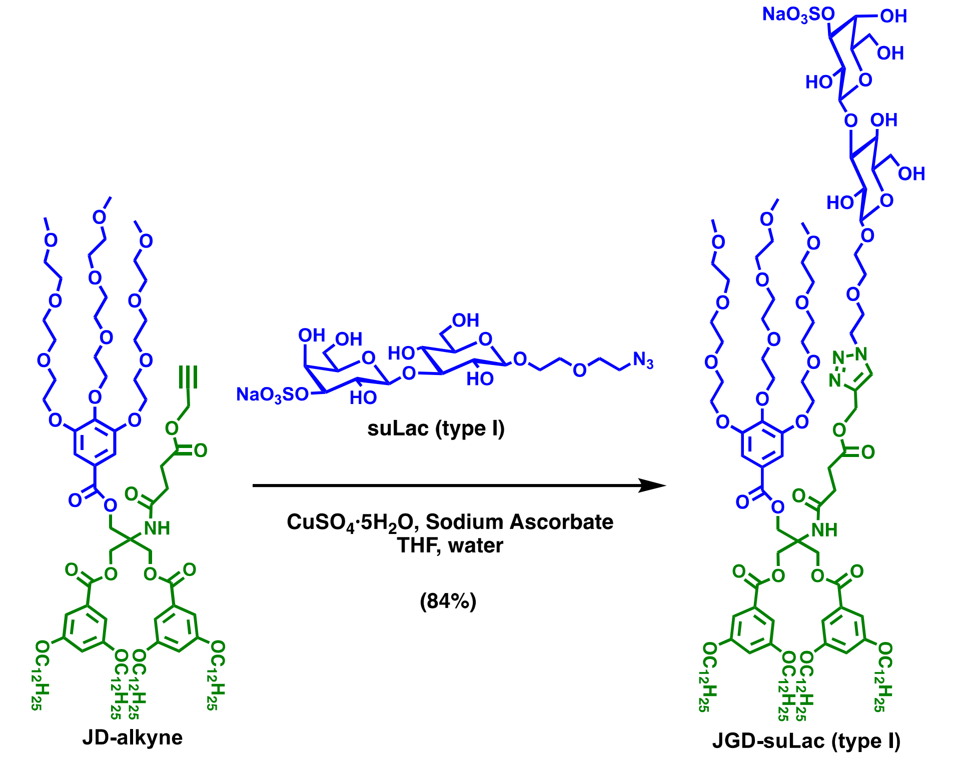

suLac (type I)
CuSO4·5H2O, Sodium Ascorbate
THF, water
(84%)
JD-alkyne
JGD-suLac (type I)

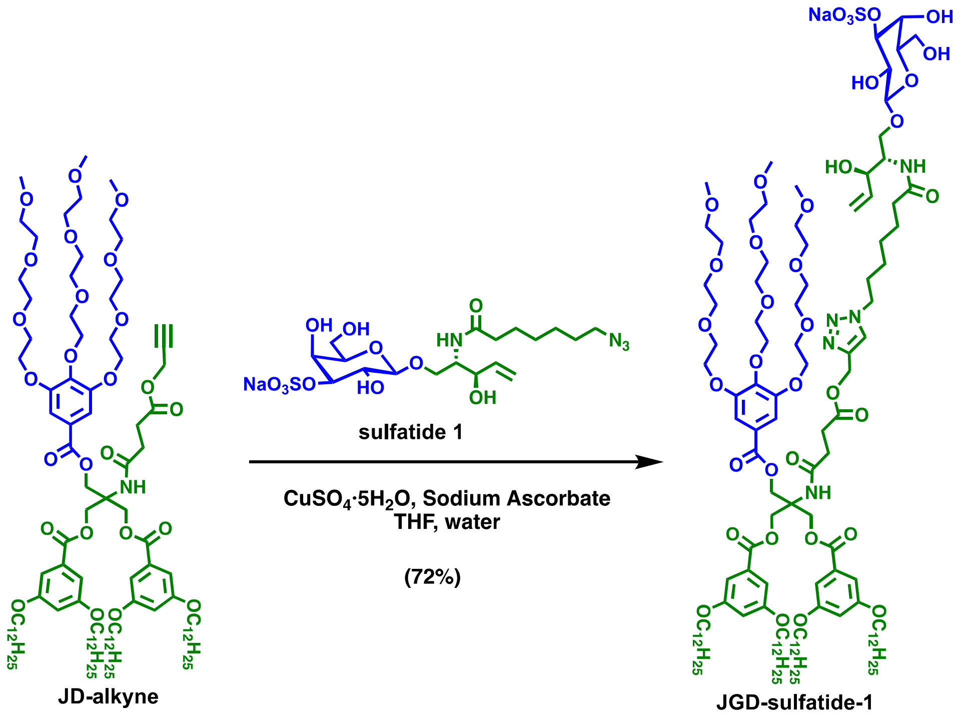

sulfatide 1
CuSO4·5H2O, Sodium Ascorbate
THF, water
(72%)
JD-alkyne
JGD-sulfatide-1

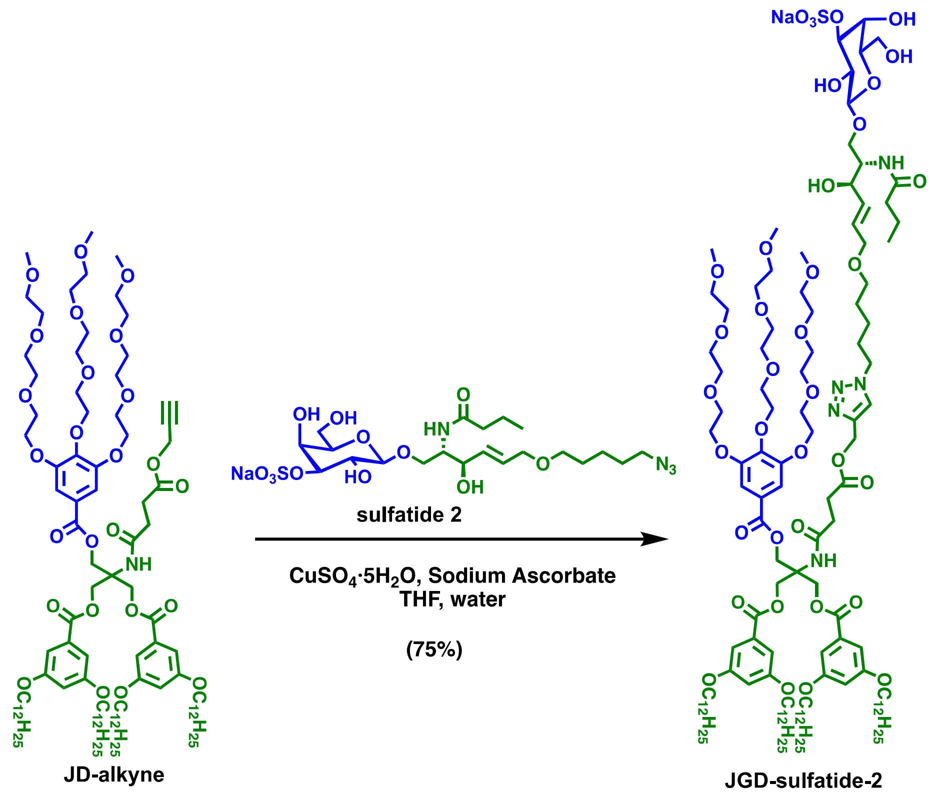

Preparation of nanoscale GDSs in injection method

A stock solution was prepared by dissolving the required amount of amphiphilic Janus glycodendrimers in ethanol. GDSs were then generated by injection of 100 μL of the stock solution into 2.0 mL PBS, followed by 5 sec vortexing.

Dynamic light scattering

DLS measurements of nanoscale GDSs were performed with a Malvern Zetasizer Nano-S instrument equipped with 4 mW He-Ne laser (633 nm) and avalanche photodiode positioned at 175° to the beam. Instrument parameters and measurement times were determined automatically. Experiments were performed in triplicate.

Aggregation assays

Aggregation assays of nanoscale GDSs with lectins were performed in semimicro disposable cuvettes at 23 °C and the course of OD was monitored at the wavelength λ = 450 nm by using a Shimadzu UV-vis spectrophotometer UV-1601 with Shimadzu/UV Probe software in kinetic mode. PBS containing

galectin (100 μL) was injected into PBS solution of GDSs (900 μL). The cuvette was shaken by hand for 1–2 s before data collection was started. The same solution of GDSs solution was used as a reference. PBS solutions of galectin were prepared before the aggregation assays and were maintained at 0 °C (ice bath) before data collection.

Cloning and expression of galectins

Generation of cDNAs and recombinant expression of galectins-1, -2, -4 (wild type, linker variants and domains), -8S and -3 and its 8S-linked homodimer were described previously (André et al., 2014; Kopitz et al., 2010, 2012; Ludwig et al., 2019; Saal et al., 2005; Xiao et al., 2018). To produce the Gal-4CC variant, full-length cDNA for two directly linked CRDs was obtained by applying a two-step PCR procedure previously described for Gal-1.[S15] In the first step, two DNA fragments were generated using the sense primer 5' gctcatatgcctgtgccatatttcgggagg '3 and the antisense primer 5' cctcccgaaatatggcacagggatctggacataggacaaggtg'3 to amplify the first fragment (the N-terminal Gal-4C domain) and the sense primer 5' caccttgtcctatgtccagatccctgtgccatatttcgggagg '3 and the antisense primer 5' cgaaagcttttagatctggacataggacaaggtg '3 for the second domain (C-terminal Gal-4C domain). In the second step, these PCR-products were used as a template to produce full-length cDNA for Gal-4CC with the sense primer 5' gctcatatgcctgtgccatatttcgggagg '3 (internal restriction site for *Nde*I underlined) and the antisense primer 5' cgaaagcttttagatctggacataggacaaggtg '3 (internal restriction site for *Hind*III underlined). In-frame ligation into the pGEMEX-1 expression vector (Promega, Munich, Germany) was followed by recombinant protein production after transformation of BL21(DE)pLysS cells (Novagen, Sigma Aldrich, Munich, Germany) (for details and yields, see Compilation below).

Human galectins obtained by recombinant expression (see Compilation below) were purified by affinity chromatography on lactosylated Sepharose 4B resin as crucial step as routinely applied.

Compilation: expression conditions and yields of recombinant galectins

Protein	Bacterial strain	Expression vector	Expression Temp. °C	IPTG (μM)	Yield (mg/L)
Gal-1	BL21 (DE3) pLysS	pGEMEX-1	37 °C	100 μM	75.20 ± 19.00
Gal-2	BL21 (DE3) pLysS	pGEMEX-1	37 °C	100 μM	10.84 ± 1.18
Gal-4	BL21 (DE3) pLysS	pGEMEX-1	30 °C	75 μM	33.56 ± 23.44
Gal-4P	BL21 (DE3) pLysS	pGEMEX-1	30 °C	75 μM	5.90 ± 1.24
Gal-4V	BL21 (DE3) pLysS	pGEMEX-1	30 °C	75 μM	13.91 ± 6.26
Gal-4N	BL21 (DE3) pLysS	pGEMEX-1	30 °C	75 μM	11.67 ± 7.32
Gal-4C	BL21 (DE3) pLysS	pGEMEX-1	30 °C	75 μM	49.72 ± 32.92

Gal-4CC	BL21 (DE3) pLysS	pGEMEX-1	30 °C	75 µM	0.80 ± 0.35
Gal-8S	BL21 (DE3) pLysS	pGEMEX-1	22 °C	100 µM	24.45 ±3.85
Gal-3—8S—Gal-3	BL21 (DE3) pLysS	pGEMEX-1	22 °C	100 µM	18.78 ± 12.69

Glycoproteins and saccharides for binding assays and assay procedure

Sources and further processing of the glycoproteins for galectin assays have been given previously in detail (Krzeminski et al., 2011). The predominant carbohydrate determinants with affinity to galectins are listed in the footnotes to Table S1 and Table S2. The *Pneumococcus* type 14 polysaccharide was a generous gift from the late Dr. E. A. Kabat (Department of Microbiology, Columbia University, NY, USA). Mono-, di- and oligosaccharides used were obtained from Dextra (Reading, Berkshire, UK) or Sigma (Munich, Germany). In detail, for the assay, the volume of each reagent solution applied to wells of the plate was 50 µL/well, and all incubations, except for coating, were performed at 20 °C. The reagents, if not indicated otherwise, were diluted with tris-buffered saline (TBS; 0.05 M Tris-HCl, 0.15 M NaCl, pH 7.35) containing 0.05 % Tween 20 (TBS-T). TBS-T was used for washing plates between incubation steps.

The surface of 96-well microtiter plate wells (Nunc-Immuno plate, Kamstrupvej, Denmark) was coated with glycoproteins dissolved in 0.05 M sodium carbonate buffer (0.05 M $NaHCO_3$/0.05 M Na_2CO_3, pH 9.6) overnight at 4 °C. After washing the plate, solution with biotinylated hGal-4 (250 ng/well) was added and the plate was then incubated for 30 min. The plates were next carefully washed to remove any free lectin, the ExtrAvidin/alkaline phosphatase solution (diluted 1:10,000; Sigma) was added thereafter to detect the specifically bound Gal-4 by its biotin moieties. After 1 h the plates were washed at least four times to remove free conjugate and then incubated with a solution of *p*-nitrophenyl phosphate (Sigma phosphatase substrate 5 mg tablets) in 0.05 M carbonate buffer, pH 9.6, containing 1 mM $MgCl_2$ (1 tablet/5 mL). The resulting absorbance was read at 405 nm in a microtiter plate reader after 24 h incubation at 20 °C in the dark with the substrate-containing solution. For inhibition studies, serially diluted inhibitor samples were mixed with an equal volume of Gal-4-containing solution. The inhibitory activity was determined from the inhibition curve and is expressed as the amount of inhibitor (ng or nmol per well) giving 50 % inhibition of the control binding.

Array testing for naturally sulfated glycans and their precursors

Probing was performed using biotinylated galectin (50 µg/mL) in a standardized procedure given previously (Blixt et al., 2004; Kutzner et al., 2019). Relative fluorescence units were recorded using the two-step procedure with fluorescent streptavidin, using this optimized protocol.

Crystallization

Crystallization trials were performed at 295 K using the sitting-drop vapor-diffusion method with commercial screening solutions including JBScreen Classic (Jena Bioscience, Jena, Germany) and Wizard Classics I–IV (Emerald Bio, Bainbridge Island, USA) in 96-well sitting-drop plates (Swissci MRC; Molecular Dimensions, Suffolk, England). Drops were set up by mixing equal volumes (0.2 µl) of protein-containing solution and reservoir solution using a Cartesian Honeybee System (Genomic Solutions, Irvine, USA) nano-dispenser robot and equilibrated against 50 µl reservoir solution. Single well-diffracting crystals were obtained in 0.1 M HEPES-NaOH Buffer at pH 7.8 containing 7.5% PEG 4000 and 15% isopropanol.

X-ray Data collection and structure determination

For data collection, crystals were cryo-protected with a cryo-solution containing the reservoir supplemented with 30 % (v/v) ethylene glycol and flash-frozen in liquid nitrogen. X-Ray data collection experiments were performed at the ALBA Synchrotron (Cerdanyola del Vallès, Spain) BL13 XALOC beamline. The data were indexed and integrated, scaled and merged using XDS (Kabsch, 2010). The structure was solved by molecular replacement using the Gal-8 N-terminal CRD structure (PDB: 5GZE) (Si et al., 2016) with Phaser (Adams et al., 2010). The initial model was first refined using Phenix-refine (Adams et al., 2010) and alternating manual building with Coot (Emsley et al., 2010). The final model was obtained by repetitive cycles of refinement; solvent molecules were added automatically and inspected visually for chemically plausible positions. PEG, acetate molecules and the sulfatide ligand, **sulfatide-1**, were added manually. The model was validated and analyzed by MolProbity (Chen et al., 2010). figures illustrating protein structure were drawn with PyMOL (DeLano, 2002). Data collection statistics are listed in Supplementary Table S3.

Supplemental References

Adams, P.D., Afonine, P.V., Bunkoczi, G., Chen, V.B., Davis, I.W., Echols, N., Headd, J.J., Hung, L.W., Kapral, G.J., Grosse-Kunstleve, R.W., *et al.* (2010). PHENIX: a comprehensive Python-based system for macromolecular structure solution. Acta Crystallogr. *D66*, 213-221.

André, S., Wang, G.N., Gabius, H.-J., and Murphy, P.V. (2014). Combining glycocluster synthesis with protein engineering: an approach to probe into the significance of linker length in a tandem-repeat-type lectin (galectin-4). Carbohydr. Res. *389*, 25-38.

Blixt, O., Head, S., Mondala, T., Scanlan, C., Huflejt, M.E., Alvarez, R., Bryan, M.C., Fazio, F., Calarese, D., Stevens, J., *et al.* (2004). Printed covalent glycan array for ligand profiling of diverse glycan binding proteins. Proc. Natl. Acad. Sci. USA *101*, 17033-17038.

Bundle, D.R., Ling, C.C., and Zhang, P. Synthetic methods for the large scale production from glucose of analogs of sphingosine, azidosphingosine, ceramides, lactosyl ceramides and glycosyl phytosphingosine. International Patent Classification: C07C 247/08, International Application Number: PCT/CA03/00832, International Publication Number: WO 03/101937 A1, 11.12.2003.

Chen, V.B., Arendall, W.B., Headd, J.J., Keedy, D.A., Immormino, R.M., Kapral, G.J., Murray, L.W., Richardson, J.S., and Richardson, D.C. (2010). MolProbity: all-atom structure validation for macromolecular crystallography. Acta Crystallogr. D*66*, 12-21.

Crich, D., Banerjee, A., Li, W., and Yao, Q. (2005). Improved synthesis of 1-benzenesulfinyl piperidine and analogs for the activation of thioglycosides in conjunction with trifluoromethanesulfonic anhydride. J. Carbohydr. Chem. *24*, 415-424.

DeLano, W. L http://www.pymol.org.

DeLano, W. L. (2002). Pymol: An open-source molecular graphics tool. CCP4 Newsletter On Protein Crystallography, *40*, 82-92

Doyle, L.M., Meany, F.B., and Murphy, P.V. (2019). Lewis acid promoted anomerisation of alkyl O- and S-xylo-, arabino- and fucopyranosides. Carbohydr. Res. *471*, 85-94.

Emsley, P., Lohkamp, B., Scott, W.G., and Cowtan, K. (2010). Features and development of Coot. Acta Crystallog.r *D66*, 486-501.

Furstner, A., Bonnekessel, M., Blank, J.T., Radkowski, K., Seidel, G., Lacombe, F., Gabor, B., and Mynott, R. (2007). Total synthesis of myxovirescin A1. Chem. Eur. J. *13*, 8762-8783.

Kabsch, W. (2010). Xds. Acta Crystallogr. *D66*, 125-132.

Kopitz, J., Bergmann, M., and Gabius, H.-J. (2010). How adhesion/growth-regulatory galectins-1 and -3 attain cell specificity: case study defining their target on neuroblastoma cells (SK-N-MC) and marked affinity regulation by affecting microdomain organization of the membrane. IUBMB Life *62*, 624-628.

Kopitz, J., Ballikaya, S., André, S., and Gabius, H.-J. (2012). Ganglioside GM1/galectin-dependent growth regulation in human neuroblastoma cells: special properties of bivalent galectin-4 and significance of linker length for ligand selection. Neurochem. Res. *37*, 1267-1276.

Krzeminski, M., Singh, T., André, S., Lensch, M., Wu, A.M., Bonvin, A.M.J.J., and Gabius, H.-J. (2011). Human galectin-3 (Mac-2 antigen): defining molecular switches of affinity to natural glycoproteins, structural and dynamic aspects of glycan binding by flexible ligand docking and putative regulatory sequences in the proximal promoter region. Biochim. Biophys. Acta. *1810*, 150-161.

Kutzner, T.J., Gabba, A., FitzGerald, F.G., Shilova, N.V., García Caballero, G., Ludwig, A.-K., Manning, J.C., Knospe, C., Kaltner, H., Sinowatz, F., *et al.* (2019). How altering the modular architecture affects aspects of lectin activity: case study on human galectin-1. Glycobiology *29*, 593-607.

Ludwig, A.-K., Michalak, M., Xiao, Q., Gilles, U., Medrano, F.J., Ma, H., FitzGerald, F.G., Hasley, W.D., Melendez-Davila, A., Liu, M., *et al.* (2019). Design-functionality relationships for adhesion/growth-regulatory galectins. Proc. Natl. Acad. Sci. USA *116*, 2837-2842.

Percec, V., Leowanawat, P., Sun, H.J., Kulikov, O., Nusbaum, C.D., Tran, T.M., Bertin, A., Wilson, D.A., Peterca, M., Zhang, S., *et al.* (2013). Modular synthesis of amphiphilic Janus glycodendrimers and their self-assembly into glycodendrimersomes and other complex architectures with bioactivity to biomedically relevant lectins. J. Am. Chem. Soc. *135*, 9055-9077.

Saal, I., Nagy, N., Lensch, M., Lohr, M., Manning, J.C., Decaestecker, C., André, S., Kiss, R., Salmon, I., and Gabius, H.-J. (2005). Human galectin-2: expression profiling by RT-PCR/immunohistochemistry and its introduction as histochemical tool for ligand localization. Histol. Histopathol. *20*, 1191-1208.

Si, Y., Wang, Y., Gao, J., Song, C., Feng, S., Zhou, Y., Tai, G., and Su, J. (2016). Crystallization of Galectin-8 Linker Reveals Intricate Relationship between the N-terminal Tail and the Linker. Int. J. Mol Sci. *17*, 2088.

Wang, W.W., Angulo-Ibanez, M., Lyu, J., Kurra, Y., Tong, Z., Wu, B., Zhang, L., Sharma, V., Zhou, J., Lin, H., *et al.* (2019). A Click Chemistry Approach Reveals the Chromatin-Dependent Histone H3K36 Deacylase Nature of SIRT7. J. Am. Chem .Soc. *141*, 2462-2473.

Xiao, Q., Ludwig, A.-K., Romanò, C., Buzzacchera, I., Sherman, S.E., Vetro, M., Vértesy, S., Kaltner, H., Reed, E.H., Möller, M., *et al.* (2018). Exploring functional pairing between surface glycoconjugates and human galectins using programmable glycodendrimersomes. Proc. Natl. Acad. Sci. USA *115*, E2509-E2518.

Zhang, S., Moussodia, R.-O., Sun, H.J., Leowanawat, P., Muncan, A., Nusbaum, C.D., Chelling, K.M., Heiney, P.A., Klein, M.L., André, S., *et al.* (2014). Mimicking biological membranes with programmable glycan ligands self-assembled from amphiphilic Janus glycodendrimers. Angew. Chem. Int. Ed. *53*, 10899-10903.

NMR Spectra

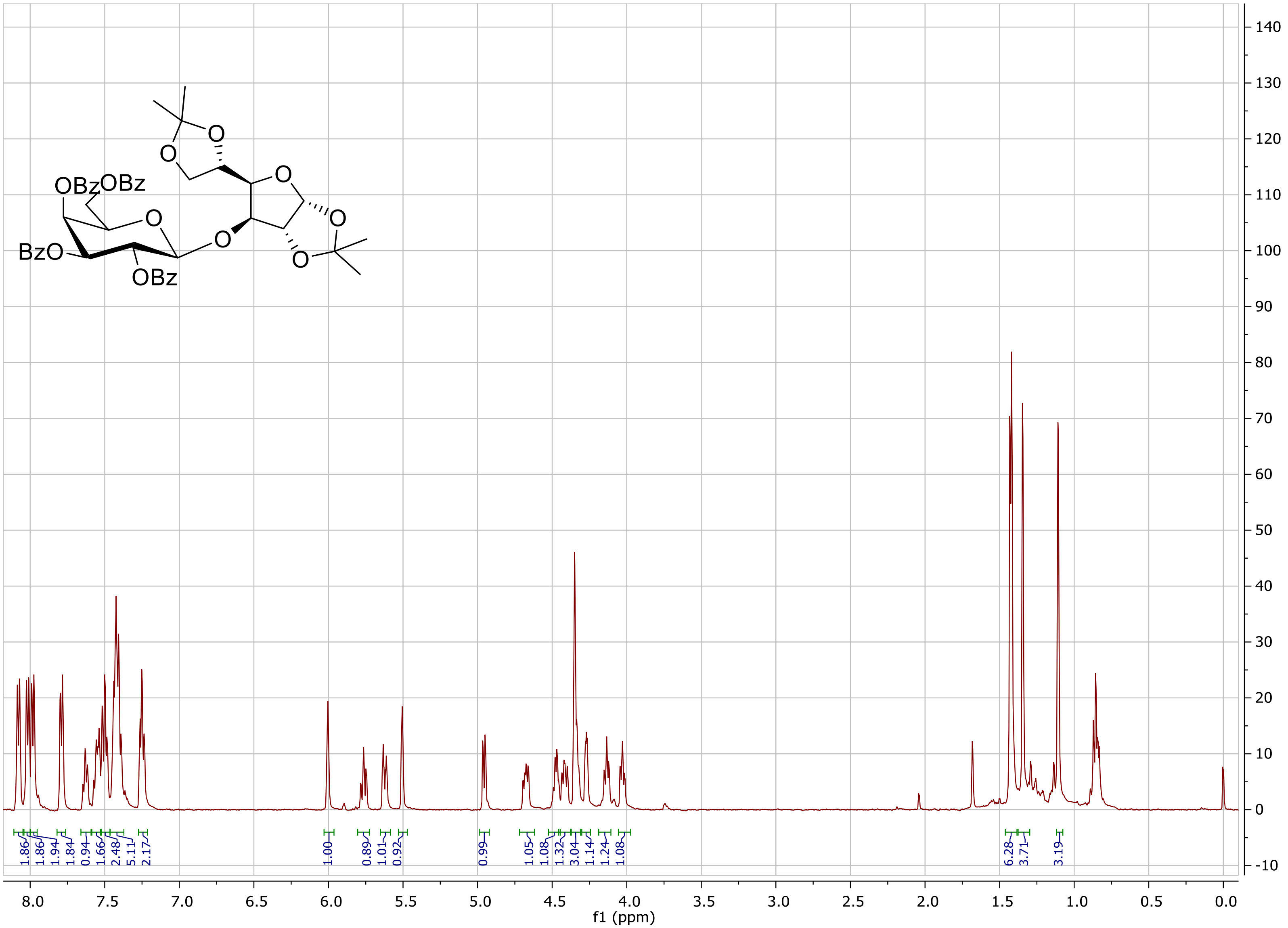

OBz
OBz
BzO
OBz
O
f1 (ppm)
1.86
1.86
1.94
1.84
0.94
1.66
2.48
5.11
2.17
1.00
0.89
1.01
0.92
0.99
1.05
1.08
1.32
3.04
1.14
1.24
1.08
6.28
3.71
3.19

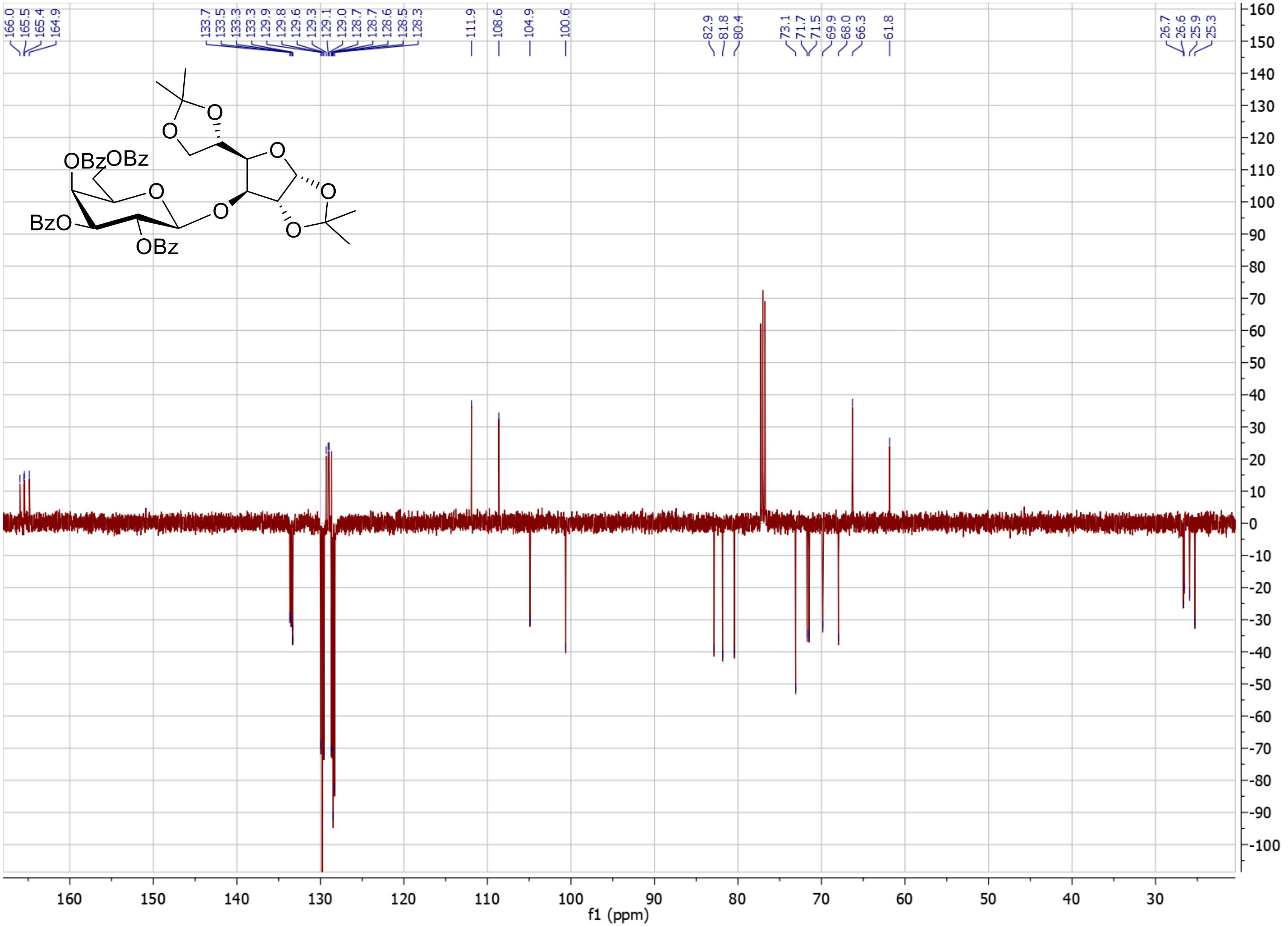

166.0
165.5
165.4
164.9
133.7
133.5
133.3
133.3
129.9
129.8
129.6
129.3
129.1
129.0
128.7
128.7
128.6
128.5
128.3
111.9
108.6
104.9
100.6
82.9
81.8
80.4
73.1
71.7
71.5
69.9
68.0
66.3
61.8
26.7
26.6
25.9
25.3
OBz
OBz
BzO
OBz
O
f1 (ppm)

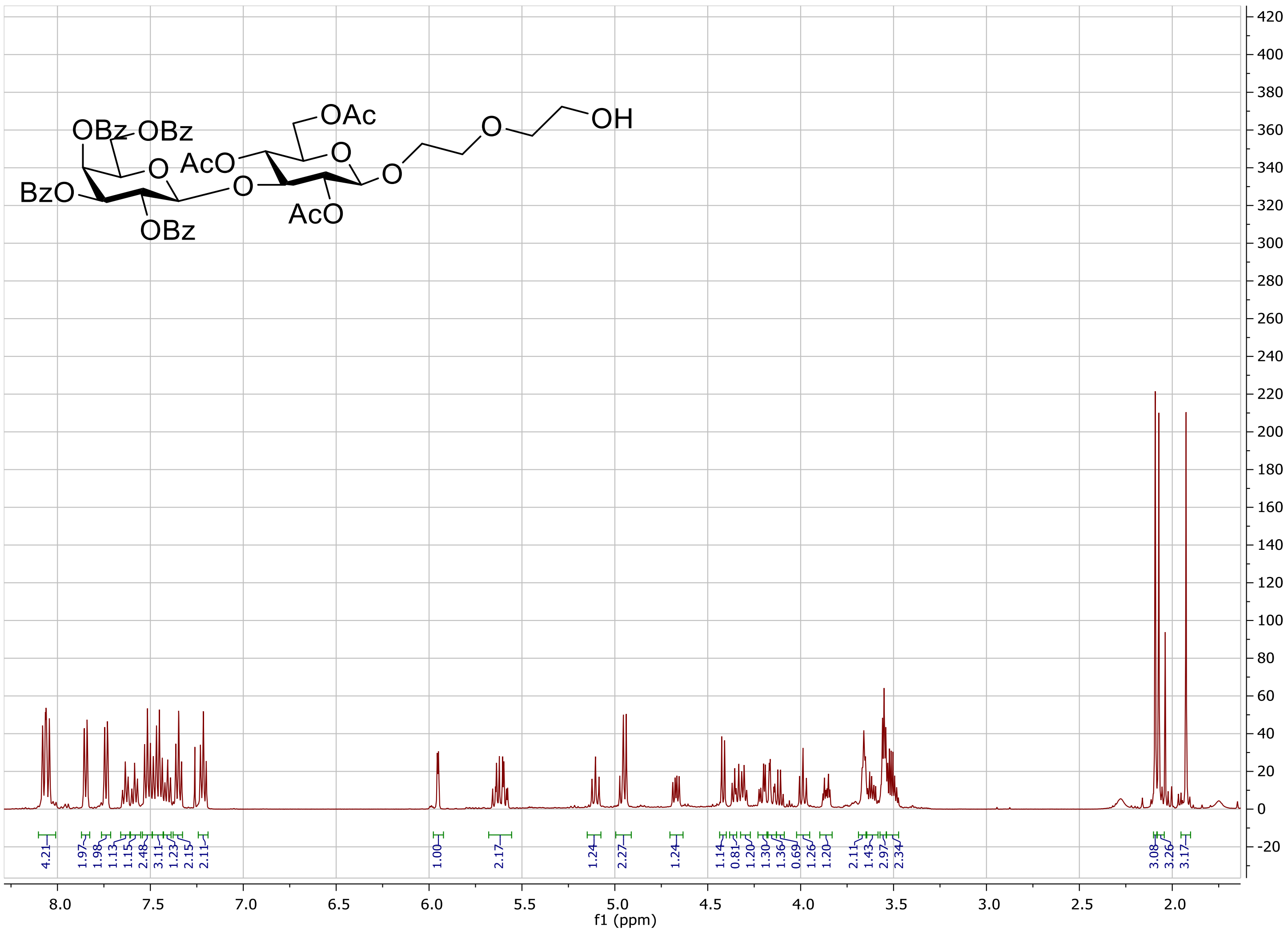

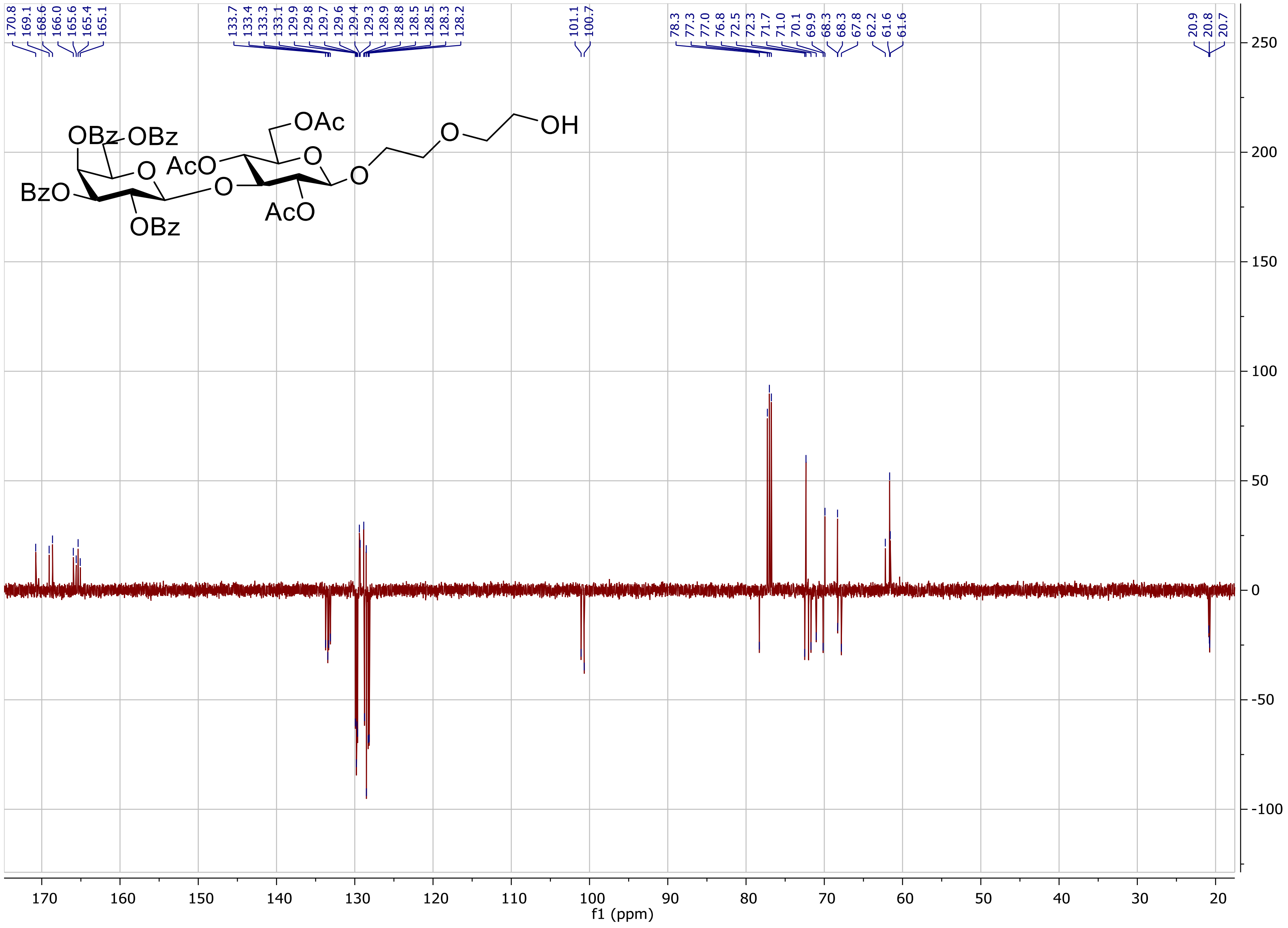
170.8
169.1
168.6
166.0
165.6
165.4
165.1
133.7
133.4
133.3
133.1
129.9
129.8
129.7
129.6
129.4
129.3
128.9
128.8
128.5
128.5
128.3
128.2
101.1
100.7
78.3
77.3
77.0
76.8
72.5
72.3
71.7
71.0
70.1
69.9
68.3
68.3
67.8
62.2
61.6
61.6
20.9
20.8
20.7
OBz
OBz
O
AcO
OAc
O
O
O
OH
BzO
O
OBz
AcO
f1 (ppm)

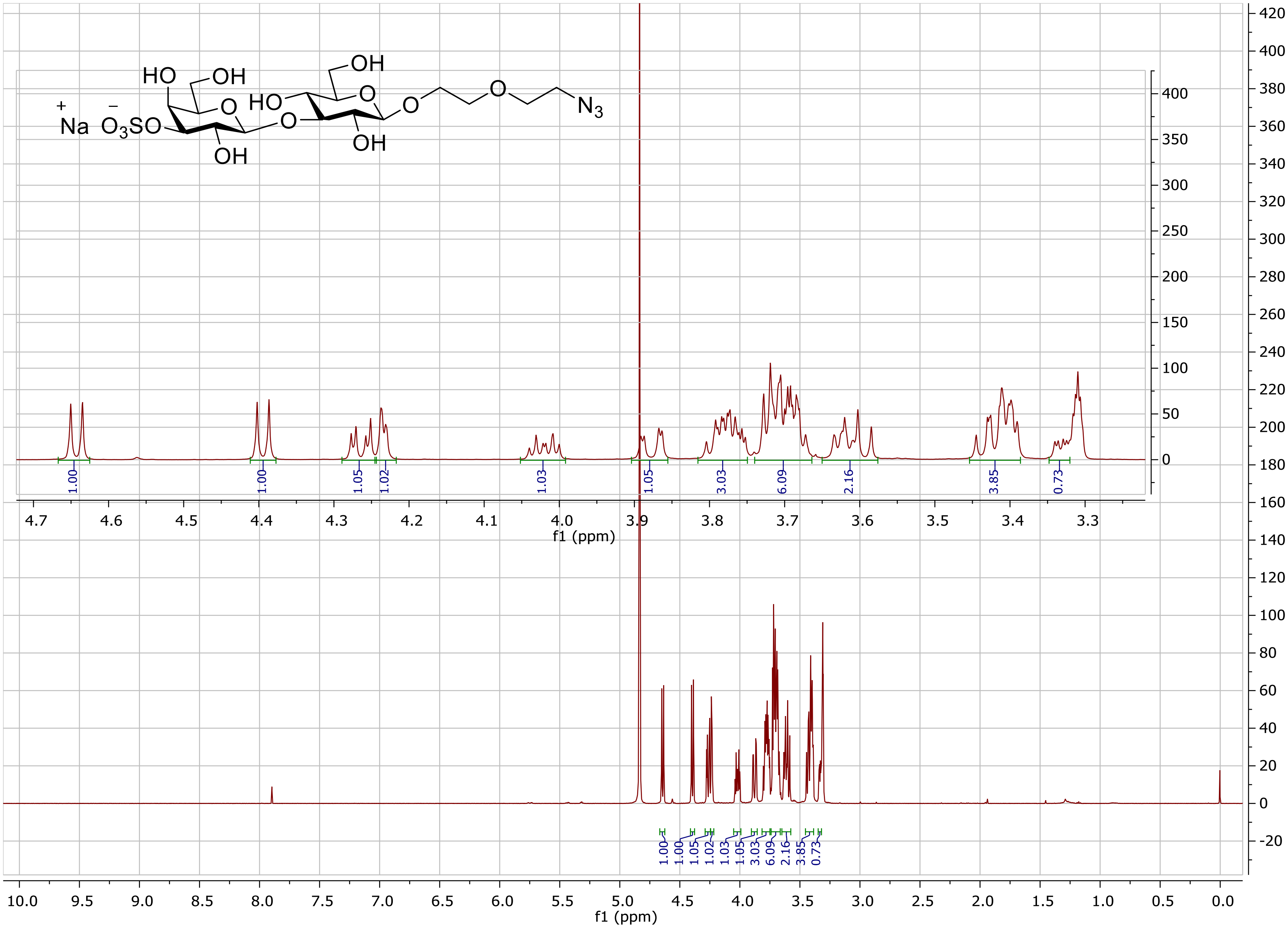

HO
OH
Na
O3SO
HO
OH
OH
N3
f1 (ppm)
f1 (ppm)

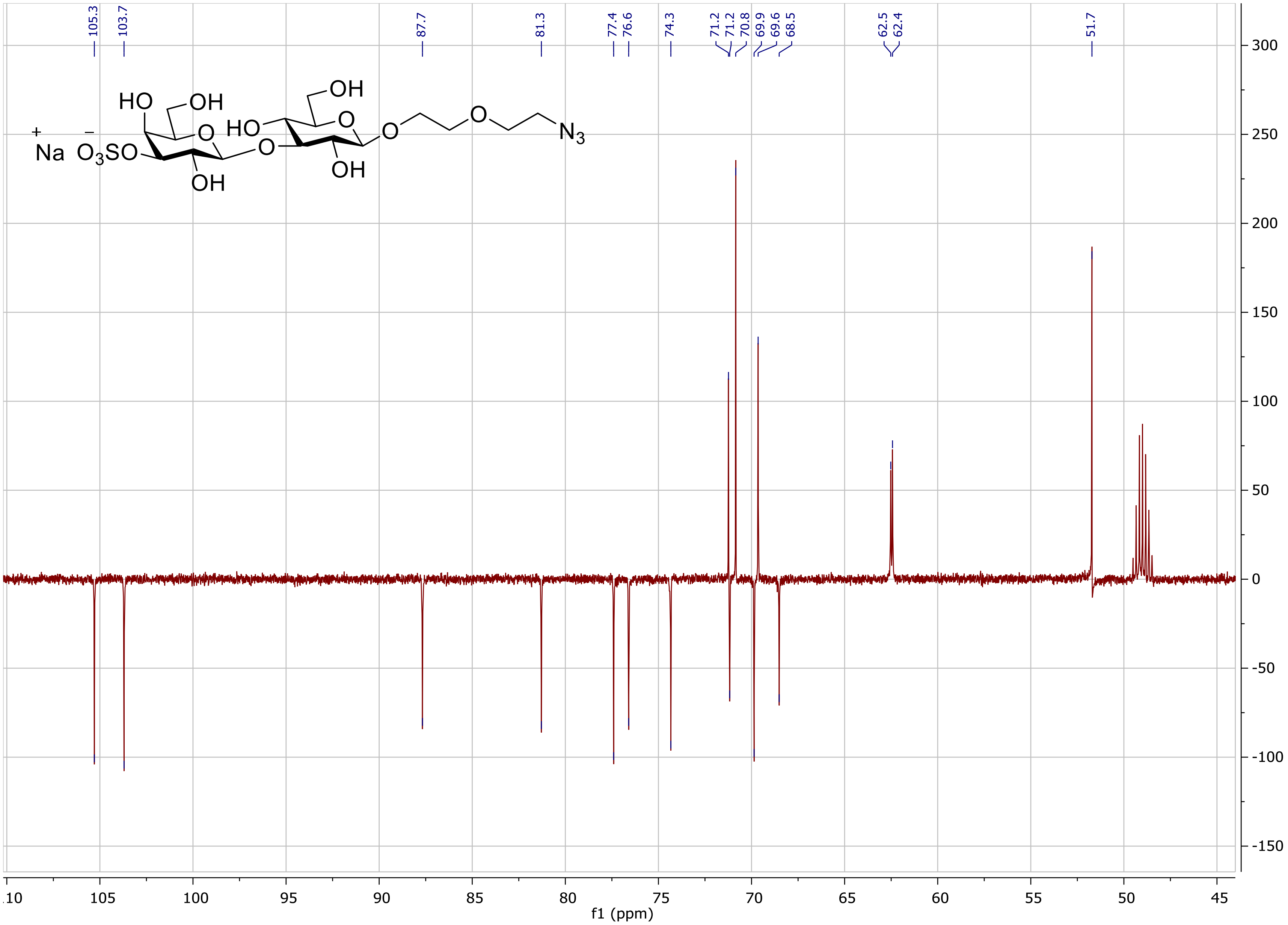

105.3
103.7
87.7
81.3
77.4
76.6
74.3
71.2
71.2
70.8
69.9
69.6
68.5
62.5
62.4
51.7
f1 (ppm)

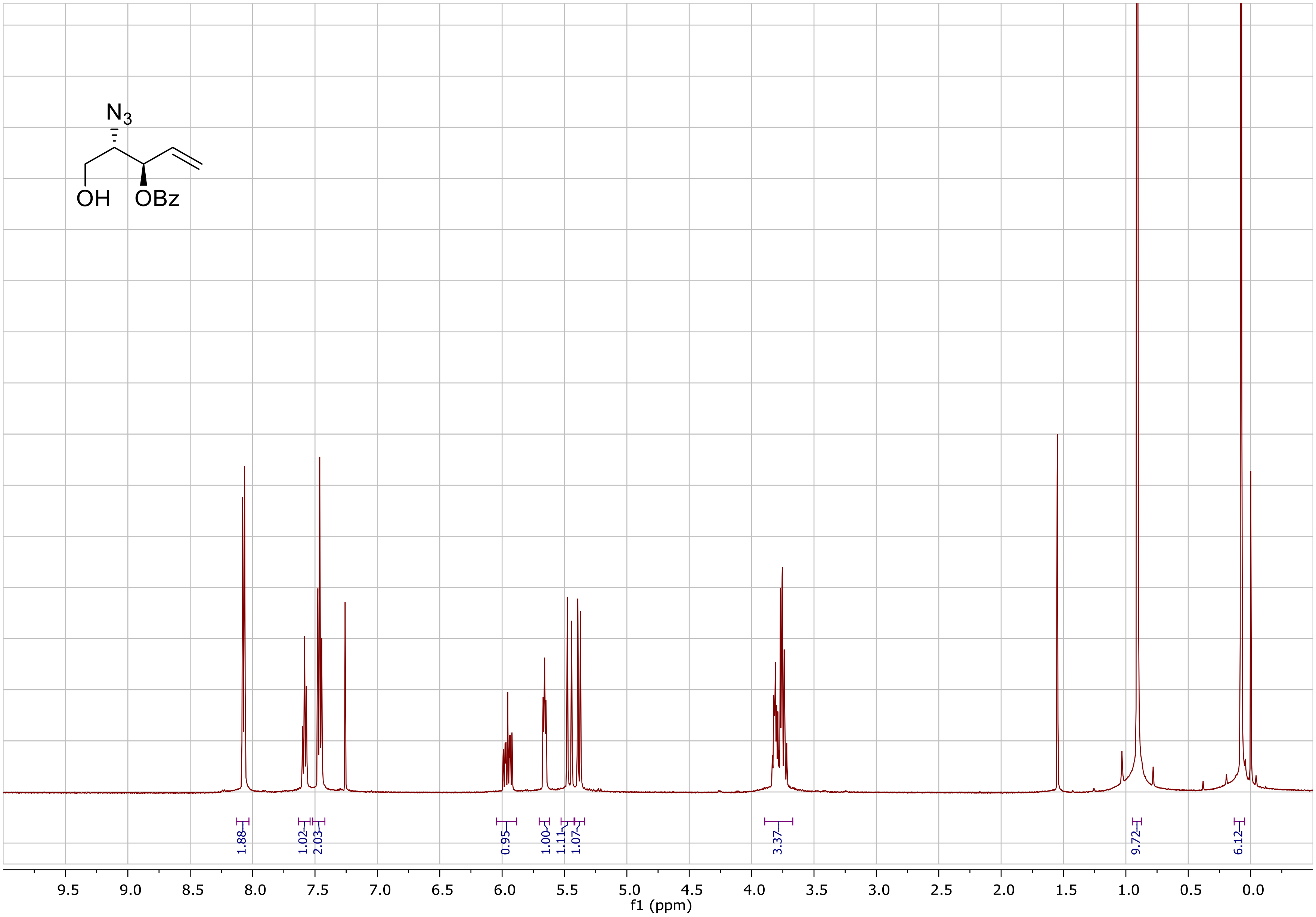
N3
OH
OBz
1.88
1.02
2.03
0.95
1.00
1.11
1.07
3.37
9.72
6.12
9.5 9.0 8.5 8.0 7.5 7.0 6.5 6.0 5.5 5.0 4.5 4.0 3.5 3.0 2.5 2.0 1.5 1.0 0.5 0.0
f1 (ppm)

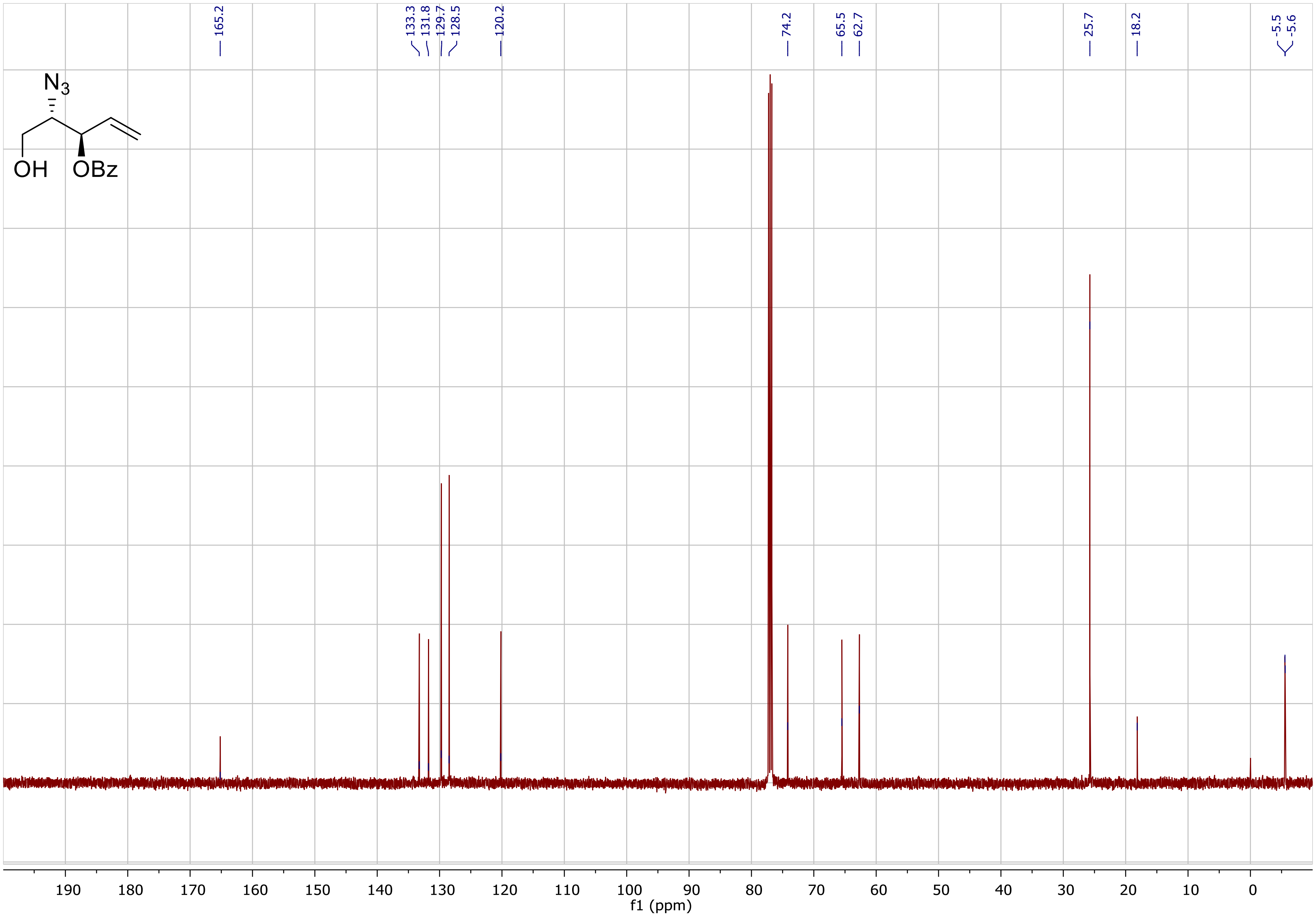
N3
OH
OBz
165.2
133.3
131.8
129.7
128.5
120.2
74.2
65.5
62.7
25.7
18.2
-5.5
-5.6
190
180
170
160
150
140
130
120
110
100
90
80
70
60
50
40
30
20
10
0
f1 (ppm)

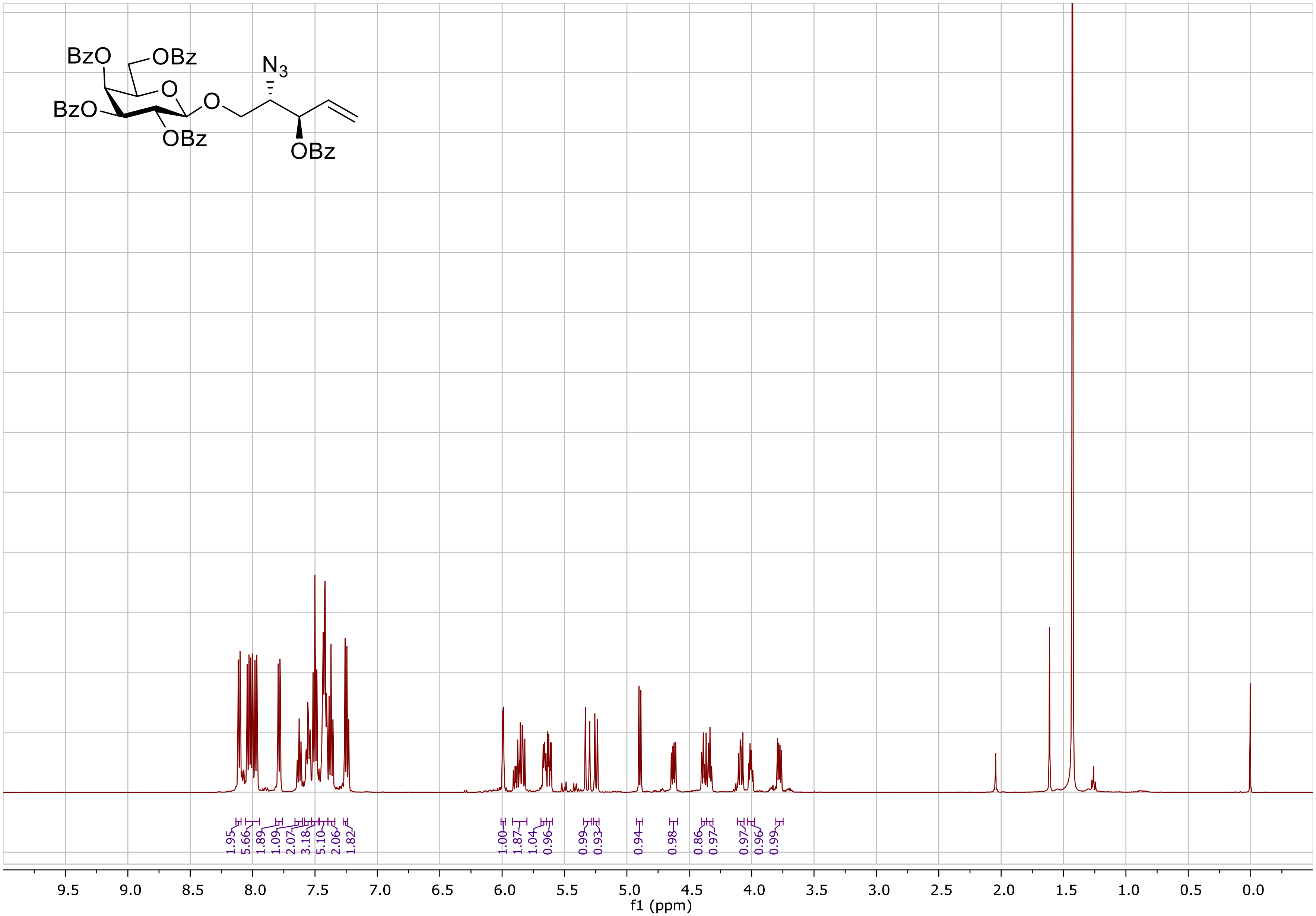
BzO
OBz
O
N3
BzO
O
OBz
OBz
1.95
5.66
1.89
1.09
2.07
3.18
5.10
2.06
1.82
1.00
1.87
1.04
0.96
0.99
0.93
0.94
0.98
0.86
0.97
0.97
0.96
0.99
9.5
9.0
8.5
8.0
7.5
7.0
6.5
6.0
5.5
5.0
4.5
4.0
3.5
3.0
2.5
2.0
1.5
1.0
0.5
0.0
f1 (ppm)

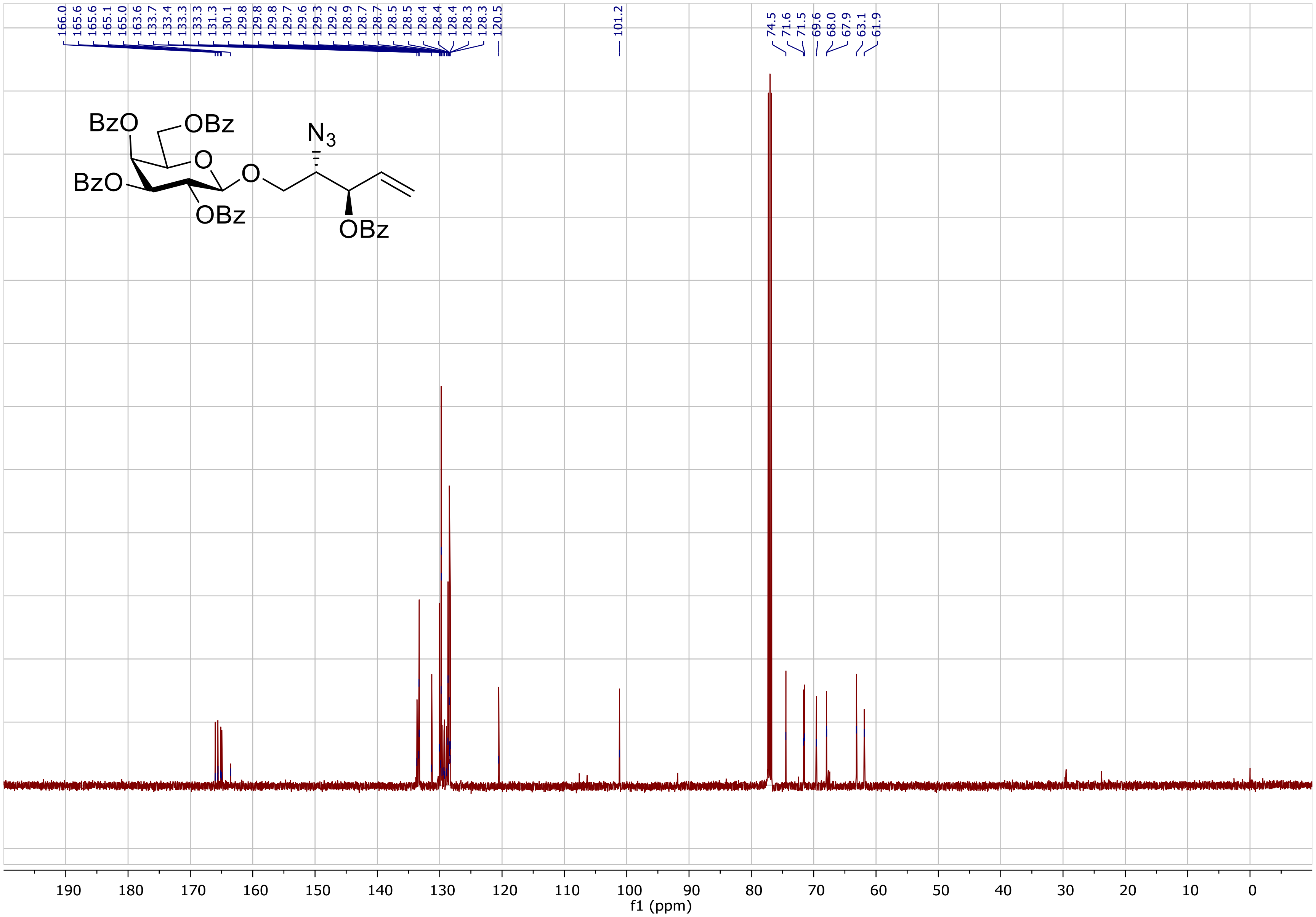

166.0
165.6
165.6
165.1
165.0
163.6
133.7
133.4
133.3
133.3
131.3
130.1
129.8
129.8
129.8
129.7
129.6
129.3
129.2
128.9
128.7
128.7
128.5
128.5
128.4
128.4
128.4
128.3
128.3
120.5
101.2
74.5
71.6
71.5
69.6
68.0
67.9
63.1
61.9
BzO
OBz
O
BzO
OBz
O
N3
OBz
190
180
170
160
150
140
130
120
110
100
90
80
70
60
50
40
30
20
10
0
f1 (ppm)

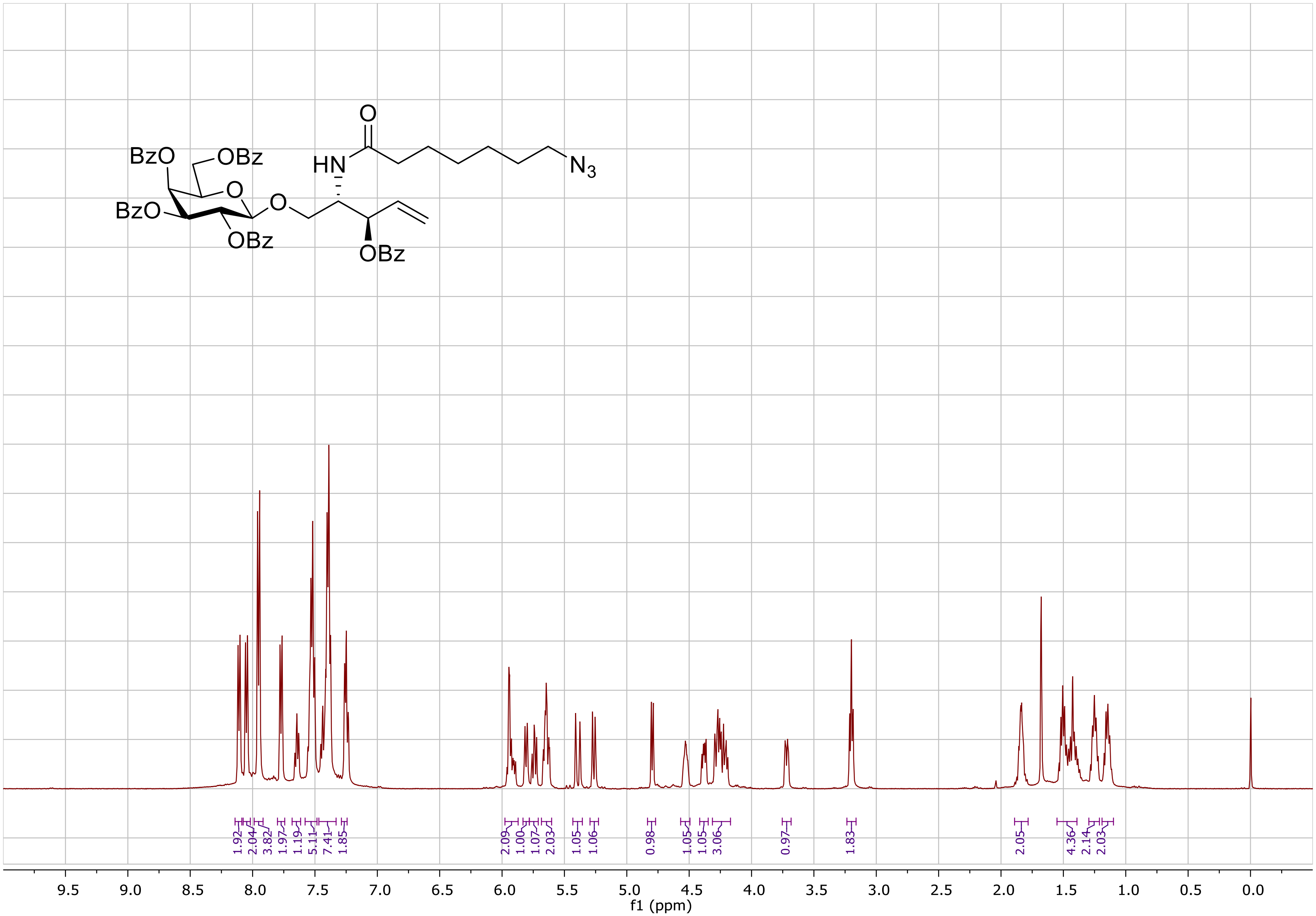

BzO
OBz
O
BzO
O
OBz
HN
O
N3
OBz
1.92
2.04
3.82
1.97
1.19
5.11
7.41
1.85
2.09
1.00
1.07
2.03
1.05
1.06
0.98
1.05
1.05
3.06
0.97
1.83
2.05
4.36
2.14
2.03
9.5
9.0
8.5
8.0
7.5
7.0
6.5
6.0
5.5
5.0
4.5
4.0
3.5
3.0
2.5
2.0
1.5
1.0
0.5
0.0
f1 (ppm)

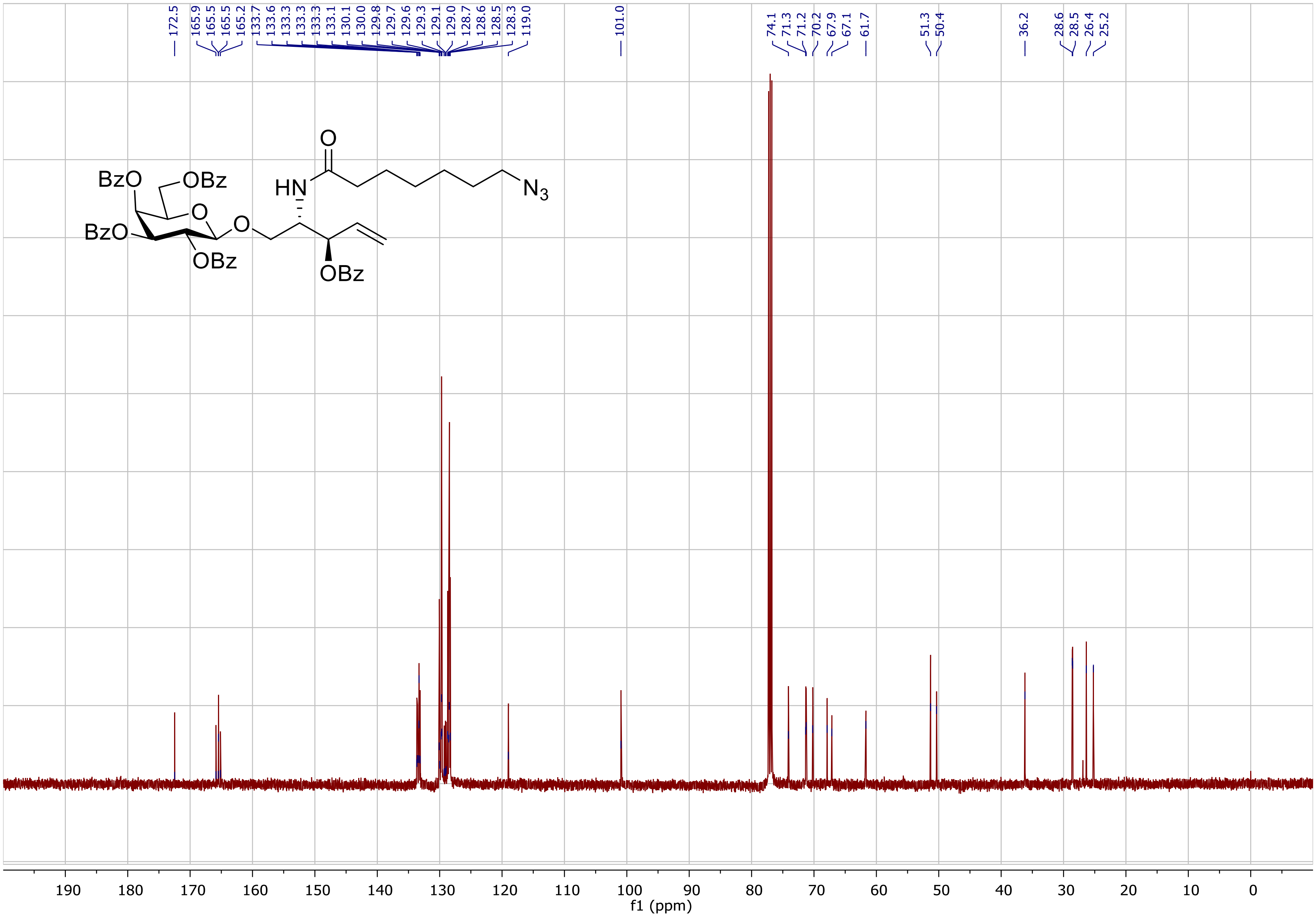

172.5
165.9
165.5
165.5
165.2
133.7
133.6
133.3
133.3
133.3
133.1
130.1
130.0
129.8
129.7
129.6
129.3
129.1
129.0
128.7
128.6
128.5
128.3
119.0
101.0
74.1
71.3
71.2
70.2
67.9
67.1
61.7
51.3
50.4
36.2
28.6
28.5
26.4
25.2
BzO
OBz
O
BzO
OBz
O
HN
O
N3
OBz
190
180
170
160
150
140
130
120
110
100
90
80
70
60
50
40
30
20
10
0
f1 (ppm)

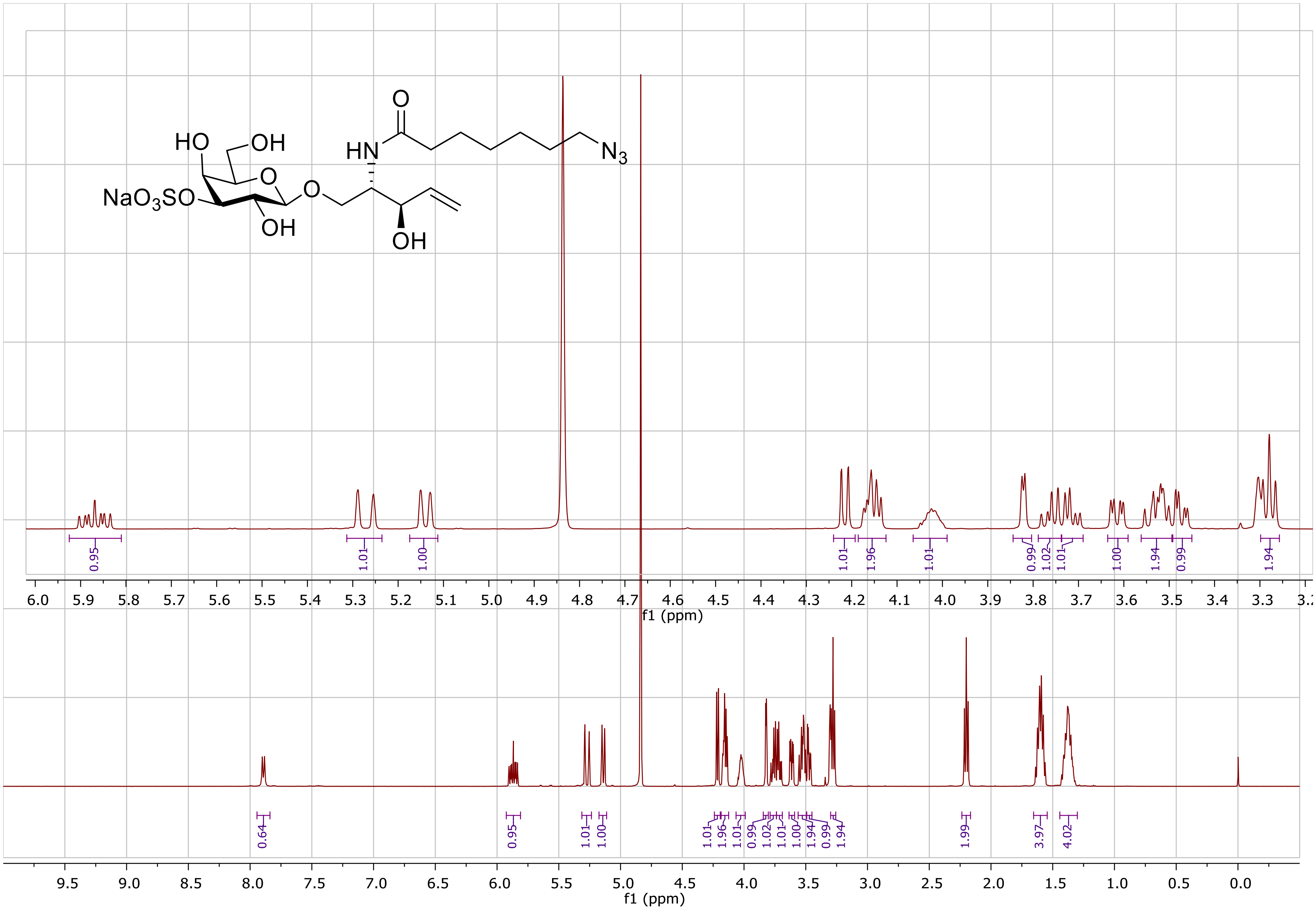

HO
OH
O
NaO3SO
OH
O
HN
O
N3
OH
f1 (ppm)
f1 (ppm)

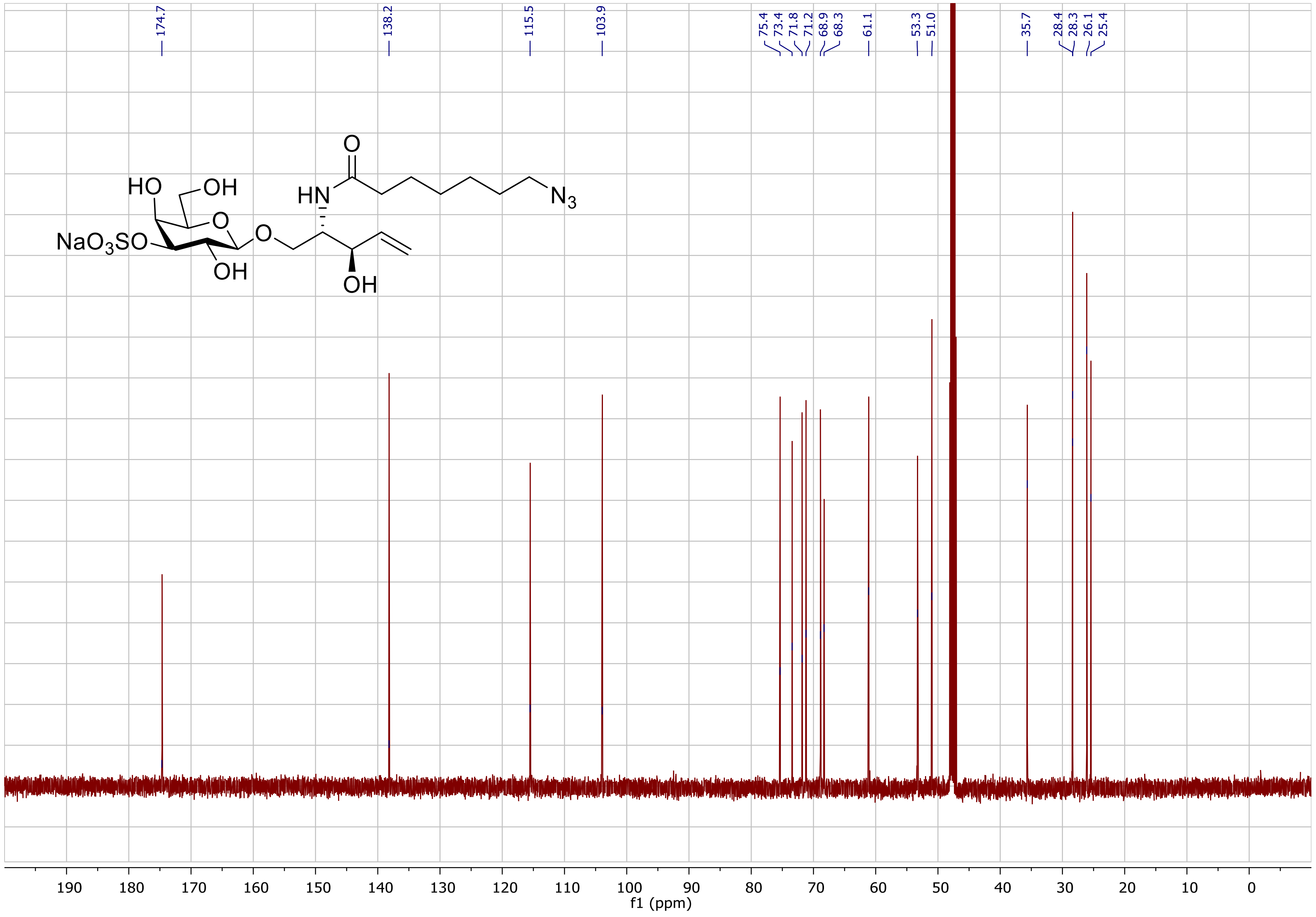
174.7
138.2
115.5
103.9
75.4
73.4
71.8
71.2
68.9
68.3
61.1
53.3
51.0
35.7
28.4
28.3
26.1
25.4
HO
OH
O
NaO3SO
OH
O
HN
O
N3
OH
190
180
170
160
150
140
130
120
110
100
90
80
70
60
50
40
30
20
10
0
f1 (ppm)

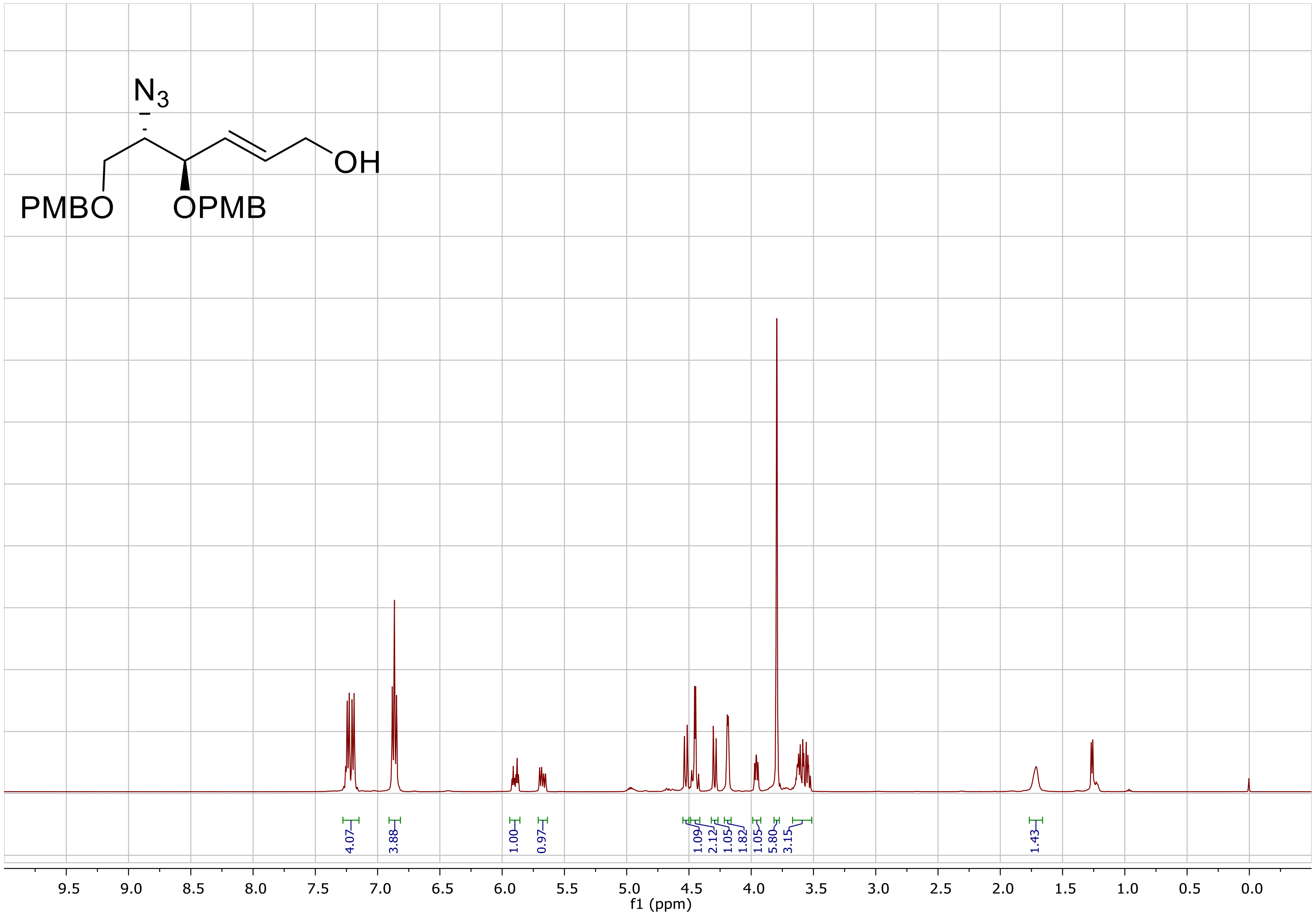

N3
OH
PMBO
OPMB
4.07
3.88
1.00
0.97
1.09
2.12
1.05
1.82
1.05
5.80
3.15
1.43
9.5
9.0
8.5
8.0
7.5
7.0
6.5
6.0
5.5
5.0
4.5
4.0
3.5
3.0
2.5
2.0
1.5
1.0
0.5
0.0
f1 (ppm)

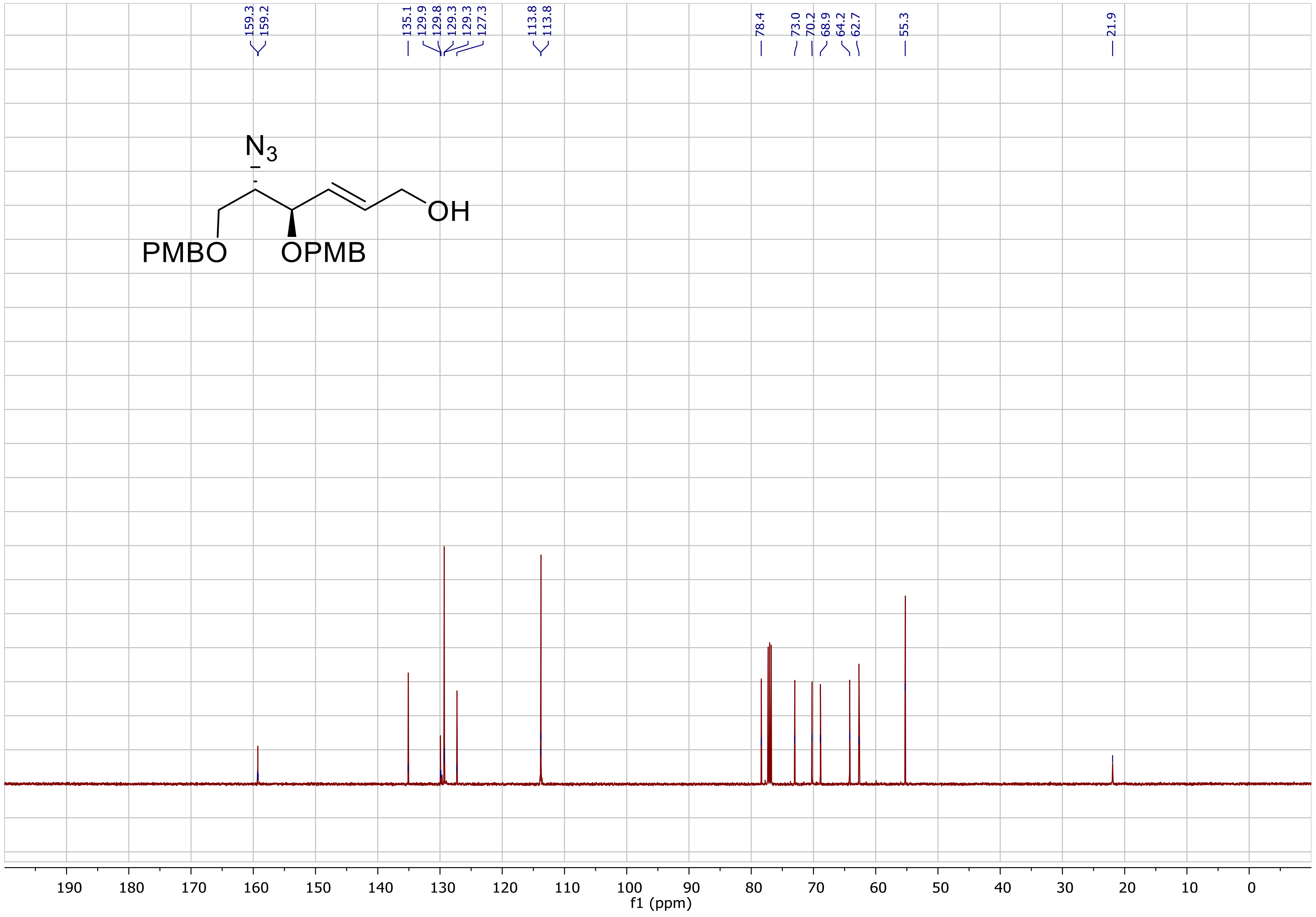

159.3
159.2
135.1
129.9
129.8
129.3
129.3
127.3
113.8
113.8
78.4
73.0
70.2
68.9
64.2
62.7
55.3
21.9
N3
OH
PMBO
OPMB
190 180 170 160 150 140 130 120 110 100 90 80 70 60 50 40 30 20 10 0
f1 (ppm)

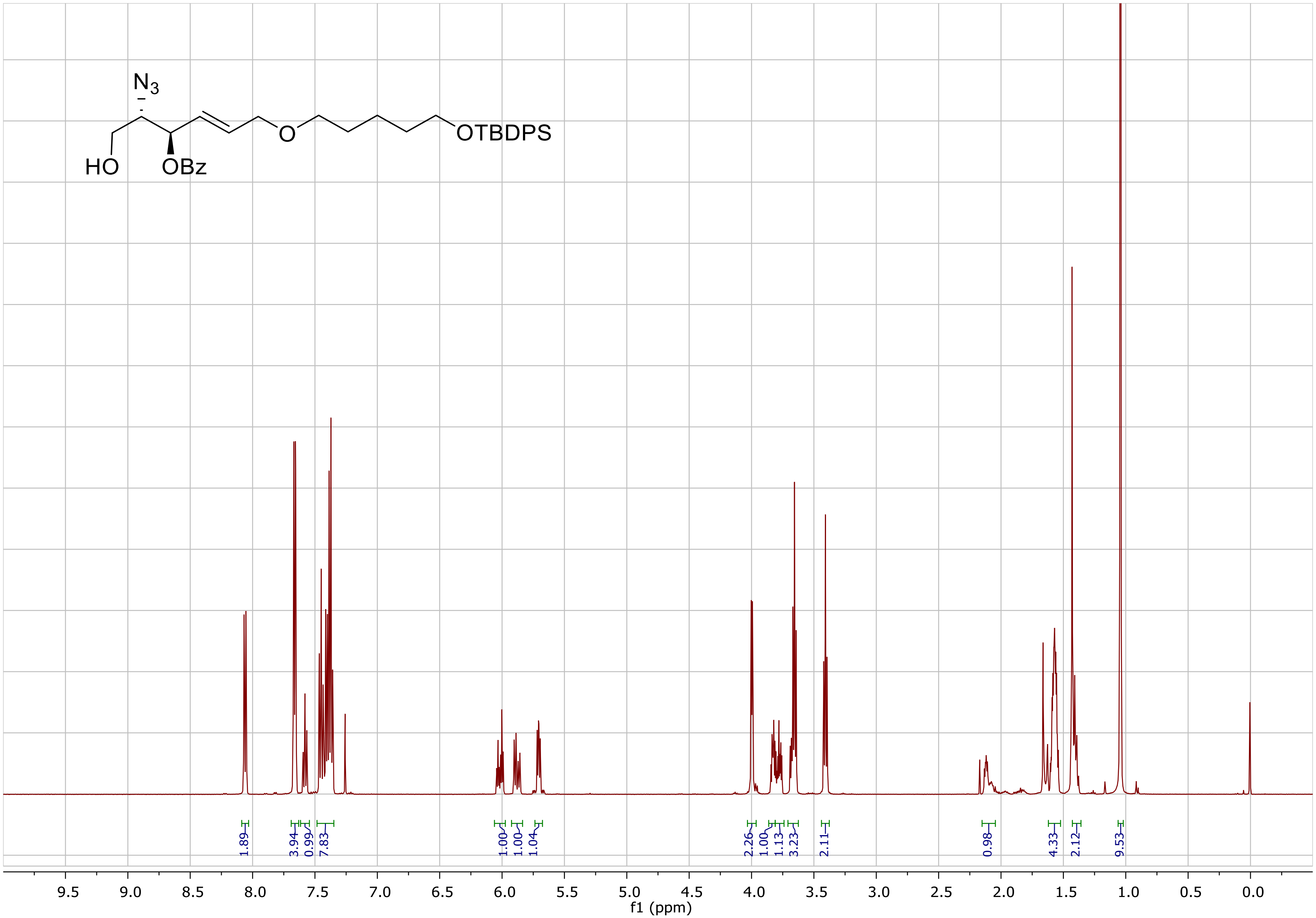

N3
HO
OBz
O
OTBDPS
1.89
3.94
0.99
7.83
1.00
1.00
1.04
2.26
1.00
1.13
3.23
2.11
0.98
4.33
2.12
9.53
9.5 9.0 8.5 8.0 7.5 7.0 6.5 6.0 5.5 5.0 4.5 4.0 3.5 3.0 2.5 2.0 1.5 1.0 0.5 0.0
f1 (ppm)

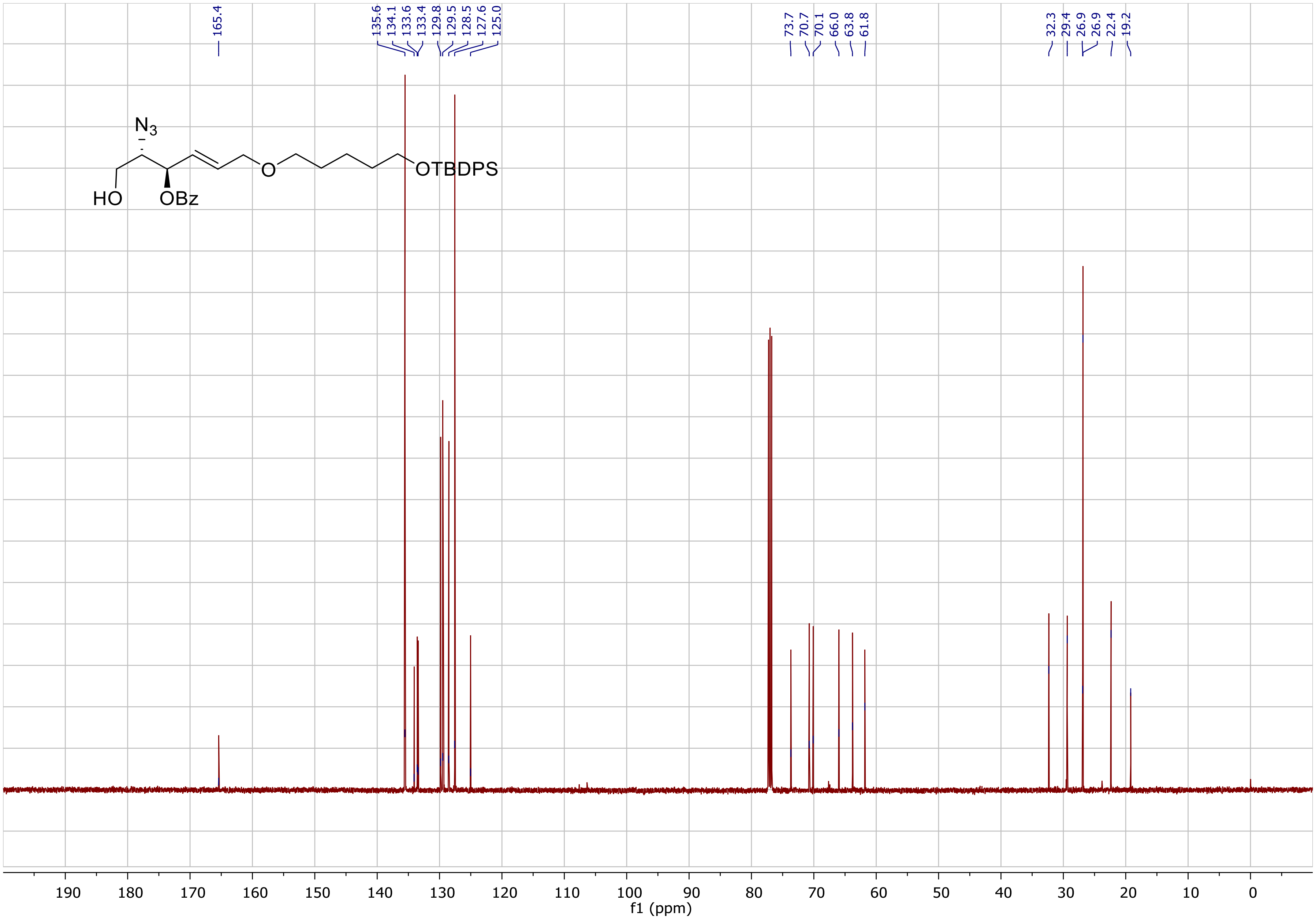

165.4
135.6
134.1
133.6
133.4
129.8
129.5
128.5
127.6
125.0
73.7
70.7
70.1
66.0
63.8
61.8
32.3
29.4
26.9
26.9
22.4
19.2
N3
HO
OBz
O
OTBDPS
190
180
170
160
150
140
130
120
110
100
90
80
70
60
50
40
30
20
10
0
f1 (ppm)

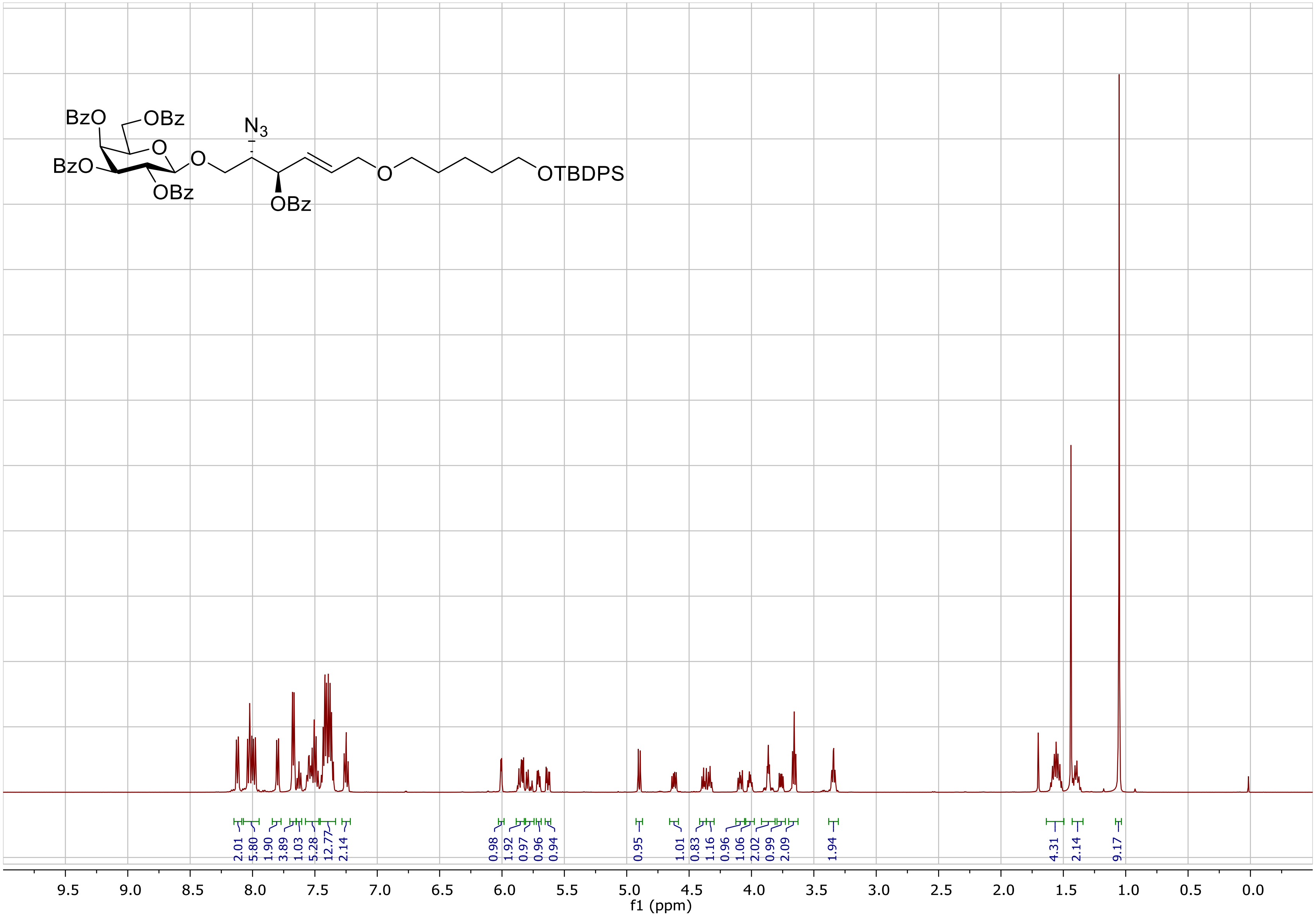
BzO
OBz
O
BzO
OBz
O
N_3
OBz
O
OTBDPS
2.01
5.80
1.90
3.89
1.03
5.28
12.77
2.14
0.98
1.92
0.97
0.96
0.94
0.95
1.01
0.83
1.16
0.96
1.06
2.02
0.99
2.09
1.94
4.31
2.14
9.17
9.5
9.0
8.5
8.0
7.5
7.0
6.5
6.0
5.5
5.0
4.5
4.0
3.5
3.0
2.5
2.0
1.5
1.0
0.5
0.0
f1 (ppm)

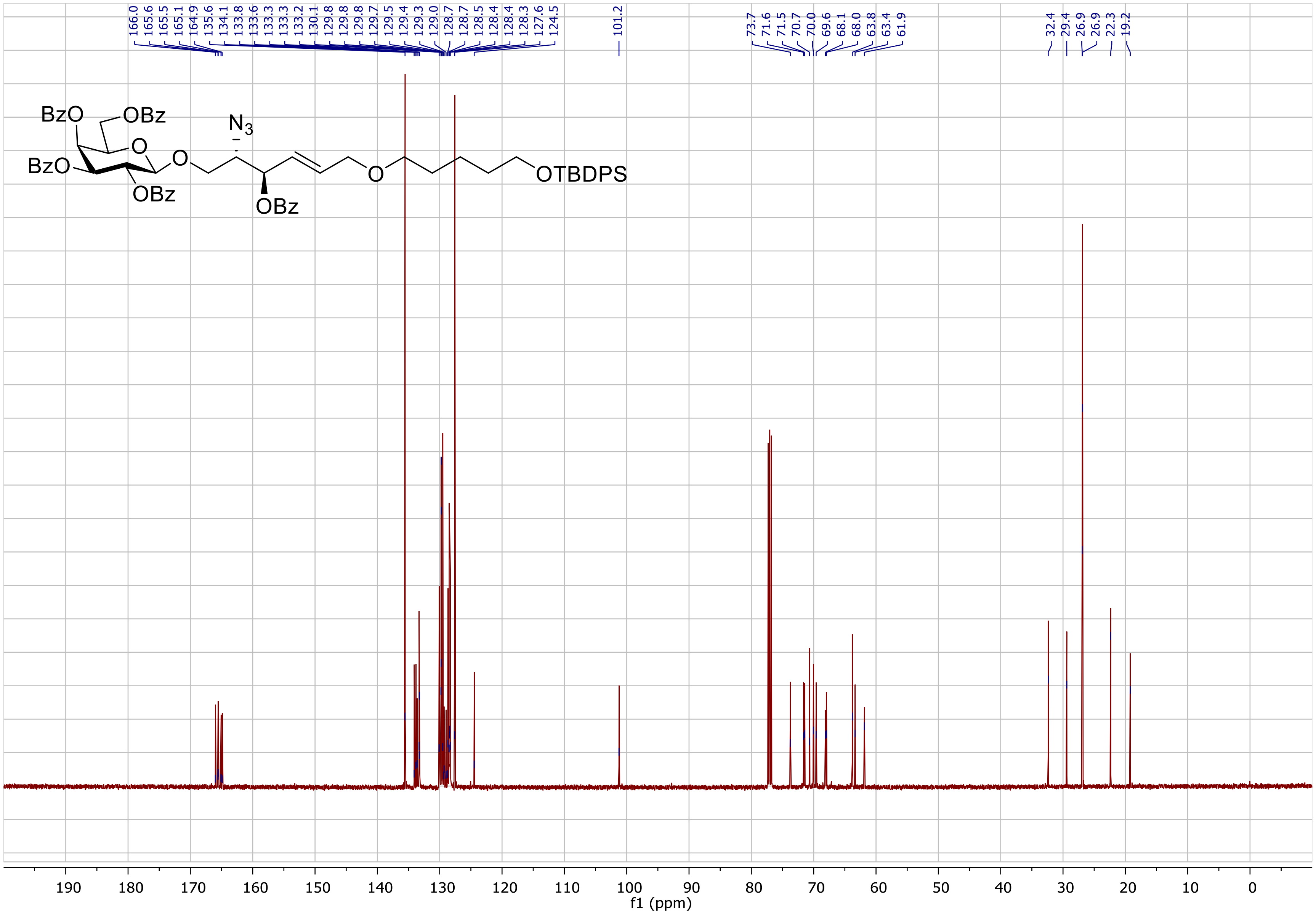

166.0
165.6
165.5
165.1
164.9
135.6
134.1
133.8
133.6
133.3
133.3
133.2
130.1
129.8
129.8
129.8
129.7
129.5
129.4
129.3
129.0
128.7
128.7
128.5
128.4
128.4
128.3
127.6
124.5
101.2
73.7
71.6
71.5
70.7
70.0
69.6
68.1
68.0
63.8
63.4
61.9
32.4
29.4
26.9
26.9
22.3
19.2
BzO
OBz
O
N3
BzO
O
O
OTBDPS
OBz
OBz
190
180
170
160
150
140
130
120
110
100
90
80
70
60
50
40
30
20
10
0
f1 (ppm)

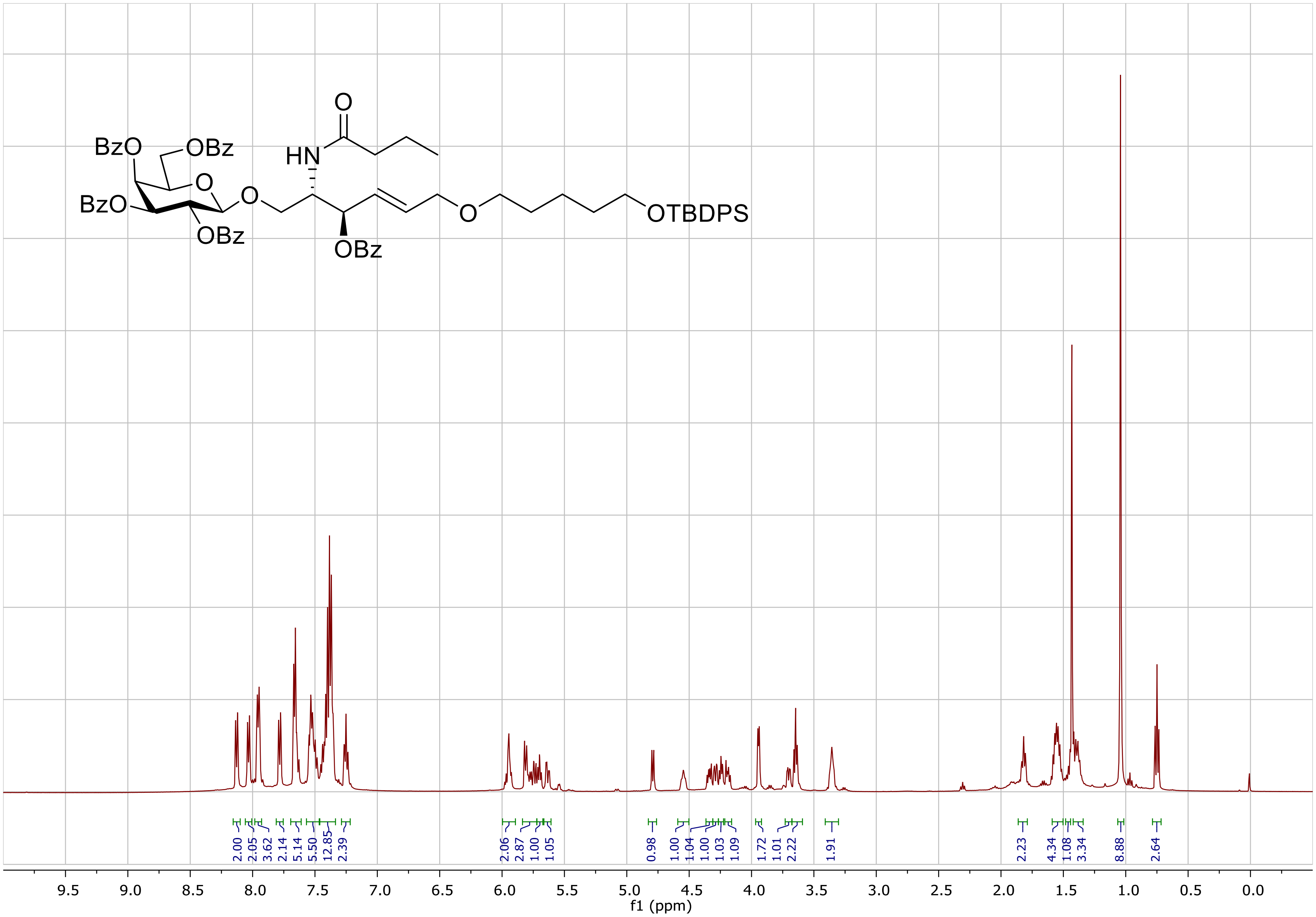

BzO
OBz
O
BzO
OBz
O
HN
O
OBz
O
OTBDPS
2.00
2.05
3.62
2.14
5.14
5.50
12.85
2.39
2.06
2.87
1.00
1.05
0.98
1.00
1.04
1.00
1.03
1.09
1.72
1.01
2.22
1.91
2.23
4.34
1.08
3.34
8.88
2.64
9.5
9.0
8.5
8.0
7.5
7.0
6.5
6.0
5.5
5.0
4.5
4.0
3.5
3.0
2.5
2.0
1.5
1.0
0.5
0.0
f1 (ppm)

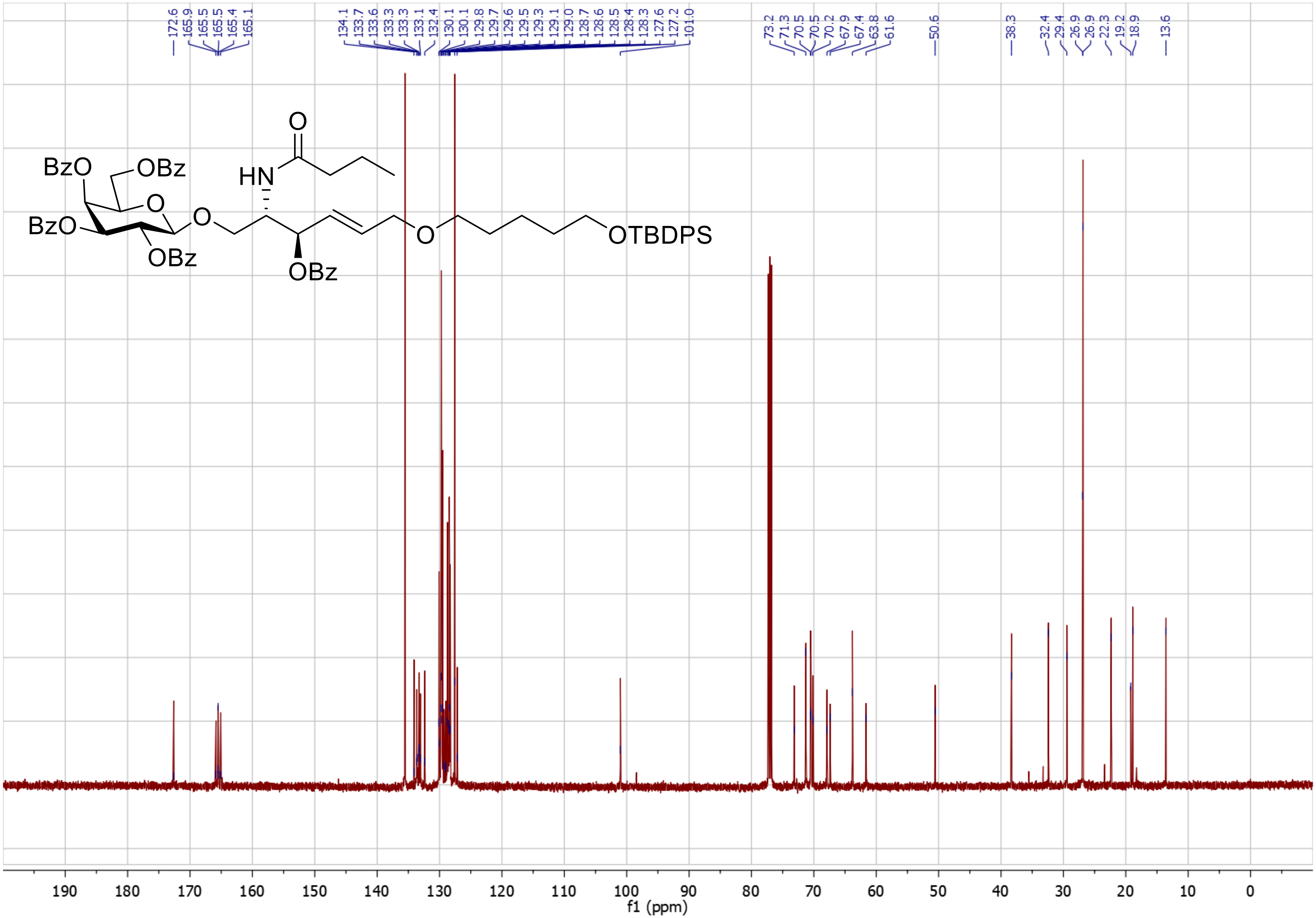

172.6
165.9
165.5
165.5
165.4
165.1
134.1
133.7
133.6
133.3
133.3
133.1
132.4
130.1
130.1
129.8
129.7
129.6
129.5
129.3
129.1
129.0
128.7
128.6
128.5
128.4
128.3
127.6
127.2
101.0
73.2
71.3
70.5
70.5
70.2
67.9
67.4
63.8
61.6
50.6
38.3
32.4
29.4
26.9
26.9
22.3
19.2
18.9
13.6
BzO
OBz
O
BzO
OBz
O
HN
O
OBz
O
OTBDPS
190
180
170
160
150
140
130
120
110
100
90
80
70
60
50
40
30
20
10
0
f1 (ppm)

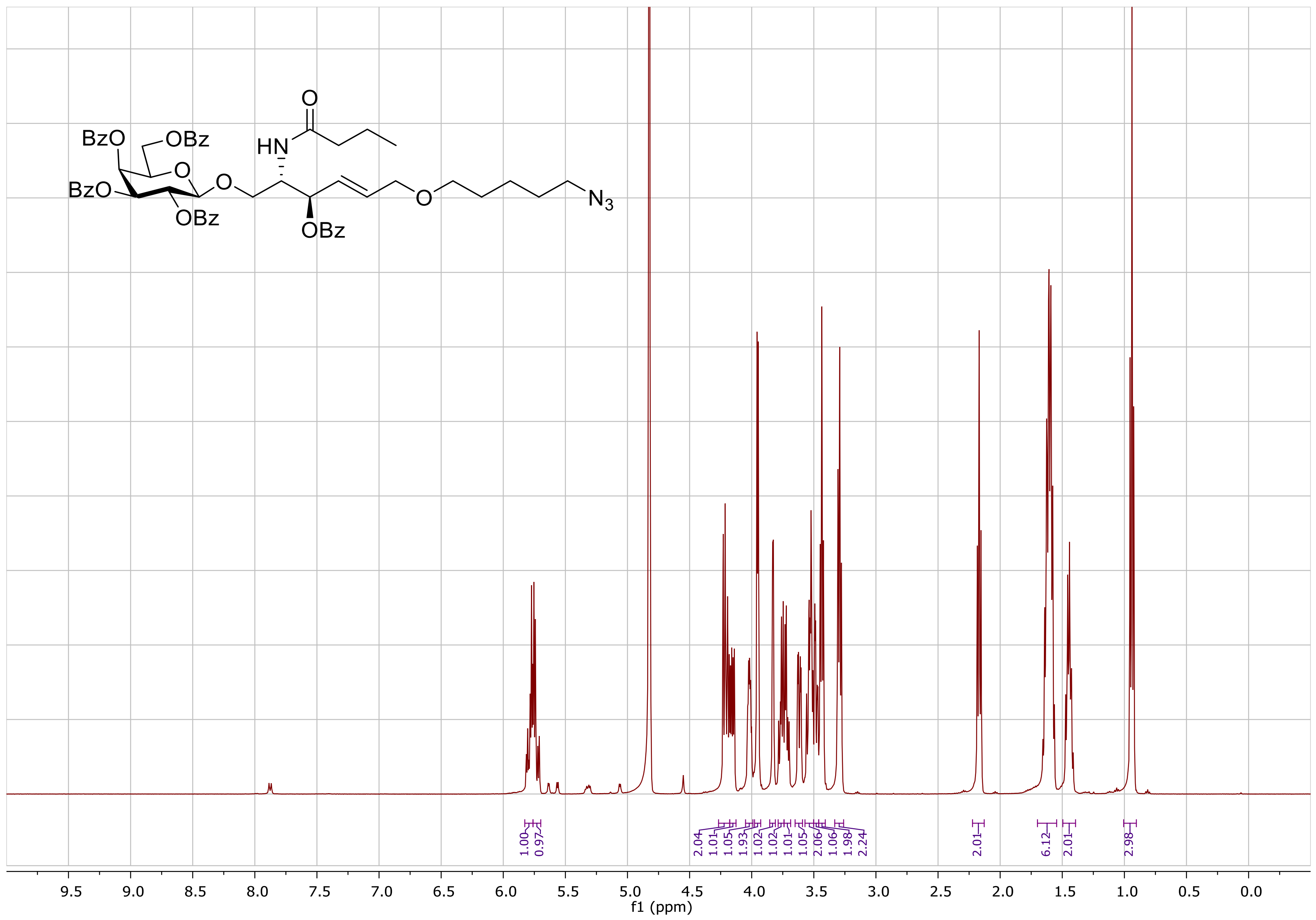

BzO
OBz
O
BzO
OBz
O
HN
O
OBz
O
N3
1.00
0.97
2.04
1.01
1.05
1.93
1.02
1.02
1.01
1.05
2.06
1.06
1.98
2.24
2.01
6.12
2.01
2.98
9.5
9.0
8.5
8.0
7.5
7.0
6.5
6.0
5.5
5.0
4.5
4.0
3.5
3.0
2.5
2.0
1.5
1.0
0.5
0.0
f1 (ppm)

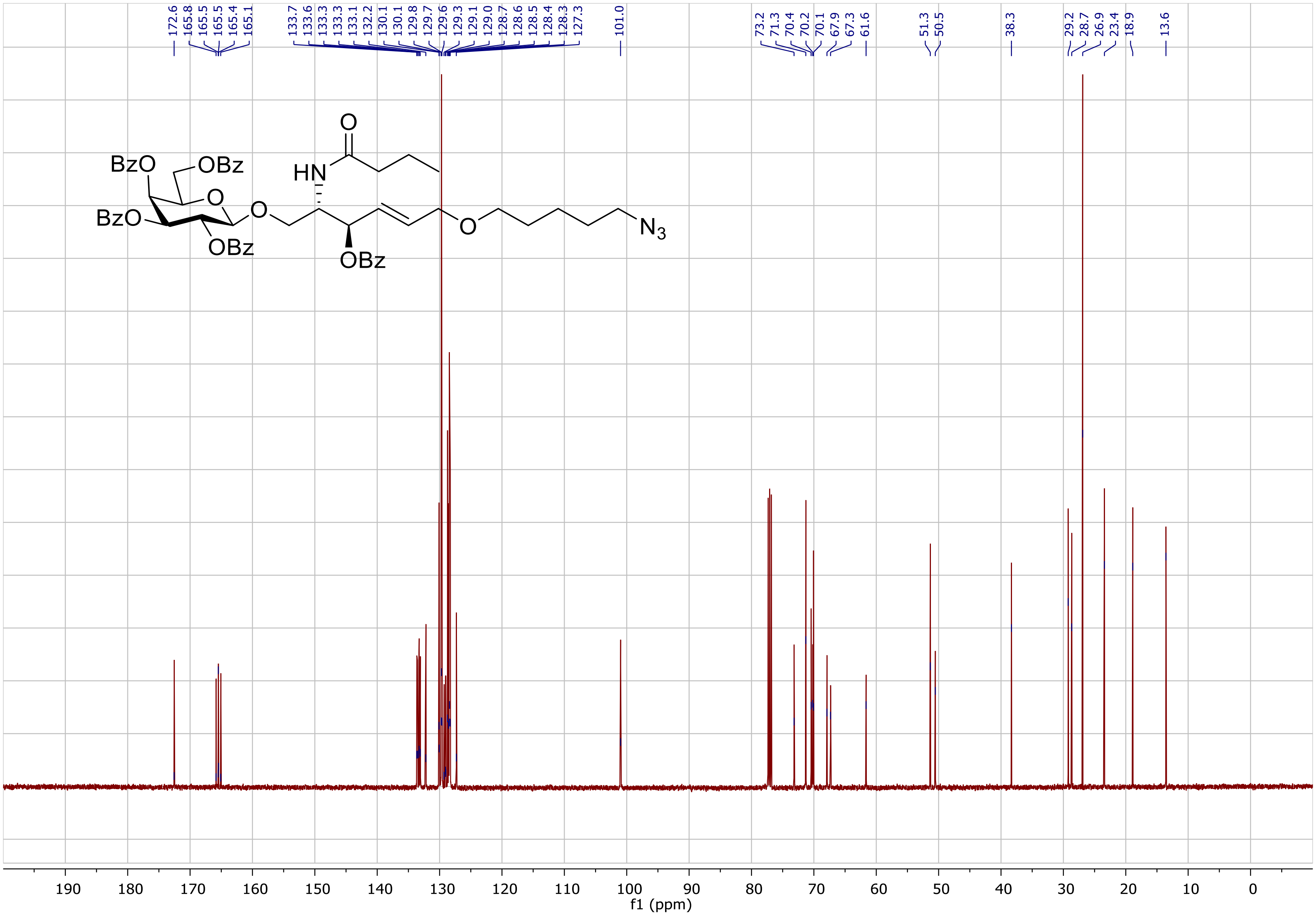
172.6
165.8
165.5
165.5
165.4
165.1
133.7
133.6
133.3
133.3
133.1
132.2
130.1
130.1
129.8
129.7
129.6
129.3
129.1
129.0
128.7
128.6
128.5
128.4
128.3
127.3
101.0
73.2
71.3
70.4
70.2
70.1
67.9
67.3
61.6
51.3
50.5
38.3
29.2
28.7
26.9
23.4
18.9
13.6
BzO
OBz
O
BzO
OBz
O
HN
O
OBz
O
N3
190 180 170 160 150 140 130 120 110 100 90 80 70 60 50 40 30 20 10 0
f1 (ppm)

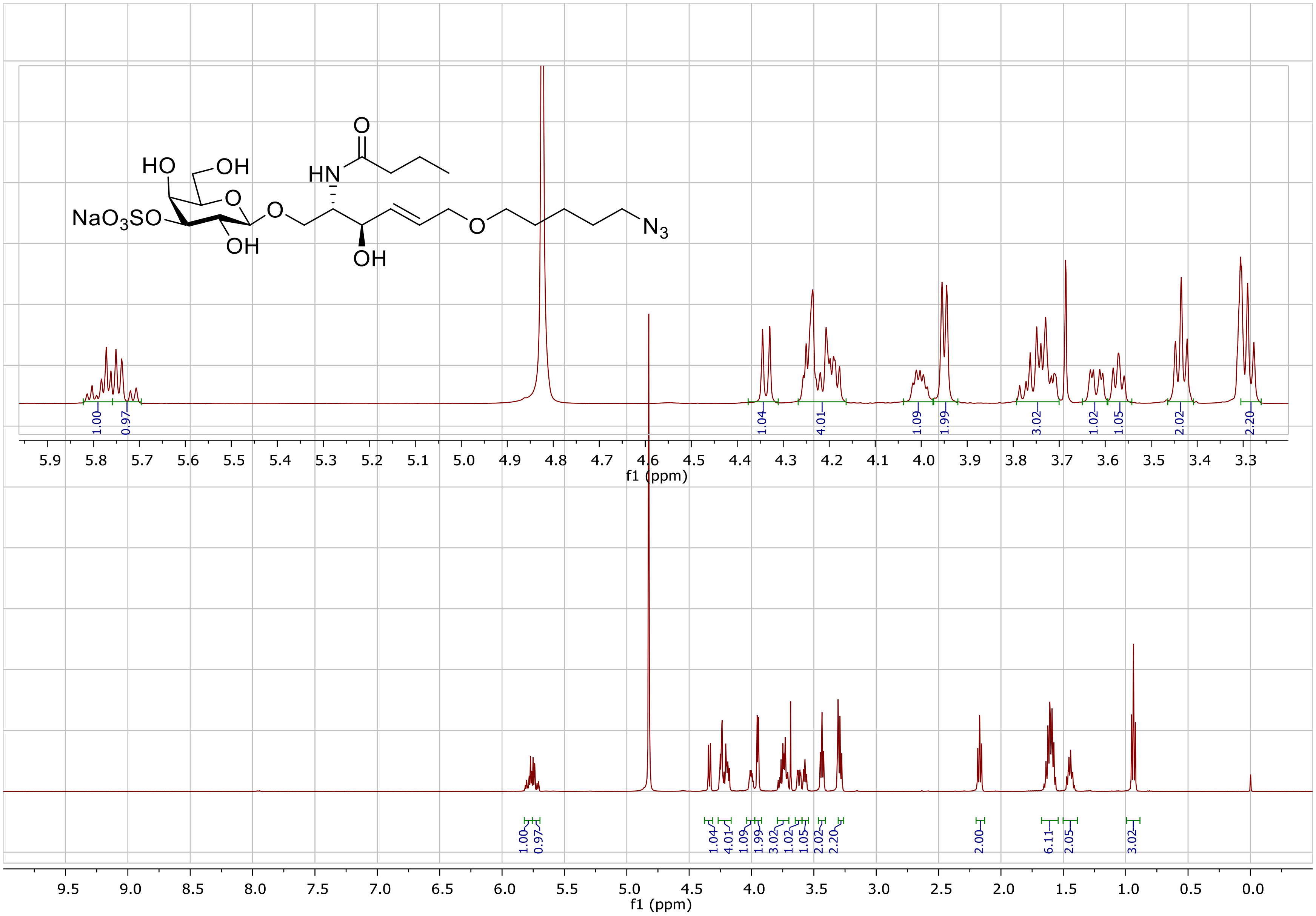
HO
OH
O
NaO3SO
OH
O
HN
O
OH
O
N3
1.00
0.97
1.04
4.01
1.09
1.99
3.02
1.02
1.05
2.02
2.20
f1 (ppm)
2.00
6.11
2.05
3.02
f1 (ppm)

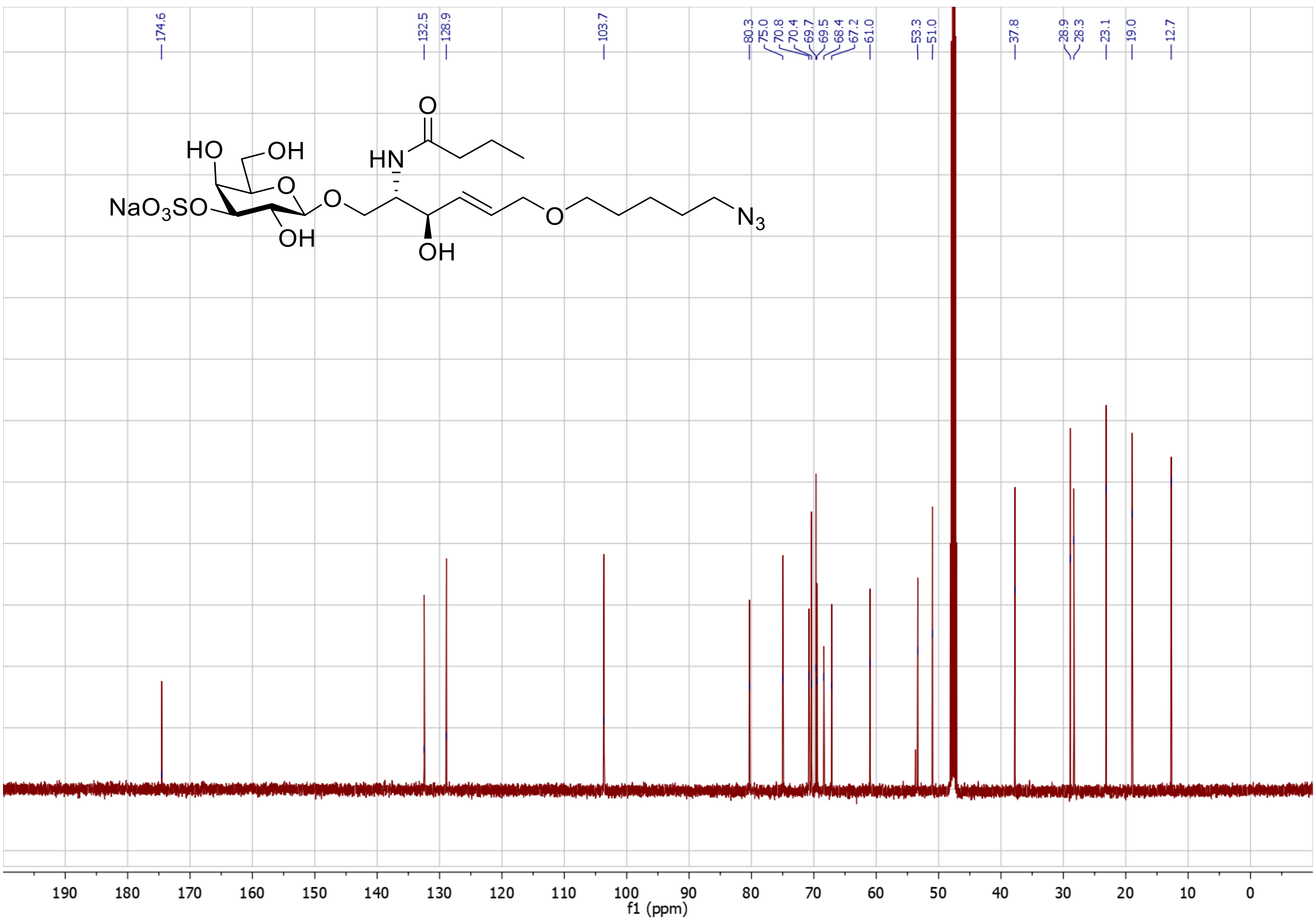

174.6
132.5
128.9
103.7
80.3
75.0
70.8
70.4
69.7
69.5
68.4
67.2
61.0
53.3
51.0
37.8
28.9
28.3
23.1
19.0
12.7
HO
OH
O
NaO3SO
OH
O
HN
O
OH
O
N3
190
180
170
160
150
140
130
120
110
100
90
80
70
60
50
40
30
20
10
0
f1 (ppm)
